# Antiplasmodial, antimalarial activities and toxicity of African medicinal plants: a systematic review of literature

**DOI:** 10.1186/s12936-021-03866-0

**Published:** 2021-08-25

**Authors:** Elahe Tajbakhsh, Tebit Emmanuel Kwenti, Parya Kheyri, Saeed Nezaratizade, David S. Lindsay, Faham Khamesipour

**Affiliations:** 1 Department of Microbiology, Shahrekord Branch, Islamic Azad University, Shahrekord, Iran; 2 Department of Biomedical Science, Faculty of Health Sciences, Regional Hospital Buea, Buea, Cameroon; 3 Department of Public Health and Hygiene, Faculty of Health Sciences, University of Buea, Yaoundé, Cameroon; 4 Department of Medical Laboratory Sciences, Faculty of Health Sciences, University of Buea, Yaoundé, Cameroon; 5 Young Researchers and Elite Club, Shahrekord Branch, Islamic Azad University, Shahrekord, Iran; 6 Department of Biomedical Sciences and Pathobiology, Center for One Health Research, Virginia Maryland College of Veterinary Medicine, Virginia Tech, 1410 Prices Fork Road, Blacksburg, VA 24061-0342 USA; 7 Shahrekord Branch, Islamic Azad University, Shahrekord, Iran; 8 Shahid Beheshti University of Medical Sciences, Tehran, Iran

**Keywords:** Malaria, Medicinal plants, Antiplasmodial activity, Antimalarial activity

## Abstract

**Background:**

Malaria still constitutes a major public health menace, especially in tropical and subtropical countries. Close to half a million people mainly children in Africa, die every year from the disease. With the rising resistance to frontline drugs (artemisinin-based combinations), there is a need to accelerate the discovery and development of newer anti-malarial drugs. A systematic review was conducted to identify the African medicinal plants with significant antiplasmodial and/or anti-malarial activity, toxicity, as wells as assessing the variation in their activity between study designs (in vitro and in vivo).

**Methods:**

Key health-related databases including Google Scholar, PubMed, PubMed Central, and Science Direct were searched for relevant literature on the antiplasmodial and anti-malarial activities of African medicinal plants.

**Results:**

In total, 200 research articles were identified, a majority of which were studies conducted in Nigeria. The selected research articles constituted 722 independent experiments evaluating 502 plant species. Of the 722 studies, 81.9%, 12.4%, and 5.5% were in vitro, in vivo*,* and combined in vitro and in vivo*,* respectively. The most frequently investigated plant species were *Azadirachta indica, Zanthoxylum chalybeum, Picrilima nitida,* and *Nauclea latifolia* meanwhile *Fabaceae, Euphorbiaceae, Annonaceae, Rubiaceae, Rutaceae, Meliaceae,* and *Lamiaceae* were the most frequently investigated plant families. Overall, 248 (34.3%), 241 (33.4%), and 233 (32.3%) of the studies reported very good, good, and moderate activity, respectively. *Alchornea cordifolia, Flueggea virosa, Cryptolepis sanguinolenta, Zanthoxylum chalybeum,* and *Maytenus senegalensis* gave consistently very good activity across the different studies. In all, only 31 (4.3%) of studies involved pure compounds and these had significantly (p = 0.044) higher antiplasmodial activity relative to crude extracts. Out of the 198 plant species tested for toxicity, 52 (26.3%) demonstrated some degree of toxicity, with toxicity most frequently reported with *Azadirachta indica* and *Vernonia amygdalina*. These species were equally the most frequently inactive plants reported. The leaves were the most frequently reported toxic part of plants used. Furthermore, toxicity was observed to decrease with increasing antiplasmodial activity.

**Conclusions:**

Although there are many indigenous plants with considerable antiplasmodial and anti-malarial activity, the progress in the development of new anti-malarial drugs from African medicinal plants is still slothful, with only one clinical trial with *Cochlospermum planchonii* (*Bixaceae*) conducted to date. There is, therefore, the need to scale up anti-malarial drug discovery in the African region.

**Supplementary Information:**

The online version contains supplementary material available at 10.1186/s12936-021-03866-0.

## Background

Malaria still constitutes a major public health menace, especially in tropical and subtropical countries. Various species of *Plasmodium*, transmitted through the bite of an infected female *Anopheles* mosquito, cause malaria, including *Plasmodium falciparum, Plasmodium malariae, Plasmodium ovale, Plasmodium vivax,* and *Plasmodium knowlesi*. Among these species, *P. falciparum* is the most virulent, responsible for the highest morbidity and mortality. It is also the predominant species in sub-Saharan Africa (SSA), a region with the highest number of malaria cases and deaths in the world. According to the World Health Organization (WHO), there were 228 million cases, and 405,000 malaria attributed deaths in 2018 [1]. In SSA, children and pregnant women are the most at-risk groups [1–3].

Malaria can be treated using chemotherapy but there is widespread resistance to many of the drugs. The first case of resistance to artemisinins was reported in Cambodia in 2006 and has then spread to most of South-East Asia [4, 5]. The safety of chemoprophylaxis is also a major concern; for instance, primaquine, atovaquone, and doxycycline are contraindicated in pregnant women and children [6]. All these shortcomings necessitate the discovery and production of new drugs to treat malaria.

In the past 50 years, natural compounds including plant products, have played a major role in drug discovery and have provided value to the pharmaceutical industry [7]. For instance, therapeutics for various infectious diseases, cancer, and other debilitation diseases caused by metabolic disorders have all benefitted from many drug classes that were initially developed based on active compounds from plant sources [8]. Furthermore, quinine and artemisinin, and their synthetic derivatives which are the mainstay of anti-malarial chemotherapy, were also derived from plant sources. In malaria-endemic areas, especially in Africa, many people rely on herbal medicines as the first line of treatment [9]. The common reasons for their preference vary from the cost of standard drugs, availability and accessibility, perceived effectiveness, low side effect, and faith in traditional medicines [10].

Reviews of the antiplasmodial and anti-malarial activities of medicinal plants are needed to drive research into the discovery and production of new anti-malarial drugs. Only a few reviews of the antiplasmodial or anti-malarial activity of medicinal plants have been published in the scientific literature [11–16]. These reviews focused only on studies with high antiplasmodial or anti-malarial activity and hardly report on their toxicity. The purpose of this study was to review medicinal plants with moderate to very good antiplasmodial and anti-malarial activities, as well as assess the variation in the activities between different methods. Furthermore, the toxicity of plant species is highlighted.

## Methods

The literature was reviewed in search of scientific articles reporting antiplasmodial activities (IC_50_, ED_50_, LD_50_, and parasite suppression rate) of medicinal plants used in Africa to treat malaria. The current study conforms to the Preferred Reporting Items for Systematic Reviews and Meta-analysis (PRISMA) guidelines [17].

### Search strategy and selection criteria

Relevant articles were searched in health-related electronic databases including PubMed, PubMed Central, Google Scholar, and ScienceDirect using the keywords: Traditional herbs or Medicinal plants or Antiplasmodial activity or Antimalarial activity or Herbal medicine or *Plasmodium*.

**Fig. 1:**
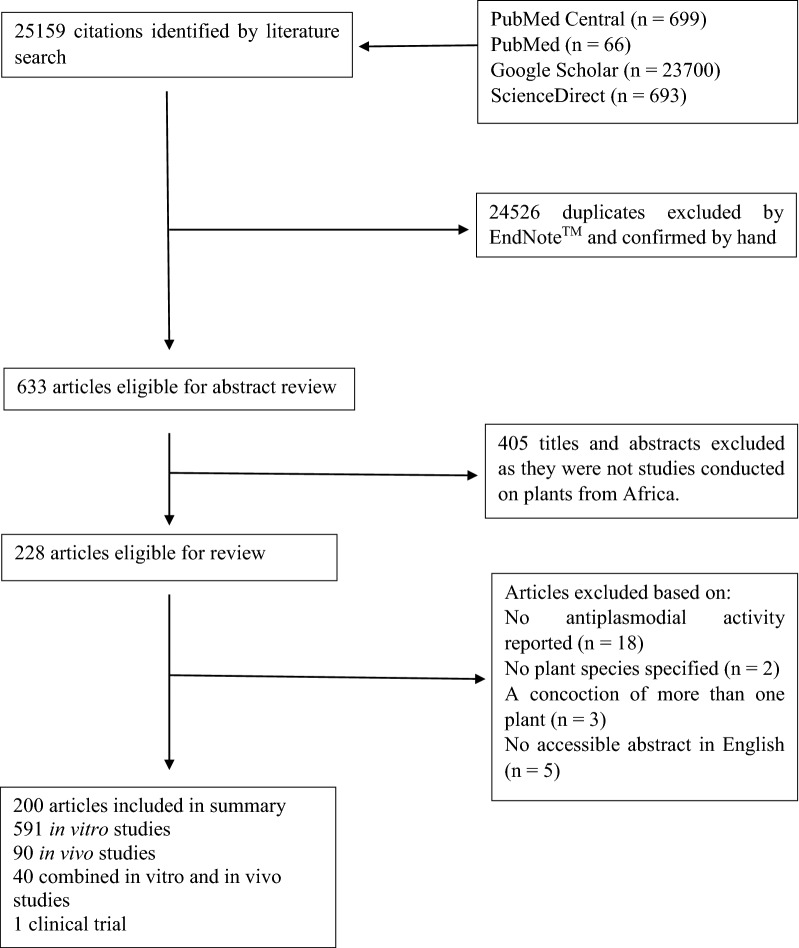
Flowchart of the selection process for publications included in this review

The search was limited to studies published in English or containing at least an abstract written in English until May 2020. The titles and abstracts were subsequently examined by two reviewers, independently (parallel method) to identify articles reporting the antiplasmodial activity of medicinal plants. In the case of any discrepancy in their reports, a third reviewer was brought in to resolve the issue. Relevant papers were equally manually cross-checked to identify further references. The following data were extracted from the selected articles by the reviewers: plant species, plant family, place of collection of plant, parts of the plant used, type of study (whether in *vitro*, in vivo, or human), the extraction solvent used, IC_50_ or ED_50_ values, parasite suppression rate, isolated compounds, interaction with known malarial drugs (whether synergistic or antagonistic), and toxicity. Articles that did not report antiplasmodial or anti-malarial activity of medicinal plants as well as review articles were excluded. The entire selection process is presented in Fig. 1.

In this study, antiplasmodial activity pertains to studies performed in vitro using different strains of *Plasmodium falciparum*, meanwhile, anti-malarial activity is reserved for in vivo studies performed using mice and various parasite models (including *Plasmodium berghei, Plasmodium yoelii,* and *Plasmodium chabaudi*) and reporting parasite suppression rate.

### Categorization of antiplasmodial and anti-malarial activities

For in vitro studies, the antiplasmodial activity of an extract was considered very good if IC_50_ < 5 µg/ml, good 5 µg/ml ≤ IC_50_ < 10 µg/ml, and moderate 10 µg/ml ≤ IC_50_ < 20 µg/ml [18]. For in vivo studies, the anti-malarial activity of an extract is considered very good if the suppression is ≥ 50% at 100 mg/kg body weight/day, good if the suppression is ≥ 50% at 250 mg/kg body weight/day, and moderate if the suppression is ≥ 50% at 500 mg/kg body weight/day [18]. Antiplasmodial activities of 20 µg/ml and above for in vitro studies and anti-malarial ≥ 50% at > 500 mg/kg body weight/day for in vivo studies, were considered inactive.

### Risk of bias in individual studies

The level of risk of bias for the study was likely to be high mainly because of differences in the studies and the methods used to determine the antiplasmodial or anti-malarial activity. The stains of *Plasmodium* used to assess the antiplasmodial or anti-malarial activity of the medicinal plants equally varied between studies. Furthermore, the extraction solvent, as well as the extraction yield of the plants in the different studies, was not the same, which may have accounted for the variation in the antiplasmodial and anti-malarial activities for the same plants but in the different studies.

## Results

The PRISMA flowchart (Fig. 1) presents a four-phase study selection process in the present systematic review study. A total of 25,159 titles were identified in the initial search. After the title and abstract screening, 228 full-text articles were retrieved. Of these, a final 200 articles were identified for the review.

**Fig. 2:**
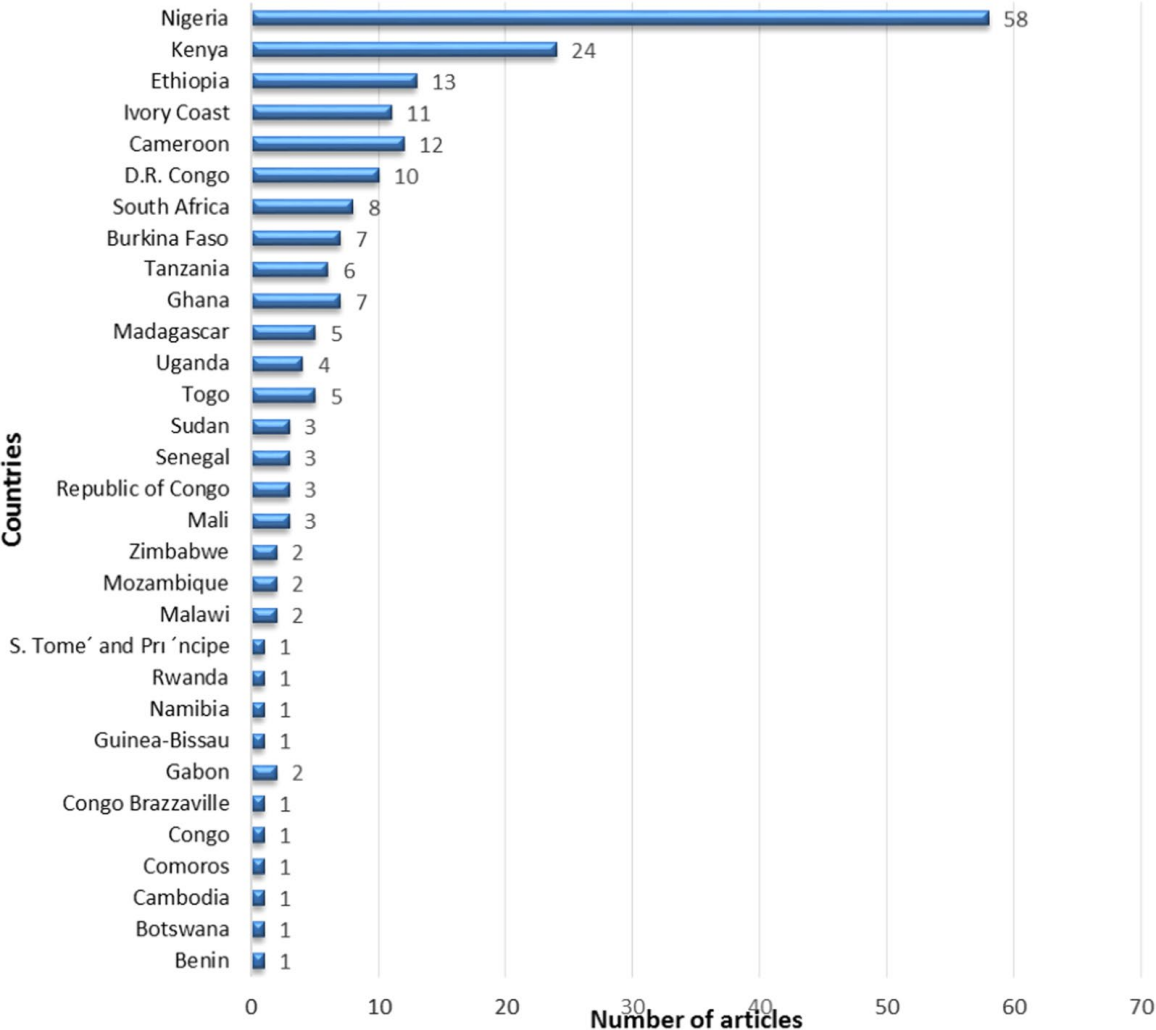
Distribution of the research articles on the antiplasmodial activity of indigenous plants according to African countries

For this review, the evaluation of the individual plant species was considered as an independent study, so it is common for one article to have more than one study depending on the number of plant species evaluated. In all, there were 722 independent studies. Five hundred and ninety-on (81.9%) of the independent studies were in vitro (Table 1), 90 (12.4%) were in vivo (Table 2) and 40 (5.5%) were both in vitro and in vivo (Table 3). There was only one human study (clinical trial) conducted so far (Table 4). The selected research articles were from 31 African countries. Out of the 200 research articles reviewed, most of them were from Nigeria 58 (29.0%), Kenya 24 (12.0%), Ethiopia 13 (6.5%), Cameroon 12 (6.0%), Ivory Coast 11 (5.5%), D.R. Congo 10 (5.0%), and Burkina Faso 7 (3.5%) (Fig. 2). The studies cover the period from 1989 to 2020.

**Table 1:** In vitro antiplasmodial activity of African medicinal plants

Plant species	Plant family	Source	Country of study	Part of plant used	Extraction solvent	Antiplasmodial Activity	IC_50_ or ED_50_ or LD_50_	Strain of *Plasmodium* Tested	Toxicity (value; assay)
*Dicoma anomala subsp. Gerrardii*	*Compositae*	[19]	South Africa	Whole plant	Methanol, Water, Hexane, Dichloromethane	Very good^a^	1.865 µM IC_50_	*Plasmodium falciparum* 3D7, D10	Nd
*Abutilon grandiflorum*	*Malvaceae*	[20]	Tanzania	Roots	Ethyl Acetate	Moderate	10 µg/ml IC_50_	*Plasmodium falciparum* K1	Nd
*Acacia mellifera*	*Fabaceae*	[21]	Kenya	Inner Barks	Methanol	Very Good	4.48 µg/ml IC_50_	*Plasmodium falciparum* D6	No
*Acacia nilotica*	*Fabaceae*	[22]	South Africa	Twigs	Dichloromethane/Methanol	Moderate	13 µg/ml IC_50_	*Plasmodium falciparum* D10	Nd
		[23]	Sudan	Seeds	Methanol	Very Good	0.9–4.1 µg/ml IC_50_	*Plasmodium falciparum* 3D7, Dd2	No
*Acacia polyacantha*	*Fabaceae*	[20]	Tanzania	Root Barkss	Ethyl Acetate	Moderate	13 µg/ml IC_50_	*Plasmodium falciparum* K1	Nd
*Acacia tortilis*	*Fabaceae*	[24]	Kenya	Stem Barks	Methanol	Moderate	13.4 µg/ml IC_50_	*Plasmodium falciparum* D6, W2	Nd
		[22]	South Africa	Whole Plant	Dichloromethane/Methanol	Very Good	4.8 µg/ml IC_50_	*Plasmodium falciparum* D10	Nd
*Acacia xanthoploea*	*Fabaceae*	[25]	South Africa	Stem Barks	Acetone	Moderate	10.1 µg/ml IC_50_	*Plasmodium falciparum* UP1 (CQ-R)	Nd
		[24]	Kenya	Stem Barks	Methanol	Moderate	17.3 µg/ml IC_50_	*Plasmodium falciparum* D6, W2	Nd
*Acacia mellifera*	*Fabaceae*	[24]	Kenya	Stem Barks	Methanol	Moderate	12.3 µg/ml IC_50_	*Plasmodium falciparum* D6, W2	Nd
*Amorpha fruticosa*	*Euphorbiaceae*	[26]	Kenya	Leaves	Methanol	Moderate	13.8 µg/ml IC_50_	*Plasmodium falciparum* D6, W2	Nd
*Acampe pachyglossa*	*Orchidaceae*	[20]	Kenya	Leaves	Ethyl Acetate	Moderate	11 µg/ml IC_50_	*Plasmodium falciparum* K1	Nd
*Acanthospermum hispidum DC*	*Compositae*	[27]	Burkina Faso	Stems, Leaves	Crude Alkaloid	Good	4–10 µg/ml IC_50_	*Plasmodium falciparum* W2	Nd
		[28]	Ivory Coast	Stems and Leaves	Ethanol	Moderate	13.7 µg/ml IC_50_	*Plasmodium falciparum* Fcb1/Colombia Strain	Nd
		[29]	Republic of Congo	Leaves	Methanolic, Ethanol	Very Good	2.8 µg/ml IC_50_	*Plasmodium falciparum*	No
*Achyranthes aspera*	*Amaranthaceae*	[22]	South Africa	Whole plant	Dichloromethane/Methanol	Good	9.9 µg/ml IC_50_	*Plasmodium falciparum* D10	Nd
*Acmella caulirhiza*	*Compositae*	[30]	Kenya	Whole plant	Dichloromethane	Good	5.201–9.939 µg/ml IC_50_	*Plasmodium falciparum* W2, D6	Nd
*Acridocarpus chloropterus*	*Malpighiaceae*	[31]	Tanzania	Roots	Dichloromethane	Good	5.06 µg/ml IC_50_	*Plasmodium falciparum* K1	No
*Achyranthes aspera*	*Amaranthaceae*	[20]	Tanzania	Root barks	Ethyl Acetate	Very Good	3 µg/ml IC_50_	*Plasmodium falciparum* K1	Nd
*Adansonia digitata*	*Malvaceae*	[20]	Kenya	Stem barks	Ethyl Acetate	Good	8.2 µg/ml IC_50_	*Plasmodium falciparum* K1	Nd
*Adenia cissampeloides*	*Passifloraceae*	[32]	Ghana	Whole plant	Ethanol	Good	8.521 µg/ml IC_50_	*Plasmodium falciparum* 3D7	Nd
*Adhatoda latibracteata*	*Acanthaceae*	[33]	Gabon	Stems	Dichloromethane	Very Good	0.7–1.6 µg/ml IC_50_	*Plasmodium falciparum* Fcbm W2	No
*Aerva javanica*	*Amaranthaceae*	[34]	Sudan	Whole plant	Petroleum Ether/Chloroform	Very Good	< 5 µg/ml IC_50_	*Plasmodium falciparum*	Nd
*Aerva lanata*	*Amaranthaceae*	[20]	Tanzania	Whole plant	Ethyl Acetate	Good	8.6 µg/ml IC_50_	*Plasmodium falciparum* K1	Nd
*Aframomum giganteum*	*Zingiberaceae*	[33]	Gabon	Stems	Dichloromethane	Moderate	8.3–13.5 µg/ml IC_50_	*Plasmodium falciparum* Fcbm W2	No
*Agathosma apiculata*	*Rutaceae*	[22]	South Africa	Whole plant	Dichloromethane/Methanol	Good	5.2 µg/ml IC_50_	*Plasmodium falciparum* D10	Nd
*Ageratum conyzoides*	*Compositae*	[24]	Kenya	Whole plant	Methanol	Moderate	11.5–12.1 µg/ml IC_50_	*Plasmodium falciparum* D6, W2	Nd
		[30]	Kenya	Whole plant	Dichloromethane	Very Good	2.15–3.444 µg/ml IC_50_	*Plasmodium falciparum* W2, D6	Nd
*Ajuga remota*	*Lamiaceae*	[35]	Kenya	Ns	Ns	Good^a^	8.2 µM IC_50_	*Plasmodium falciparum* FCA 20/GHA	No
		[35]	Kenya	Aerial parts	Chloroform	Good	8.2 µg/ml IC_50_	*Plasmodium falciparum* FCA 20/GHA	No
*Alafia barteri*	*Apocynaceae*	[36]	Nigeria	Leaves	Water	Very Good	1.5 µg/ml IC_50_	*Plasmodium falciparum*	Nd
*Albizia coriaria*	*Fabaceae*	[30]	Kenya	Stem barks	Dichloromethane	Good	6.798–10.679 µg/ml IC_50_	*Plasmodium falciparum* W2, D6	Nd
		[24]	Kenya	Stem barks	Methanol	Moderate	15.2–16.8 µg/ml C_50_	*Plasmodium falciparum* D6, W2	Nd
*Albizia gummifera*	*Fabaceae*	[24]	Kenya	Stem barks	Methanol	Good	6.7 µg/ml IC_50_	*Plasmodium falciparum* D6, W2	Nd
		[20]	Tanzania	Stem barks	Ethyl Acetate	Moderate	15 µg/ml IC_50_	*Plasmodium falciparum* K1	Nd
*Albizia versicolor Welw.ex Oliv*	*Fabaceae*	[37]	South Africa	Roots	Dichloromethane	Very Good	2.12 µg/ml IC_50_	*Plasmodium falciparum* NF54	Nd
*Alchornea cordifolia*	*Euphorbiaceae*	[38]	Ivory Coast	Leaves	Ethanol	Very Good^a^	0.2–0.5 μM IC_50_	Plasmodium falciparum Fcm29 Cameroon And Nigerian Strain	No
		[39]	Ivory Coast	Stems, leaves	Water, Ethanol, Pentane	Very Good	2.43–4.56 µg/ml IC_50_	Plasmodium falciparum Fcm29, Fcb1, Plasmodium falciparum CQ-S (Nigerian)	No
		[40]	D.R.Congo	Leaves	Water	Very Good	4.84 µg/ml IC_50_	*Plasmodium falciparum* K1	No
*Alepidea amatymbica*	*Apiaceae*	[22]	South Africa	Whole plant	Dichloromethane/Methanol	Moderate	12.5 µg/ml IC_50_	*Plasmodium falciparum* D10	Nd
*Aloe marlothii*	*Xanthorrhoeaceae*	[22]	South Africa	Whole plant	Dichloromethane	Very Good	3.5 µg/ml IC_50_	*Plasmodium falciparum* D10	Nd
*Aloe ferox*	*Xanthorrhoeaceae*	[22]	South Africa	Whole plant	Dichloromethane/Methanol	Good	8 µg/ml IC_50_	*Plasmodium falciparum* D10	Nd
*Aloe maculata*	*Xanthorrhoeaceae*	[22]	South Africa	Whole plant	Dichloromethane/Methanol	Moderate	12.4 µg/ml IC_50_	*Plasmodium falciparum* D10	Nd
*Aloe pulcherrima*	*Xanthorrhoeaceae*	[41]	Ethiopia	Roots	N-Hexane, Chloroform, Acetone Ans Methanol	Moderate^a^	18.6 µg/ml IC_50_	*Plasmodiumfalciparum*	Nd
*Aloe secundiflora*	*Xanthorrhoeaceae*	[24]	Kenya	Leaves	Methanol	Moderate	15.4 µg/ml IC_50_	*Plasmodium falciparum* D6, W2	Nd
*Alstonia boonei*	*Apocynaceae*	[42]	Nigeria	Stem barks	Ethanol	Nd	nd	*Plasmodium beghei* NK-65	No
		[43]	Ivory Coast	Stem barks	Ethanol	Moderate	12.3 µg/ml IC_50_	*Plasmodium falciparum* FCB1	Nd
*Alstonia congensis*	*Apocynaceae*	[44]	D.R. Congo	Leaves, Root Barks, Stem Barks	Water, Methanol	Very Good	2—5 µg/ml IC_50_	*Plasmodium falciparum* K1	Nd
*Ampelocissus africana*	*Vitaceae*	[20]	Kenya	Whole plant	Ethyl Acetate	Good	9.0 µg/ml IC_50_	*Plasmodium falciparum* K1	Nd
*Andrographis peniculata*	*Acanthaceae*	[45]	Cambodia	Whole plant	Dichloromethane	Moderate	12.7 µg/ml IC_50_	*Plasmodium falciparum* W2	Nd
*Annickia kummeriae*	*Annonaceae*	[31]	Tanzania	Leaves	Methanol	Very Good	0.12 µg/ml IC_50_	*Plasmodium falciparum* K1	No
Anisopappus chinensis	*Compositae*	[46]	D.R. Congo	Whole plant	Methanolic and dichloromethane	Good	6.53 µg/ml IC_50_	*Plasmodium falciparum* (3D7, W2), *Plasmodium berghei berghei*	No
Annona reticulata	*Annonaceae*	[47]	Cameroon	Roots	Ethanol	Very good	1.90 µg/ml IC_50_	*Plasmodium falciparum* W2	No
*Annona muricata*	*Annonaceae*	[48]	Ivory Coast	Leaves	Pentane	Moderate	8–18 µg/ml IC_50_	*Plasmodium falciparum* FCM29, *Plasmodium falciparum* CQ-S (Nigerian)	Nd
		[49]	Cameroon	Leaves	Hexane	Very Good	2.03 µg/ml IC_50_	*Plasmodium falciparum* W2	Nd
		[47]	Cameroon	Stem barks	Ethanol	Very Good	1.45 µg/ml IC_50_	*Plasmodium falciparum* W2	No
*Anogeissus leiocarpus*	*Combretaceae*	[50]	Nigeria	Ns	Methanol, Water, Butanol, Ethyl Acetate	Moderate	10.94–13.77 µg/ml IC_50_	*Plasmodium falciparum* 3D7, K1	Yes (SI = 121; mouse [NBMH])
		[51]	Ivory Coast	Leaves	Methylene Chloride	Very Good	3.8 µg/ml IC_50_	*Plasmodium falciparum* K1	No
*Anonidium mannii*	*Annonaceae*	[49]	Cameroon	Twigs	Methanol	Very Good	2.04 µg/ml IC_50_	*Plasmodium falciparum* W2	Nd
*Ansellia africana*	*Orchidaceae*	[20]	Tanzania	Leaves	Ethyl Acetate	Moderate	10 µg/ml IC_50_	*Plasmodium falciparum* K1	Nd
*Anthocleista grandiflora Gilg*	*Gentianaceae*	[37]	South Africa	Stem barks	Dichloromethane	Good	8.69 µg/ml IC_50_	*Plasmodium falciparum* NF54	Nd
*Anthocleista nobilis*	*Gentianaceae*	[52]	Burkina Faso	Leaves	Dichloromethane	Moderate	10 µg/ml	*Plasmodium falciparum*	Nd
*Anthocleista vogelii*	*Gentianaceae*	[53]	Nigeria	Roots	Petroleum Ether	Good	9.50 µg/ml IC_50_	*Plasmodium falciparum* D10	Nd
*Arenga engleri*	*Arecaceae*	[25]	South Africa	Stem barks	Dichloromethane	Very Good	1.7 µg/ml IC_50_	*Plasmodium falciparum* UP1 (CQ-R)	Yes (ID_50_ = 35 µg/ml; Monkey kidney cells)
*Artabotrys monteiroae*	*Annonaceae*	[22]	South Africa	Twigs	Dichloromethane/Methanol	Good	8.7 µg/ml IC_50_	*Plasmodium falciparum* D10	Nd
*Artemisia afra*	*Asteraceae*	[54]	Zimbabwe	Leaves	Petrolether/Ethylacetate	Moderate	8.9–15.3 µg/ml IC_50_	*Plasmodium falciparum* Pow, Dd2	Nd
		[22]	South Africa	Leaves	Dichloromethane	Good	5 µg/ml IC_50_	*Plasmodium falciparum* D10	Nd
		[24]	Kenya	Leaves	Methanol	Good	3.9–9.1 µg/ml C_50_	*Plasmodium falciparum* D6, W2	Nd
*Artemisia annua L*	*Asteraceae*	[24]	Kenya	Leaves	Methanol	Good	4.7–5.5 µg/ml C_50_	*Plasmodium falciparum* D6, W2	Nd
*Artocarpus communis*	*Moraceae*	[55]	Cameroon	Stems, Leaves	Ethanol, Water, Dichloromethane, Methanol, Hexane	Very Good	0.67–8.20 µg/ml IC_50_	*Plasmodium falciparum* W2	Nd
*Asparagus virgatus*	*Asparagaceae*	[22]	South Africa	Whole plant	Dichloromethane/Methanol	Good	8 µg/ml IC_50_	*Plasmodium falciparum* D10	Nd
*Aspilia africana*	*Asteraceae*	[56]	Uganda	Shoots	Ethyl Acetate	Moderate	9.3–11.5 µg/ml IC_50_	*Plasmodium falciparum* D10, K1	Nd
*Aspilia pruliseta*	*Compositae*	[24]	Kenya	Root BARKS	Methanol	Good	6.8–9.7 µg/ml C_50_	*Plasmodium falciparum* D6, W2	Nd
*Asystasia gangetica*	*Acanthaceae*	[22]	South Africa	Twigs	Dichloromethane/Methanol	Moderate	16 µg/ml IC_50_	*Plasmodium falciparum* D10	Nd
*Azadirachta indica*	*Meliaceae*	[57]	Ivory Coast	Stems, leaves	Water	Very Good	2.35–6.8 µg/ml IC_50_	*Plasmodium falciparum* Fcb1 & F32	Nd
		[45]	Cambodia	Barks	Dichloromethane	Very Good	4.7 µg/ml IC_50_	*Plasmodium falciparum* W2	Nd
		[58]	Sudan	Leaves	Methanol	Very Good	1.7–5.8 µg/ml IC_50_	*Plasmodium falciparum* 3D7, Dd5	Nd
		[59]	Togo	Leaves	Ethanol	Very Good	2.48–2.5 µg/ml IC_50_	*Plasmodium falciparum* W2, D6	Nd
*Azanza garckeana*	*Malvaceae*	[60]	Malawi	Leaves	Dichloromethane	Moderate	11·79 µg/ml IC_50_	*Plasmodium falciparum,* Vl/S	Nd
*Balanites aegyptiaca*	*Zygophyllaceae*	[24]	Kenya	Root barks	Methanol	Good	8.9 µg/ml C_50_	*Plasmodium falciparum* D6, W2	Nd
		[21]	Kenya	Root barks	Methanol	Very good	3.49 µg/ml IC_50_	*Plasmodium falciparum* D6	No
*Balanites maughamii*	*Zygophyllaceae*	[25]	South Africa	Stem barks	Dichloromethane	Very good	1.94 µg/ml IC_50_	*Plasmodium falciparum* UP1 (CQ-R)	Nd
*Barringtonia racemosa*	*Lecythidaceae*	[22]	South Africa	Twigs	Dichloromethane/Methanol	Good	5.7 µg/ml IC_50_	*Plasmodium falciparum* D10	Nd
*Berberis holstii*	*Berberidaceae*	[61]	Malawi	Roots	Dichloromethane/Methanol	Very good	0.17 µg/ml IC_50_	*Plasmodium falciparum* 3D7	Nd
		[24]	Kenya	Root barks	Methanol	Very Good	< 5 µg/ml C_50_	*Plasmodium falciparum* D6, W2	Nd
*Bergia suffruticosa*	*Elatinaceae*	[62]	Burkina Faso	Whole plant	Dichloromethane	Moderate	19.53 µg/ml IC_50_	*Plasmodium falciparum* 3D7 & W2	Nd
*Berula erecta*	*Apiaceae*	[22]	South Africa	Whole plant	Dichloromethane/Methanol	Good	6.6 µg/ml IC_50_	*Plasmodium falciparum* D10	Nd
		[24]	Kenya	Leaves	Methanol	Good	9.9 µg/ml C_50_	*Plasmodium falciparum* D6, W2	Nd
		[22]	South Africa	Leaves	Methanol	Good	5 µg/ml IC_50_	*Plasmodium falciparum* D10	Nd
Bidens engleri	*Compositae*	[63]	Senegal	Leaves	Petroleum ether	Moderate	9–18 µg/ml IC_50_	*Plasmodium falciparum* FcM29, FcB1, *Plasmodium vinckei petteri*	Yes (IC_50_ = 10 µg/ml; Vero cells)
*Bixa orellana*	*Bixaceae*	[45]	Cambodia	Leaves	Water	Good	9.3 µg/ml IC_50_	*Plasmodium falciparum* W2	Nd
*Boscia angustifolia*	*Capparaceae*	[24]	Kenya	Stem barks	Water	Very good	1.4–4.7 µg/ml C_50_	*Plasmodium falciparum* D6, W2	Nd
*Boscia salicifolia*	*Capparaceae*	[26]	Kenya	Stem barks	Methanol	good	1.1–8.8 µg/ml IC_50_	*Plasmodium falciparum* D6, W2	Nd
*Boswellia dalzielii*	*Burseraceae*	[50]	Nigeria	Ns	Methanol, Water, Butanol, Ethyl Acetate	Moderate	14.59–15.1 µg/ml IC_50_	*Plasmodium falciparum* 3D7, K1	Yes (SI ≥ 101; Mouse [NBMH]
		[62]	Burkina Faso	Leaves	Methanol	Moderate	18.85 µg/ml IC_50_	*Plasmodium falciparum* 3D7 & W2	Nd
*Bridelia micrantha*	*Phyllanthaceae*	[26]	Kenya	Stem Barks	Methanol	Moderate	14.2–19.4 µg/ml IC_50_	*Plasmodium falciparum* D6, W2	Nd
*Bridelia mollis Hutch*	*Phyllanthaceae*	[37]	South Africa	Roots	Dichloromethane	Very good	3.06 µg/ml IC_50_	*Plasmodium falciparum*NF54	Nd
*Brucea javanica*	*Simaroubaceae*	[45]	Cambodia	Roots	Dichloromethane	Very good	1.0 µg/ml IC_50_	*Plasmodium falciparum* W2	Nd
*Bruguiera gymnorhiza*	*Rhizophoraceae*	[22]	South Africa	Twigs	Dichloromethane/Methanol	Moderate	11.7 µg/ml IC_50_	*Plasmodium falciparum* D10	Nd
*Burchellia bubalina*	*Rubiaceae*	[22]	South Africa	Twigs	Dichloromethane/Methanol	Moderate	18 µg/ml IC_50_	*Plasmodium falciparum* D10	Nd
*Caesalpinia bonducella*	*Fabaceae*	[64]	Nigeria	Aerial Parts	Ethyl Acetate	Moderate	16 µg/ml EC_50_	*Plasmodium falciparum*	Yes (SI = 0.29–0.69; mouse mammary tumour [FM3A])
*Canthium setosum*	*Rubiaceae*	[65]	Benin	Aerial Parts	Methylene Chloride	Very good	2.77–4.80 µg/ml IC_50_	*Plasmodium falciparum* 3D7 & K1	Nd
*Capparis tomentosa Lam*	*Capparaceae*	[37]	South Africa	Roots	Dichloromethane	Very good	2.19 µg/ml IC_50_	*Plasmodium falciparum* NF54	Nd
*Cardiospermum halicacabum*	*Sapindaceae*	[22]	South Africa	Whole Plant	Dichloromethane/Methanol	Moderate	20 µg/ml IC_50_	*Plasmodium falciparum* D10	Nd
*Carica papaya*	*Caricaceae*	[66]	Nigeria	Leaves	Ethyl Acetate	Very good	2.96 µg/ml IC_50_	*Plasmodium falciparum* D10, DD2	No
*Carissa edulis*	*Apocynaceae*	[21]	Kenya	Root barks	Methanol	Good	6.41 µg/ml IC_50_	*Plasmodium falciparum* D6	No
*Carpolobia alba*	*Polygalaceae*	[53]	Nigeria	Roots	Dichloromethane	Good	7.10 µg/ml IC_50_	*Plasmodium falciparum* D10	Nd
*Cassia abbreviata*	*Fabaceae*	[60]	Malawi	Roots	Dichloromethane	Very Good	2·88 µg/ml IC_50_	*Plasmodium falciparum* Vl/S	Nd
*Cassia alata*	*Fabaceae*	[67]	D.R.Congo	Leaves	Ethanol, Methanol, Petroleum Ether, Chloroform	Very Good	< 0.1—5.4 µg/ml IC_50_	*Plasmodium Falciparum*	Nd
*Senna occidentalis L*	*Fabaceae*	[68]	Mozambique And Portugal	Roots	N-Hexane	Moderate	19.3 µg/ml IC_50_	*Plasmodium falciparum* 3D7	Nd
		[26]	Kenya	Root Barks	Methanol	Moderate	18.8 µg/ml IC_50_	*Plasmodium falciparum* D6, W2	Nd
		[69]	D.R. Congo	Leaves	Petroleum Ether	Very Good	1.5 µg/ml IC_50_	*Plasmodium falciparum*	Nd
		[67]	D.R. Congo	Leaves	Ethanol, Methanol, Petroleum Ether, Chloroform	Very Good	< 0.1—0.25 µg/ml IC_50_	*Plasmodium falciparum*	Nd
*Cassia siamea*	*Fabaceae*	[70]	Togo	Leaves	Water	Good	< 7 µg/ml IC_50_	*Plasmodium falciparum*	Nd
		[27]	Burkina Faso	Leaves	Crude Alkaloid	Good	4–10 µg/ml IC_50_	*Plasmodium falciparum* W2	Nd
*Cassia tora*	*Fabaceae*	[23]	Sudan	Aerial parts	Methanol	Good	3.3–5.2 µg/ml IC_50_	*Plasmodium falciparum* 3D7, Dd2	No
*Catha edulis*	*Celastraceae*	[22]	South Africa	Roots	Dichloromethane	Very Good	0.68 µg/ml IC_50_	*Plasmodium falciparum* D10	Nd
*Cedrelopsis grevei*	*Rutaceae*	[71]	Madagascar	Leaves	Water	Moderate	17.5 mg/L IC_50_	*Plasmodium falciparum*	Nd
*Celtis integrifolia*	*Cannabaceae*	[52]	Burkina Faso	Leaves	Dichloromethane	Very Good	3.7 µg/ml IC_50_	*Plasmodiumfalciparum*	Yes (SI ≥ 0.5; HepG2 cells)
*Centella asiatica*	*Apiaceae*	[22]	South Africa	Leaves	Dichloromethane/Methanol	Good	8.3 µg/ml IC_50_	*Plasmodium falciparum* D10	Nd
		[72]	Kenya	Root Barks	Dichloromethane	Moderate	14.9–15.4 µg/ml IC_50_	*Plasmodium falciparum* K1, NF54	Nd
*Cephalanthus natalensis*	*Rubiaceae*	[22]	South Africa	Twigs	Dichloromethane/Methanol	Moderate	16.5 µg/ml IC_50_	*Plasmodium falciparum* D10	Nd
Ceratotheca sesamoides	*Pedaliaceae*	[63]	Senegal	Leaves	Petroleum ether	Moderate	15–23 µg/ml IC_50_	*Plasmodium falciparum* FcM29, FcB1, *Plasmodium vinckei petteri*	Yes (IC_50_ = 50 µg/ml; Vero cells)
*Chrysophyllum perpulchrum*	*Sapotaceae*	[43]	Ivory Coast	Stem Barks	Ethanol	Moderate	12.8 µg/ml IC_50_	*Plasmodium falciparum*FCB1	Nd
*Cinchona succirubra*	*Rubiaceae*	[73]	S. Tome´ And Prı ´Ncipe	Barks	Petroleum Ether, Dichloromethane, Ethyl Acetate, Methanol	Good	< 10 µg/ml IC_50_	*Plasmodium falciparum*3D7 And Dd2	Nd
*Cinnamonum camphora*	*Lauraceae*	[57]	Ivory Coast	Cortex	Water	Moderate	9.37–16.6 µg/ml IC_50_	*Plasmodium falciparum*Fcb1 & F32	Nd
*Cissampelos mucronata*	*Menispermaceae*	[20]	Tanzania	Roots	Ethyl Acetate	Very Good	0.38 µg/ml IC50	*Plasmodium falciparum*K1	Nd
		[26]	Kenya	Leaves	Methanol	Very Good	4.4 µg/ml IC_50_	*Plasmodium falciparum*D6, W2	Nd
*Cissampelos pareira*	*Menispermaceae*	[24]	Kenya	Root Barks	Methanol	Good	5.2–6.5 µg/ml C_50_	*Plasmodium falciparum*D6, W2	Nd
		[74]	Kenya	Root	Methanol	Good	5.85–7.70 µg/ml IC_50_	*Plasmodium falciparum*NF54, ENT30	Nd
*Cissus populnea*	*Vitaceae*	[50]	Nigeria	Ns	Methanol, Water, Butanol, Ethyl Acetate	Moderate	15.81–19.91 µg/ml IC_50_	*Plasmodium falciparum*3D7, K1	Yes (SI ≥ 84, Mouse [NBMH])
*Citropsis articulata*	*Rutaceae*	[75]	Uganda	Root Barks	Ethyl Acetate	Nd	nd	*Plasmodium falciparum*Fcb1	Nd
*Clausena anisota*	*Rutaceae*	[24]	Kenya	Stem Barks	Methanol	Good	8.4–9.2 µg/ml C_50_	*Plasmodium falciparum*D6, W2	Nd
		[22]	South Africa	Twigs	Dichloromethane/Methanol	Moderate	18 µg/ml IC_50_	*Plasmodium falciparum*D10	Nd
*Clematis brachiata Thunb*	*Ranunculaceae*	[37]	South Africa	Roots	Dichloromethane	Good	5.36 µg/ml IC_50_	*Plasmodium falciparum*NF54	Nd
		[21]	Kenya	Root Barks	Methanol	Very Good	4.15 µg/ml IC_50_	*Plasmodium falciparum*D6	No
*Clerodendrum eriophyllum*	*Lamiaceae*	[72]	Kenya	Root Barks	Dichloromethane	Very Good	2.7–5.3 µg/ml IC_50_	*Plasmodium falciparum*K1, NF54	Nd
		[24]	Kenya	Leaves	Methanol	Very Good	< 1.8–3.9 µg/ml C_50_	*Plasmodium falciparum*D6, W2	Nd
*Clerodendrum glabrum E. Mey*	*Lamiaceae*	[37]	South Africa	Leaves	Dicloromethane	Good	8.89 µg/ml IC_50_	*Plasmodium falciparum*NF54	Nd
*Clerodendrum glabrum var. glabrum*	*Lamiaceae*	[22]	South Africa	Twigs	Dichloromethane/Methanol	Moderate	19 µg/ml IC_50_	*Plasmodium falciparum*D10	Nd
*Clerodendrum johnstonii*	*Lamiaceae*	[24]	Kenya	Root Barks	Methanol	Good	8.5 µg/ml C_50_	*Plasmodium falciparum*D6, W2	Nd
*Rotheca myricoides*	*Lamiaceae*	[76]	Kenya	Root Barks	Methanol	Good	4.0—8.4 µg/ml IC_50_	*Plasmodium falciparum*(K39, ENT30, NF54, V1/S)	Nd
		[26]	Kenya	Root Barks	Methanol	Good	4.7–8.3 µg/ml IC_50_	*Plasmodium falciparum*D6, W2	Nd
		[20]	Tanzania	Root Barks	Ethyl Acetate	Moderate	11 µg/ml IC_50_	*Plasmodium falciparum*K1	Nd
		[72]	Kenya	Root Barks	Dichloromethane	Moderate	10.9–15.8 µg/ml IC_50_	*Plasmodium falciparum*K1, NF54	Nd
*Clerodendrum rotundifolium*	*Lamiaceae*	[24]	Kenya	Leaves	Dichloromethane	Good	< 3.9–15.7 µg/ml C_50_	*Plasmodium falciparum*D6, W2	Nd
		[77]	Uganda	Leaves	Ethyl Acetate	Very Good	0.03–0.21 µg/ml IC_50_	*Plasmodium falciparum*NF54 & FCR3	Nd
*Clutia abyssinica*	*Peraceae*	[24]	Kenya	Leaves	Methanol	Moderate	7.8–11.3 µg/ml IC_50_	*Plasmodium falciparum* D6, W2	Nd
*Clutia hirsuta*	*Peraceae*	[22]	South Africa	Whole Plant	Dichloromethane/Methanol	Moderate	15 µg/ml IC_50_	*Plasmodium falciparum* D10	Nd
*Clutia robusta*	*Peraceae*	[24]	Kenya	Leaves	Methanol	Good	3.4–7.5 µg/ml IC_50_	*Plasmodium falciparum* D6, W2	Nd
*Cochlospermum planchonii*	*Bixaceae*	[78]	Burkina Faso	Rhizomes	Methanol, Dichloromethane	Good^a^	2.4–11.5 μg/ml IC_50_	*Plasmodium falciparum* 3D7	Nd
		[51]	Ivory Coast	Roots	Methylene Chloride	Very Good	4.4 µg/ml IC_50_	*Plasmodium falciparum* K1	No
*Cochlospermum tinctorium*	*Bixaceae*	[79]	Burkina Faso	Tubecles	Ns	Very Good	1–2 µg/ml IC_50_	*Plasmodium falciparum*	Nd
		[79]	Burkina Faso	Tubercles	Water	Very Good	0.4–1.56 µg/ml IC_50_	*Plasmodium falciparum* Fcbl And F32	Nd
*Cola caricaefolia*	*Malvaceae*	[48]	Ivory Coast	Leaves	Pentane	Moderate	11–16 µg/ml IC_50_	*Plasmodium falciparum* FCM29, CQ-S (Nigerian)	No
*Combretum collinum*	*Combretaceae*	[52]	Burkina Faso	Leaves	Dichloromethane	Very Good	0.2 µg/ml IC_50_	*Plasmodiumfalciparum*	Nd
*Combretum micranthum*	*Combretaceae*	[57]	Ivory Coast	Stem, Leaves	Water	Very Good	0.88–1.7 µg/ml IC_50_	*Plasmodium falciparum* Fcb1 & F32	Nd
*Combretum psidioides subsp. Psilophyllum*	*Combretaceae*	[20]	Tanzania	Root Barks	Ethyl Acetate	Good	6.5 µg/ml IC_50_	*Plasmodium falciparum* K1	Nd
*Combretum zeyheri*	*Combretaceae*	[22]	South Africa	Twigs	Dichloromethane/Methanol	Moderate	15 µg/ml IC_50_	*Plasmodium falciparum* D10	Nd
*Commiphora africana*	*Burseraceae*	[24]	Kenya	Stem Barks	Methanol	Good	9.6–10.2 µg/ml IC_50_	*Plasmodium falciparum* D6, W2	Nd
*Commiphora schimperi*	*Burseraceae*	[26]	Kenya	Stem Barks	Methanol	Very Good	3.9–5.2 µg/ml IC_50_	*Plasmodium falciparum* D6, W2	Nd
		[21]	Kenya	Inner Barks	Methanol	Very Good	4.63 µg/ml IC_50_	*Plasmodium falciparum* D6	No
*Conyza albida*	*Asteraceae*	[22]	South Africa	Whole Plant	Dichloromethane/Methanol	Very Good	2 µg/ml IC_50_	*Plasmodium falciparum* D10	Nd
*Conyza podocephala*	*Asteraceae*	[22]	South Africa	Whole Plant	Dichloromethane/Methanol	Good	6.8 µg/ml IC_50_	*Plasmodium falciparum* D10	Nd
*Conyza scabrida*	*Asteraceae*	[22]	South Africa	Flower	Dichloromethane/Methanol	Good	7.8 µg/ml IC_50_	*Plasmodium falciparum* D10	Nd
*Copaifera religiosa*	*Fabaceae*	[33]	Gabon	Leaves	Dichloromethane	Moderate	8.5–13.4 µg/ml IC_50_	*Plasmodium falciparum* FCB, 3D7	Yes (CC_50_ = 4.87 µg/ml; human embryonic lung cells [MRC-5])
*Cordia myxa*	*Boraginaceae*	[52]	Burkina Faso	Leaves	Dichloromethane	Good	6.2 µg/ml IC_50_	*Plasmodiumfalciparum*	Yes (SI = 0.5–0.9; HrpG2 cells)
*Coula edulis*	*Olacaceae*	[80]	Cameroon	Stem Barks	Methanol	Good	5.79–13.8 µg/ml IC_50_	*Plasmodium falciparum* 3D7, DD2	No
*Crossopteryx febrifuga*	*Rubiaceae*	[27]	Burkina Faso	Leaves	Crude Alkaloid	Good	4–10 µg/ml IC_50_	*Plasmodium falciparum* W2	Nd
*Crotalaria burkeana*	*Fabaceae*	[22]	South Africa	Roots	Dichloromethane	Good	9.5 µg/ml IC_50_	*Plasmodium falciparum* D10	Nd
*Croton gratissimus var. subgratissimus*	*Euphorbiaceae*	[22]	South Africa	Leaves	Dichloromethane	Very Good	3.5 µg/ml IC_50_	*Plasmodium falciparum* D10	Nd
*Croton lobatus*	*Euphorbiaceae*	[65]	Benin	Roots	Methanol	Good	2.80–6.56 µg/ml IC_50_	*Plasmodium falciparum* 3D7 & K1	Nd
*Croton macrostachyus*	*Euphorbiaceae*	[30]	Kenya	Leaves, Stems	Dichloromethane	Very Good	2.72 µg/ml IC_50_	*Plasmodium falciparum* W2, D6	Nd
*Croton menghartii*	*Euphorbiaceae*	[22]	South Africa	Leaves	Dichloromethane/Methanol	Very Good	1.7 µg/ml IC_50_	*Plasmodium falciparum* D10	Nd
*Croton pseudopulchellus*	*Euphorbiaceae*	[25]	South Africa	Stem Barks	Chloroform	Very Good	3.45 µg/ml IC_50_	*Plasmodium falciparum* UP1 (CQ-R)	Nd
*Croton zambesicus*	*Euphorbiaceae*	[55]	Cameroon	Stem Barks	Ethanol, Water, Dichloromethane, Methanol, Hexane	Good	0.88–9.14 µg/ml IC_50_	*Plasmodium falciparum* W2	Nd
		[34]	Sudan	Fruits	Petroleum Ether/Chloroform	Very Good	< 5 µg/ml IC_50_	*Plasmodium falciparum*	Nd
*Cryptolepis sanguinolenta*	*Apocynaceae*	[81]	Guinea-Bissau	Leaves, Roots	Ethanol, Chcl3, Chloroform	Very Good	1.79 µg/ml IC_50_	*Plasmodium falciparum* K1, T996	Nd
		[82]	Ghana	Roots	Ethanol	Very good^a^	0.031 µg/ml IC_50_	*Plasmodium falciparum* K1, *Plasmodium berghei*	Nd
		[83]	D.R. Congo	Root barks	Water, ethanol, chloroform	Very good	27–41 ng/ml IC_50_	*Plasmodium falciparum* D6, K1, W2, *Plasmodium berghei yoelii, Plasmodium berghei berghei*	Nd
		[84]	Ghana	Roots	Hexane, ethanol, dichloromethane	Very good^a^	0.2–0.6 μM IC_50_	*Plasmodium vinckei petteri, Plasmodium berghei* ANKA	Nd
*Cussonia spicata Thunb*	*Araliaceae*	[22]	South Africa	Fruits	Dichloromethane/Methanol	Moderate	14 µg/ml IC_50_	*Plasmodium falciparum* D10	Nd
		[37]	South Africa	Root Barks	Dichloromethane	Very Good	3.25 µg/ml IC_50_	*Plasmodium falciparum* NF54	Nd
*Cussonia zimmermannii*	*Araliaceae*	[20]	Tanzania	Root Barks	Petroleum Ether	Very Good	3.3 µg/ml IC_50_	*Plasmodium falciparum* K1	Nd
*Cuviera longiflora*	*Rubiaceae*	[80]	Cameroon	Leaves	Dichloromethane/Methanol	Moderate	13.91–20.24 µg/ml IC_50_	*Plasmodium falciparum* 3D7, DD2	No
*Cyathala prostate*	*Amaranthaceae*	[43]	Ivory Coast	Whole Plant	Ethanol	Moderate	12.4 µg/ml IC_50_	*Plasmodium falciparum FCB1*	Nd
*Cyathula schimperiana*	*Amaranthaceae*	[24]	Kenya	Root Barks	Methanol	Moderate	5–17.6 µg/ml C_50_	*Plasmodium falciparum* D6, W2	Nd
*Cymbopogon validus*	*Poaceae*	[22]	South Africa	Whole Plant	Dichloromethane/Methanol	Good	5.8 µg/ml IC_50_	*Plasmodium falciparum* D10	Nd
*Cyperus articulatus*	*Cyperaceae*	[24]	Kenya	Tubers	Methanol	Good	4.8–8.7 µg/ml C_50_	*Plasmodium falciparum* D6, W2	Nd
		[74]	Kenya	Rhizomes	Methanol	Good	4.84–8.68 µg/ml IC_50_	*Plasmodium falciparum* NF54, ENT30	Nd
*Cyphostemma spp*	*Vitaceae*	[86]	Namibia	Whole Plant	Methanol	Very Good	3.276 µg/ml IC_50_	*Plasmodium falciparum* 3D7	Nd
*Dacryodes edulis*	*Burseraceae*	[80]	Cameroon	Leaves	Dichloromethane/Methanol	Good	6.45–8.62 µg/ml IC_50_	*Plasmodium falciparum* 3D7, DD2	No
		[85]	Cameroon	Root Barks	Methylene Chloride/Methanol	Very Good	0.37 µg/ml IC_50_	*Plasmodiumfalciparum*	No
*Dichapetalum guineense*	*Dichapetalaceae*	[65]	Benin	Leaves	Methanol	Moderate	7.35- > 20 µg/ml IC_50_	*Plasmodium falciparum* 3D7 & K1	Nd
*Dichrostachys cinerea Wight et Arn*	*Fabaceae*	[37]	South Africa	Roots	Dichloromethane	Very Good	2.1 µg/ml IC_50_	*Plasmodium falciparum* NF54	Nd
*Dicoma tomentosa*	*Asteraceae*	[62]	Burkina Faso	Whole Plant	Dichloromethane, Methanol	Good	7.04–7.90 µg/ml IC_50_	*Plasmodium falciparum* 3D7 & W2	Nd
		[87]	Burkina Faso	Whole plant	Dichloromethane	Very Good	1.9–3.4 µg/ml IC_50_	*Plasmodium Falcipârum* 3D7, W2, *Plasmodium berghei*	Nd
*Diospyros abysinica*	*Ebenaceae*	[75]	Uganda	Leaves	Ethyl Acetate	Nd	nd	*Plasmodium falciparum* Fcb2	Nd
*Diospyros mespiliformis*	*Ebeneceae*	[86]	Namibia	Leaves, Roots	Methanol	Very Good	3.179–3.523 µg/ml IC_50_	*Plasmodium falciparum* 3D7	Nd
		[37]	South Africa	Roots	Dichloromethane	Very Good	4.40 µg/ml IC_50_	*Plasmodium falciparum* NF54	Nd
*Diospyros monbuttensis*	*Ebenaceae*	[88]	Nigeria	Leaves	Methanol	Very Good	3.2 nM	*Plasmodium falciparum*	Nd
*Dombeya shupangae*	*Malvaceae*	[20]	Tanzania	Root Barks	Ethyl Acetate	Good	7.5 µg/ml IC_50_	*Plasmodium falciparum* K1	Nd
*Dorstenia convexa*	*Moraceae*	[56]	Cameroon	Twigs	Ethanol, Water, Dichloromethane, Methanol, Hexane	Good	0.28–8.95 µg/ml IC_50_	*Plasmodium falciparum* W2	Nd
*Dorstenia klaineana*	*Moraceae*	[33]	Gabon	Stems	Methanol	Moderate	16.7–17.0 µg/ml IC_50_	*Plasmodium falciparum* Fcbm, W2	Yes (SI = 16.2–28.89; human embryonic lung cells [MRC-5])
*Dracaena cambodiana*	*Asparagaceae*	[45]	Cambodia	Stems	Dichloromethane	Good	8.7 µg/ml IC_50_	*Plasmodium falciparum* W2	Nd
*Drypetes natalensis*	*Putranjivaceae*	[31]	Tanzania	Roots	Ethanol	Very Good	1.06 µg/ml IC_50_	*Plasmodium falciparum* K1	No
*Ekebergia capensis*	*Meliaceae*	[22]	South Africa	Fruits	Dichloromethane/Methanol	Moderate	10 µg/ml IC_50_	*Plasmodium falciparum* D10	Nd
		[76]	Kenya	Stem Barks	Chloroform	Good	3.9—13.4 µg/ml IC_50_	*Plasmodium falciparum* K39, ENT30, NF54, V1/S	Nd
		[21]	Kenya	Inner Barks	Methanol	Very Good	3.97 µg/ml IC_50_	*Plasmodium falciparum* D6	No
		[24]	Kenya	Stem Barks	Methanol	Moderate	10.5 µg/ml IC_50_	*Plasmodium falciparum* D6, W2	Nd
*Elaeis guineensis*	*Arecaceae*	[32]	Ghana	Leaves	Ethanol	Very Good	1.195 µg/ml IC_50_	*Plasmodium falciparum* 3D7	Nd
*Elaeodendron buchananii*	*Celastraceae*	[24]	Kenya	Stem Barks	Methanol	Moderate	17.1 µg/ml IC_50_	*Plasmodium falciparum* D6, W2	Nd
*Enantia chlorantha*	*Annonaceae*	[55]	Cameroon	Stem Barks	Ethanol, Water, Dichloromethane, Methanol, Hexane	Good	0.68–14.72 µg/ml IC_50_	*Plasmodium falciparum* W2	Nd
		[40]	DR Congo	Stem Barks	Water	Good	7.77 µg/ml IC_50_	*Plasmodium falciparum* K1	Yes (CC_50_ = 3.0 µg/ml; human embryonic lung cells [MRC-5])
*Entandrophragma angolense*	*Meliaceae*	[89]	Cameroon	Stem Barks	Dichloromethane/Methanol	Moderate	18.4 µg/ml IC_50_	*Plasmodium falciparum* W2	Nd
*Entandrophragma caudatum*	*Meliaceae*	[25]	South Africa	Stem Barks	Dichloromethane	Very Good	2.9 µg/ml IC_50_	*Plasmodium falciparum* UP1 (CQ-R)	No
Entandrophragma palustre	*Meliaceae*	[46]	D.R. Congo	Stem barks	Methanol	Moderate	15.84 µg/ml IC_50_	*Plasmodium falciparum* 3D7, W2, *Plasmodium berghei berghei*	Nd
*Erigeron floribundus*	*Asteraceae*	[48]	Ivory Coast	Leaves	Pentane	Good	4.3-10 µg/ml IC_50_	*Plasmodium falciparum* FCM29, *Plasmodium falciparum* CQ-S (Nigerian)	Nd
*Erioglossum edule*	*Sapindaceae*	[45]	Cambodia	Barks	Dichloromethane	Very Good	1.7 µg/ml IC_50_	*Plasmodium falciparum* W2	Nd
*Erythrina abyssinica*	*Fabaceae*	[75]	Uganda	Barks	Ethyl Acetate	Nd	nd	*Plasmodium falciparum* Fcb3	Nd
*Erythrina lysistemon*	*Fabaceae*	[25]	South Africa	Stem Barks	Acetone	Very Good	4.8 µg/ml IC_50_	*Plasmodium falciparum* UP1 (CQ-R)	Nd
*Erythrina sacleuxii*	*Fabaceae*	[20]	Tanzania	Root Barks	Ethyl Acetate	Very Good	3.0 µg/ml IC_50_	*Plasmodium falciparum* K1	Nd
*Erythrococca anomala*	*Euphorbiaceae*	[43]	Ivory Coast	Leaves	Ethanol	Moderate	13.1 µg/dl IC_50_	*Plasmodium falciparum* FCB1	Nd
*Euclea divinorum*	*Ebenaceae*	[24]	Kenya	Root Barks	Methanol	Good	6.9–12.4 µg/ml IC_50_	*Plasmodium falciparum* D6, W2	Nd
*Euclea natalensis*	*Ebenaceae*	[22]	South Africa	Twigs	Dichloromethane/Methanol	Very Good	4.6 µg/ml IC_50_	*Plasmodium falciparum* D10	Nd
*Eucomis autumnalis*	*Asparagaceae*	[22]	South Africa	Bulbs	Dichloromethane/Methanol	Good	9.5 µg/ml IC_50_	*Plasmodium falciparum* D10	Nd
*Euphorbia hirta*	*Euphorbiaceae*	[90]	D.R. Congo	Aerial Parts	Methanol, Hexane: Ethyl Acetate	Good^a^	1.1—5.4 µg/ml IC_50_	*Plasmodium falciparum*	No
		[70]	D.R. Congo	Whole Plant	Petroleum Ether	Very Good	1.2 µg/ml IC_50_	*Plasmodium falciparum*	Nd
*Euphorbia tirucalli*	*Euphorbiaceae*	[22]	South Africa	Leaves	Dichloromethane	Moderate	12 µg/ml IC_50_	*Plasmodium falciparum* D10	Nd
*Fadogia agrestis*	*Rubiaceae*	[27]	Burkina Faso	Leaves	Crude Alkaloid	Good	4–10 µg/ml IC_50_	*Plasmodium falciparum* W2	Nd
*Fagara macrophylla*	*Rutaceae*	[28]	Ivory Coast	Stem Barks	Ethanol	Very Good	2.3 µg/ml IC_50_	*Plasmodium falciparum* Fcb1/Colombia Strain	No
*Fagaropsis angolensis*	*Rutaceae*	[24]	Kenya	Stem Barks	Methanol	Good	4.2–6.9 µg/ml IC_50_	*Plasmodium falciparum* D6, W2	Nd
*Fagraea fragrans*	*Gentianaceae*	[45]	Cambodia	Stems	Dichloromethane	Moderate	12.8 µg/ml IC_50_	*Plasmodium falciparum* W2	Nd
*Ficus capraefolia*	*Moraceae*	[52]	Burkina Faso	Leaves	Dichloromethane	Very Good	1.8 µg/ml IC_50_	*Plasmodium falciparum*	Yes (SI = 0.4; HepG2 cells)
*Ficus platyhylla*	*Moraceae*	[50]	Nigeria	Ns	Methanol, Water, Butanol, Ethyl Acetate	Moderate	13.77–15.28 µg/ml IC_50_	*Plasmodium falciparum* 3D7, K1	Yes (SI ≥ 77; mouse [NBMH])
*Ficus sur*	*Moraceae*	[24]	Kenya	Stem Barks	Methanol	Moderate	8.5–15.9 µg/ml IC_50_	*Plasmodium falciparum* D6, W2	Nd
		[76]	Kenya	Stem Barks	Chloroform, Hexane	Moderate	9.0–19.2 µg/ml IC_50_	*Plasmodium falciparum* K39 (CQ-S), ENT30, NF54, V1/S	Nd
*Ficus thonningii*	*Moraceae*	[29]	Republic Of Congo	Leaves	Methanol, Ethanol	Good	9.61 µg/ml IC_50_	*Plasmodium falciparum*	No
		[50]	Nigeria	Ns	Methanol, Water, Butanol, Ethyl Acetate	Moderate	14.09–25.06 µg/ml IC_50_	*Plasmodium falciparum* 3D7, K1	Yes (SI ≥ 103; mouse [NBMH])
*Ficus sycomorus*	*Moraceae*	[27]	Burkina Faso	Leaves	Crude Alkaloid	Good	4–10 µg/ml IC_50_	*Plasmodium falciparum* W2	Nd
*Flueggea virosa*	*Phyllanthaceae*	[91]	Comoros	Leaves	Water/Methanol	Very Good	2 µg/ml IC_50_	*Plasmodium falciparum* W2	No
		[26]	Kenya	Stem Barks	Methanol	Very Good	2.2–3.6 µg/ml IC_50_	*Plasmodium falciparum* D6, W2	Nd
		[22]	South Africa	Leaves, Twigs	Water	Moderate	11.4 µg/ml IC_50_	*Plasmodium falciparum* D10	Nd
*Fuerstia africana*	*Lamiaceae*	[92]	Rwanda	Leaves, Stems	Methanol	Good	4.1–6.9 µg/ml IC_50_	*Plasmodium falciparum* 3D7, W2	Yes (SI = 1.9; human normal foetal lung fibroblast [WI-38)
		[21]	Kenya	Leaves	Methanol	Very Good	3.76 µg/ml IC_50_	*Plasmodium falciparum* D6	No
		[24]	Kenya	Whole Plant	Methanol	Very Good	0.9–2.4 µg/ml IC_50_	*Plasmodium falciparum* D6, W2	Nd
*Funtumia elastica*	*Apocynaceae*	[43]	Ivory Coast	Stem Barks	Ethanol	Very Good	3.6 µg/ml IC_50_	*Plasmodium falciparum* FCB1	Nd
		[28]	Ivory Coast	Stem Barks	Ethanol	Very Good	3.3 µg/ml IC_50_	*Plasmodium falciparum* Fcb1/Colombia Strain	No
*Funtumia latifolia*	*Apocynaceae*	[75]	Uganda	Leaves	Ethyl Acetate	Nd	nd	*Plasmodium falciparum* Fcb4	Nd
*Garcinia kola*	*Clusiaceae*	[67]	D.R. Congo	Seeds	Ethanol, Methanol, Petroleum Ether, Chloroform	Good	1.02—15.75 µg/ml IC_50_	*Plasmodium falciparum*	Nd
		[69]	D.R. Congo	Stem Barks	Petroleum Ether	Very Good	1.6 µg/ml IC_50_	*Plasmodium falciparum*	Nd
*Gardenia lutea*	*Rubiaceae*	[23]	Sudan	Leaves	Methanol	Good	3.3–5.2 µg/ml IC_50_	*Plasmodium falciparum* 3D7, Dd2	No
*Gardenia sokotensis*	*Rubiaceae*	[62]	Burkina Faso	Leaves	Dichloromethane	Moderate	14.01 µg/ml IC_50_	*Plasmodium falciparum* 3D7 & W2	Nd
*Glinus oppositifolius*	*Molluginaceae*	[93]	Mali	Aerial parts	Chloroform	Moderate	15.52–18.70 µg/ml IC_50_	*Plasmodium falciparum* W2 & 3D7	No
*Gloriosa superba*	*Colchicaceae*	[22]	South Africa	Whole plant	Dichloromethane/Methanol	Moderate	17 µg/ml IC_50_	*Plasmodium falciparum* D10	Nd
*Gnidia cuneata*	*Thymelaeaceae*	[22]	South Africa	Stems	Dichloromethane	Moderate	15.9 µg/ml IC_50_	*Plasmodium falciparum* D10	Nd
*Gnidia kraussiana var. kraussiana*	*Thymelaeaceae*	[22]	South Africa	Leaves, Twigs	Dichloromethane/Methanol	Moderate	10.8 µg/ml IC_50_	*Plasmodium falciparum* D10	Nd
*Gomphrena celosioides*	*Amaranthaceae*	[65]	Benin	Aerial Parts	Methanol	Good	4.26–14.97 µg/ml IC_50_	*Plasmodium falciparum* 3D7 & K1	Nd
		[70]	Togo	Aerial Parts	Water	Moderate	< 15 µg/ml IC_50_	*Plasmodium falciparum*	Nd
		[20]	Tanzania	Whole plant	Ethyl Acetate	Moderate	15 µg/ml IC_50_	*Plasmodium falciparum* K1	Nd
*Guiera senegalensis*	*Combretaceae*	[57]	Ivory Coast	Stem, Leave	Water	Good	0.79–7.03 µg/ml IC_50_	*Plasmodium falciparum* Fcb1 & F32	Nd
		[94]	Mali	Roots	Chloroform	Very Good^a^	< 4 µg/ml IC_50_	*Plasmodium falciparum*	Nd
*Gutenbergia cordifolia*	*Asteraceae*	[21]	Kenya	Leaves	Methanol	Very Good	4.40 µg/ml IC_50_	*Plasmodium falciparum* D6	No
*Gynandropsis gynandra*	*Cleomaceae*	[20]	Tanzania	Roots	Ethyl Acetate	Moderate	14 µg/ml IC_50_	*Plasmodium falciparum* K1	Nd
*H. suaveolens*	*Lamiaceae*	[53]	Nigeria	Leaves	Petroleum Ether	Very Good	2.54 µg/ml IC_50_	*Plasmodium falciparum* D10	Nd
*Haplophyllum tuberculatum*	*Rutaceae*	[23]	Sudan	Aerial Parts	Methanol	Very Good	1.2–1.5 µg/ml IC_50_	*Plasmodium falciparum* 3D7, Dd2	No
*Harrisonia abyssinica*	*Rutaceae*	[58]	Sudan	Stem Barks	Methanol	Good	4.7–10 µg/ml IC_50_	*Plasmodium falciparum* 3D7, Dd3	Nd
		[72]	Kenya	Stem Barks	Dichloromethane	Good	4.4–5.6 µg/ml IC_50_	*Plasmodium falciparum* K1, NF54	Nd
		[26]	Kenya	Root Barks	Methanol	Good	7.8–11.4 µg/ml IC_50_	*Plasmodium falciparum* D6, W2	Nd
		[95]	Kenya	Barks/Roots/Stem	Water	Very Good	1.0 µg/ml IC_50_	*Plasmodium Knowlesi*	Nd
*Harrisonia perforata*	*Rutaceae*	[45]	Cambodia	StemS	Dichloromethane	Good	6.0 µg/ml IC_50_	*Plasmodium falciparum* W2	Nd
*Harungana madagascariensis*	*Hypericaceae*	[40]	D.R.Congo	Stem Barks	Water	Good	9.64 µg/ml IC_50_	*Plasmodium falciparum* K1	No
		[20]	Tanzania	Roots	Ethyl Acetate	Very Good	4.0 µg/ml IC_50_	*Plasmodium falciparum* K1	Nd
*Helichrysum gymnocephalum*	*Asteraceae*	[96]	Madagascar	Leaves	Essential Oil	In Active	25 mg/l	*Plasmodium falciparum* Fcb1	Nd
*Helichrysum cymosum*	*Asteraceae*	[97]	South Africa	Leaves	Water, Essential Oil	Very Good^a^	1.25 µg/ml IC_50_	*Plasmodium falciparum* FCR-3	Yes
*Helichrysum nudifolium*	*Asteraceae*	[22]	South Africa	Whole plant	Dichloromethane/Methanol	Good	6.8 µg/ml IC_50_	*Plasmodium falciparum* D10	Nd
*Hermannia depressa*	*Malvaceae*	[22]	South Africa	Whole plant	Dichloromethane/Methanol	Good	6.9 µg/ml IC_50_	*Plasmodium falciparum* D10	Nd
*Hexalobus crispiflorus*	*Annonaceae*	[98]	Cameroon	Stem Barks	Water	Very Good^a^	2.0 µg/ml IC_50_	*Plasmodium falciparum* W6	Nd
*Hippobromus pauciflorus*	*Sapindaceae*	[22]	South Africa	Twigs	Dichloromethane/Methanol	Good	5.9 µg/ml IC_50_	*Plasmodium falciparum D10*	Nd
*Holarrhena floribunda*	*Apocynaceae*	[99]	Cameroon	Stem Barkss	Water, Ethanol	Good	1.02 − 18.53 μg/mL IC_50_	*Plasmodium falciparum* W2,D6, FCR-3, 3D7	Nd
*Hoslundia opposita*	*Lamiaceae*	[20]	Tanzania	Root Barks	Petroleum Ether	Moderate	10 µg/ml IC_50_	*Plasmodium falciparum* K1	Nd
		[26]	Kenya	Leaves	Methanol	Moderate	15.2–25.6 µg/ml IC_50_	*Plasmodium falciparum* D6, W2	Nd
		[75]	Uganda	Leaves	Ethyl Acetate	Nd	nd	*Plasmodium falciparum* Fcb5	Nd
*Hunteria eburnea*	*Apocynaceae*	[43]	Ivory Coast	Stem Barks	Ethanol	Very Good	2.2 µg/ml IC_50_	*Plasmodium falciparum* FCB1	Nd
*Hybanthus enneaspermus*	*Violaceae*	[65]	Benin	Aerial Parts	Methanol	Moderate	2.57- > 20 µg/ml IC_50_	*Plasmodium falciparum* 3D7 & K1	Nd
*Hymenocardia acida*	*Phyllanthaceae*	[51]	Ivory Coast	Leaves	Methylene Chloride	Good	6.9 µg/ml IC_50_	*Plasmodium falciparum* K1	Yes (SI = 6–10; rat skeletal muscle myoblast [L6])
*Hypericum aethiopicum*	*Hypericaceae*	[22]	South Africa	Leaves/Flowers	Dichloromethane/Methanol	Very Good	1.4 µg/ml IC_50_	*Plasmodium falciparum* D10	Nd
*Hypericum lanceolatum*	*Hypericaceae*	[80]	Cameroon	Stem Barks	Methanol, N-Hexane, Ethyl Acetate, N-Butanol	Very Good	3.98 µg/ml IC_50_	*Plasmodium falciparum* W2, SHF4	Nd
*Hypoestes forskaolii*	*Acanthaceae*	[24]	Kenya	Root Barks	Methanol	Good	4.3–6.7 µg/ml IC_50_	*Plasmodium falciparum* D6, W2	Nd
*Hyptis pectinata*	*Lamiaceae*	[22]	South Africa	Leaves, Stem, Flower	Dichloromethane/Methanol	Moderate	17.5 µg/ml IC_50_	*Plasmodium falciparum* D10	Nd
*Icacina senegalensis*	*Icacinaceae*	[100]	Senegal	Leaves	Methanol	Good	4.7–8 µg/ml IC_50_	*Plasmodium falciparum* 3D7, 7G8	No
*Isolona hexaloba*	*Annonaceae*	[40]	D.R. Congo	Root Barks	Water	Moderate	15.28 µg/ml IC_50_	*Plasmodium falciparum* K1	No
*Khaya grandifoliola*	*Meliaceae*	[101]	Cameroon	Barks, Seeds	Methanol-Methylene Chloride	Good^a^	1.25—9.63 μg/ml IC_50_	*Plasmodium falciparum* W2	Nd
*Khaya senegalensis*	*Meliaceae*	[50]	Nigeria	Ns	Methanol, Water, Butanol, Ethyl Acetate	Moderate	15.46–28.12 µg/ml IC_50_	*Plasmodium falciparum* 3D7, K1	Yes (SI ≥ 69; mouse [NBMH])
*Kigelia africana*	*Bignoniaceae*	[24]	Kenya	Leaves	Methanol	Moderate	15.9 µg/ml IC_50_	*Plasmodium falciparum* D6, W2	Nd
		[80]	Cameroon	Stem Barks	Ethyl Acetate	Moderate	11.15 μg/mL IC_50_	*Plasmodium falciparum* W2	No
*Kirkia wilmsii*	*Kirkiaceae*	[22]	South Africa	Leaves	Dichloromethane/Methanol	Very Good	3.7 µg/ml IC_50_	*Plasmodium falciparum* D10	Nd
*Kniphofia foliosa*	*Xanthorrhoeaceae*	[102]	Ethiopia	Roots	Dichloromethane	Very Good	3.8 µg/mL ED_50_	*Plasmodium falciparum* 3D7	No
*Landolphia lanceolata*	*Apocynaceae*	[103]	Congo Brazzaville	Roots	Dichloromethane	Moderate	11 µg/ml IC_50_	*Plasmodium falciparum* Fcm29-Cameroon	Nd
*Lannea edulis*	*Anacardiaceae*	[20]	Kenya	Whole Plant	Ethyl Acetate	Moderate	17 µg/ml IC_50_	*Plasmodium falciparum* K1	Nd
*Lantana camara*	*Verbenaceae*	[22]	South Africa	Leaves, Twigs	Dichloromethane/Methanol	Moderate	11 µg/ml IC_50_	*Plasmodium falciparum* D10	Nd
*Leonotis mollissima*	*Lamiaceae*	[20]	Tanzania	Leaves	Ethyl Acetate	Good	9 µg/ml IC_50_	*Plasmodium falciparum* K1	Nd
*Leonotis africana*	*Lamiaceae*	[33]	Gabon	Stems	Dichloromethane	Moderate	15.2–27.1 µg/ml IC_50_	*Plasmodium falciparum* Fcbm W2	Yes (SI = 6.07–6.82; human embryonic lung cells [MRC-5])
*Leonotis leonurus*	*Lamiaceae*	[22]	South Africa	Leaves, Twigs	Dichloromethane/Methanol	Good	5.4 µg/ml IC_50_	*Plasmodium falciparum* D10	Nd
*Leonotis nepetifolia*	*Lamiaceae*	[22]	South Africa	Whole Plant	Dichloromethane/Methanol	Moderate	15 µg/ml IC_50_	*Plasmodium falciparum* D10	Nd
*Leonotis ocymifolia*	*Lamiaceae*	[22]	South Africa	Leaves	Dichloromethane/Methanol	Good	6.1 µg/ml IC_50_	*Plasmodium falciparum* D10	Nd
*Leptadenia madagascariensis*	*Apocynaceae*	[91]	Comoros	Ns	Dichloromethane	Good	9 µg/ml IC_50_	*Plasmodium falciparum* W2	No
*Leucas calostachys*	*Lamiaceae*	[95]	Kenya	Whole Plant	Water	Very Good	0.79 µg/ml IC_50_	*Plasmodium Knowlesi*	Nd
*Leucas martinicensis*	*Lamiaceae*	[22]	South Africa	Whole Plant	Dichloromethane/Methanol	Moderate	13.3 µg/ml IC_50_	*Plasmodium falciparum* D10	Nd
*Lippia javanica*	*Verbenaceae*	[24]	Kenya	Root Barks	Methanol	Good	5.9 µg/ml IC_50_	*Plasmodium falciparum* D6, W2	Nd
		[104]	Kenya	Roots	Dichloromethane/Ethyl Acetate	Moderate	16.7—19.2 µg/ml IC_50_	*Plasmodium falciparum* K39, V1/S	Nd
		[22]	South Africa	Roots	Dichloromethane	Very Good	3.8 µg/ml IC_50_	*Plasmodium falciparum* D10	Nd
		[25]	South Africa	Leaves	Acetone	Very Good	4.26 µg/ml IC_50_	*Plasmodium falciparum* UP1 (CQ-R)	Nd
*Lippia multiflora*	*Verbenaceae*	[57]	Ivory Coast	Leaves	Water	Very Good	1.18—2.34 µg/ml IC_50_	*Plasmodium falciparum* Fcb1 & F32	Nd
*Lophira lanceolata*	*Ochnaceae*	[52]	Burkina Faso	Leaves	Dichloromethane	Very Good	4.7 µg/ml IC_50_	*Plasmodium falciparum*	Nd
*Ludwigia erecta*	*Onagraceae*	[24]	Kenya	Whole plant	Methanol	Very Good	0.9–1.6 µg/ml IC_50_	*Plasmodium falciparum* D6, W2	Nd
*Macrostylis squarrosa*	*Rutaceae*	[22]	South Africa	Stems	Dichloromethane/Methanol	Moderate	16 µg/ml IC_50_	*Plasmodium falciparum* D10	Nd
*Maesa lanceolata*	*Primulaceae*	[22]	South Africa	Twigs	Dichloromethane/Methanol	Good	5.9 µg/ml IC_50_	*Plasmodium falciparum* D10	Nd
*Markhamia lutea*	*Bignognaceae*	[76]	Uganda	Leaves	Ethyl Acetate	Nd	Nd	*Plasmodium falciparum* Fcb6	Nd
*Maytenus heterophylla*	*Celastraceae*	[24]	Kenya	Root barks	Methanol	Very Good	1.8–3.9 µg/ml IC_50_	P*lasmodium falciparum* D6, W2	Nd
*Maytenus obtusifolia*	*Celastraceae*	[24]	Kenya	Root barks	Methanol	Good	< 1.9–5.8 µg/ml IC_50_	*Plasmodium falciparum* D6, W2	Nd
*Maytenus putterlickioides*	*Celastraceae*	[26]	Kenya	Root Barks	Methanol	Good	4.4–10.2 µg/ml IC_50_	*Plasmodium falciparum* D6, W2	Nd
*Maytenus senegalensis*	*Celastraceae*	[58]	Sudan	Stem barks	Methanol	Nd	3.9–10 µg/ml IC_50_	*Plasmodium falciparum* 3D7, Dd9	Nd
		[26]	Kenya	Root barks	Methanol	Good	4.7–9.8 µg/ml IC_50_	*Plasmodium falciparum* D6, W2	Nd
		[22]	South Africa	Roots	Dichloromethane	Moderate	15.5 µg/ml IC_50_	*Plasmodium falciparum* D10	Nd
		[20]	Tanzania	Stem barks	Ethyl Acetate	Very Good	0.16 µg/ml IC_50_	*Plasmodium falciparum* K1	Nd
		[31]	Tanzania	Roots	Ethanol	Very Good	2.05 µg/ml IC_50_	*Plasmodium falciparum* K1	No
*Maytenus undata*	*Celastraceae*	[26]	Kenya	Leaves	Water	Very Good	0.95–1.9 µg/ml IC_50_	*Plasmodium falciparum* D6, W2	Nd
Melia azedarach	*Meliaceae*	[46]	D.R. Congo	Leaves	Dichloromethane	Moderate	19.14 µg/ml IC_50_	*Plasmodium falciparum* 3D7, W2, *Plasmodium berghei berghei*	Nd
*Microdesmis keayana*	*Pandaceae*	[51]	Ivory Coast	Leaves	Methylene Chloride	Moderate	12.2 µg/ml IC_50_	*Plasmodium falciparum* K1	No
*Microglossa pyrifolia*	*Asteraceae*	[24]	Kenya	Leaves	Methanol	Moderate	10.4 µg/ml IC_50_	*Plasmodium falciparum* D6, W2	Nd
		[77]	Uganda	Leaves	Ethyl Acetate	Very Good	0.03–0.05 µg/ml IC_50_	*Plasmodium falciparum* NF54 & FCR3	Nd
		[92]	Rwanda	Leaves	Dichloromethane	Very Good	1.5–2.4 µg/ml IC_50_	*Plasmodium falciparum* 3D7, W2	Yes (SI = 3.2; human normal foetal lungfibroblast [WI-38])
*Mikania cordata*	*Compositae*	[20]	Tanzania	Leaves	Ethyl Acetate	Moderate	14 µg/ml IC_50_	*Plasmodium falciparum* K1	Nd
*Millettia zechiana*	*Fabaceae*	[28]	Ivory Coast	Stem Barks	Ethanol	Moderate	16.1 µg/ml IC_50_	*Plasmodium falciparum* Fcb1/Colombia Strain	Nd
		[43]	Ivory Coast	Stem Barks	Ethanol	Moderate	14.1 µg/ml IC_50_	*Plasmodium falciparum* FCB1	Nd
*Momordica balsamina*	*Cucurbitaceae*	[22]	South Africa	Stems	Dichloromethane/Methanol	Good	5.3 µg/ml IC_50_	*Plasmodium falciparum* D10	Nd
		[68]	Mozambique	Aerial Parts	Ns	Very Good^a^	1 μM	*Plasmodium berghei, Plasmodium falciparum*	Nd
*Momordica charantia*	*Cucurbitaceae*	[88]	Nigeria	Leaves	Methanol	Very Good	12.5 nM	*Plasmodium falciparum*	Nd
*Momordica foetida*	*Cucurbitaceae*	[77]	Uganda	Leaves	Water	Good	0.35–6.16 µg/ml IC_50_	*Plasmodium falciparum* NF54 & FCR3	Nd
*Monodora myristica*	*Annonaceae*	[33]	Gabon	Stem	Methanol	Good	5.5–6.1 µg/ml IC_50_	*Plasmodium falciparum* Fcbm W2	No
		[49]	Cameroon	Leaves	Methanol	Good	9.03 µg/ml IC_50_	*Plasmodium falciparum* W2	Nd
*Morinda lucida*	*Rubiaceae*	[74]	S. Tome´ And Prı ´Ncipe	Barks	Ethanol	Good	< 10 µg/ml IC_50_	*Plasmodium falciparum* 3D7 and Dd2	Nd
		[88]	Nigeria	Leaves	Methanol	Very Good	25 nM	*Plasmodium falciparum*	Nd
		[53]	Nigeria	Roots	Dichloromethane	Moderate	13.37 µg/ml IC_50_	*Plasmodium falciparum* D10	Nd
*Morinda morindoides*	*Rubiaceae*	[43]	Ivory Coast	Leaves	Ethanol	Good	9.8 µg/ml IC_50_	*Plasmodium falciparum* FCB1	Nd
		[28]	Ivory Coast	Leaves	Ethanol	Moderate	11.6 µg/ml IC_50_	*Plasmodium falciparum* Fcb1/Colombia Strain	Nd
*Moringa oleifera*	*Moringaceae*	[26]	Kenya	Leaves	Methanol	Moderate	9.8 µg/ml IC_50_	*Plasmodium falciparum* D6, W2	Nd
*Motandra guineensis*	*Apocynaceae*	[43]	Ivory Coast	Leaves	Ethanol	Moderate	16.3 µg/ml IC_50_	*Plasmodium falciparum* FCB1	Nd
*Mundulea sericea*	*Fabaceae*	[86]	Namibia	Leaves, Shoots	Methanol	Very Good	3.279–3.352 µg/ml IC_50_	*Plasmodium falciparum* 3D7	Nd
*Mitragyna inermis*	*Rubiaceae*	[93]	Mali	Leaves	Chloroform	Very Good	4.36–4.82 µg/ml IC_50_	*Plasmodium falciparum* W2 & 3D7	No
*Nauclea latifolia*	*Rubiaceae*	[93]	Mali	Barks	Chloroform	Good	5.36–6.2 µg/ml IC_50_	*Plasmodium falciparum* W2 & 3D7	Yes (IC_50_ = 50 µg/ml; BALB/C mouse)
		[28]	Ivory Coast	Barks	Ethanol	Good	8.9 µg/ml IC_50_	*Plasmodium falciparum* Fcb1/Colombia Strain	No
		[106]	Ivory Coast	Roots, Stem	Water	Good	0.6–7.5 µg/ml IC_50_	*Plasmodium falciparum* Fcb1- Colombian And Nigerian Strains	Nd
		[43]	Ivory Coast	Root Barks	Ethanol	Good	7.3 µg/ml IC_50_	*Plasmodium falciparum* FCB1	Nd
*Nauclea pobeguinii*	*Rubiaceae*	[107]	D.R.Congo	Stem Barks	Ethanol	In Active	32 µg/ml IC_50_	*Plasmodium falciparum, Plasmodium yeolii, Plasmodium berghei*	No
*Neoboutonia glabrescens*	*Euphorbiaceae*	[55]	Cameroon	Leaves	Ethanol, Water, Dichloromethane, Methanol, Hexane	Good	7.56 µg/ml IC_50_	*Plasmodium falciparum* W2	Nd
*Neorautanenia mitis*	*Fabaceae*	[31]	Tanzania	Tubers	Ethanol	Very Good	1.58 µg/ml IC_50_	*Plasmodium falciparum* K1	No
*Newbouldia laevis*	*Bignognaceae*	[108]	Togo	Leaves	Ethanol	Moderate	12.6 µg/ml IC_50_	*Plasmodium falciparum*	Nd
		[109]	Nigeria	Leaves	Water	Moderate	19.5 µg/ml IC_50_	*Plasmodium falciparum*	Nd
		[53]	Nigeria	Roots	Dichloromethane	Good	5.00 µg/ml IC_50_	*Plasmodium falciparum* D10	Nd
*Ocimum americana*	*Lamiaceae*	[24]	Kenya	Whole Plant	Methanol	Moderate	8.9–12.1 µg/ml IC_50_	*Plasmodium falciparum* D6, W2	Nd
		[22]	South Africa	Whole Plant	Dichloromethane/Methanol	Very Good	4.2 µg/ml IC_50_	*Plasmodium falciparum* D10	Nd
*Ocimum basilicum*	*Lamiaceae*	[159]	D.R. Congo	Leaves	Ethanol, Methanol, Petroleum Ether, Chloroform	Good	< 0.35–18 µg/ml IC_50_	*Plasmodium falciparum*	Nd
		[26]	Kenya	Leaves	Methanol	Moderate	16.4 µg/ml IC_50_	*Plasmodium falciparum* D6, W2	Nd
*Ocimum gratissimum*	*Lamiaceae*	[30]	Kenya	Leaves, Twigs	Dichloromethane	Good	8.616 µg/ml IC_50_	*Plasmodium falciparum* W2, D6	Nd
		[40]	DR Congo	Leaves	Water	Good	7.25 µg/ml IC_50_	*Plasmodium falciparum* K1	No
		[26]	Kenya	Leaves	Methanol	Good	5.9 µg/ml IC_50_	*Plasmodium falciparum* D6, W2	Nd
*Ocimum kilimandscharicum*	*Lamiaceae*	[30]	Kenya	Leaves, Twigs	Dichloromethane	Very Good	0.843–1.547 µg/ml IC_50_	*Plasmodium falciparum* W2, D6	Nd
*Olax gambecola*	*Olacaceae*	[43]	Ivory Coast	Whole Plant	Ethanol	Good	5.2 µg/ml IC_50_	*Plasmodium falciparum* FCB1	Nd
*Olea europaea*	*Oleaceae*	[24]	Kenya	Stem Barks	Methanol	Moderate	17.3 µg/ml IC_50_	*Plasmodium falciparum* D6, W2	Nd
		[21]	Kenya	Inner Barks	Methanol	Good	9.48 µg/ml IC_50_	*Plasmodium falciparum* D6	No
		[22]	South Africa	Leaves	Dichloromethane/Methanol	Moderate	12 µg/ml IC_50_	*Plasmodium falciparum* D10	Nd
*Opilia celtidifolia*	*Opiliaceae*	[52]	Burkina Faso	Leaves	Dichloromethane	Very Good	2.8 µg/ml IC_50_	*Plasmodium falciparum*	Yes (SI = 0.4; HepG2 cells)
*Ormocarpum trachycarpum*	*Fabaceae*	[77]	Kenya	Stem Barks	Dichloromethane/Ethyl Acetate	Moderate	17.5—19.6 µg/ml IC_50_	*Plasmodium falciparum* K39, V1/S	Nd
*Osteospermum imbricatum*	*Asteraceae*	[22]	South Africa	Stems	Dichloromethane/Methanol	Good	7.3 µg/ml IC_50_	*Plasmodium falciparum* D10	Nd
*Phyllanthus amarus*	*Phyllanthaceae*	[53]	Nigeria	Leaves	Petroleum Ether	Very Good	4.99 µg/ml IC_50_	*Plasmodium falciparum* D10	Nd
*Pachypodanthium confine*	*Annonaceae*	[98]	Cameroon	Stem Barks	Water	Moderate^a^	16.6 µg/ml IC_50_	*Plasmodium falciparum* W3	Nd
*Pappea capensis Eckl.& Zeyh*	*Sapindaceae*	[37]	South Africa	Twigs	Dichloromethane	Good	5.47 µg/ml IC_50_	*Plasmodium falciparum* NF54	Nd
*Parinari curatellifolia*	*Chrysobalanaceae*	[22]	South Africa	Roots	Dichloromethane	Good	5.3 µg/ml IC_50_	*Plasmodium falciparum* D10	Nd
		[24]	Kenya	Root Barks	Methanol	Good	3.9–7.9 µg/ml IC_50_	*Plasmodium falciparum* D6, W2	Nd
		[37]	South Africa	Stem Barks	Dichloromethane	Good	6.99 µg/ml IC_50_	*Plasmodium falciparum* NF54	Nd
*Parinari excelsa*	*Chrysobalanaceae*	[20]	Tanzania	Stem Barks	Ethyl Acetate	Moderate	10 µg/ml IC_50_	*Plasmodium falciparum* K1	Nd
		[75]	Uganda	Barks	Ethyl Acetate	Nd	Nd	*Plasmodium falciparum* Fcb7	Nd
*Parkinsonia aculeata*	*Fabaceae*	[22]	South Africa	Twigs	Dichloromethane/Methanol	Good	9 µg/ml IC_50_	*Plasmodium falciparum* D10	Nd
*Pavetta corymbosa*	*Rubiaceae*	[65]	Benin	Aerial parts	Methanol	Moderate	5.54–20 µg/ml IC_50_	*Plasmodium falciparum* 3D7 & K1	Nd
		[110]	Togo	Aerial parts	Methanol	Very Good	2.042 µg/ml IC_50_	*Plasmodium falciparum*	Nd
		[110]	Togo	Aerial part	Methanol	Very Good	2.042 µg/ml IC_50_	*Plasmodium falciparum*	Nd
*Pavetta crassipes*	*Rubiaceae*	[27]	Burkina Faso	Leaves	Crude Alkaloid	Very Good	< 4 µg/ml IC_50_	*Plasmodium falciparum* W2	Nd
		[71]	Togo	Aerial parts	Water	Good	< 7 µg/ml IC_50_	*Plasmodium falciparum*	Nd
*Pelargonium alchemilloides*	*Geraniaceae*	[22]	South Africa	Whole plant	Dichloromethane/Methanol	Moderate	15 µg/ml IC_50_	*Plasmodium falciparum* D10	Nd
*Pentas lanceolata*	*Rubiaceae*	[21]	Kenya	Root barks	Methanol	Good	5.15 µg/ml IC_50_	*Plasmodium falciparum* D6	No
*Pentas longiflora*	*Rubiaceae*	[26]	Kenya	Root barks	Methanol	Moderate	13.3 µg/ml IC50	*Plasmodium falciparum* D6, W2	Nd
*Pentzia globosa*	*Asteraceae*	[22]	South Africa	Roots	Dichloromethane	Good	8 µg/ml IC50	*Plasmodium falciparum* D10	Nd
*Phyllanthus amarus*	*Phyllanthaceae*	[111]	Ghana	Whole Plant	Ethanol	Moderate	11.7 µg/ml IC_50_	*Plasmodium falciparum* Dd2	No
*Phyllanthus fraternus*	*Phyllanthaceae*	[112]	Ghana	Whole plant	Methanol	Very Good	0.44 µg/ml IC_50_	*Plasmodium falciparum* 3D7, W2	No
*Phyllanthus muellerianus*	*Phyllanthaceae*	[28]	Ivory Coast	Leaves	Ethanol	Good	9.4 µg/ml IC_50_	*Plasmodium falciparum* Fcb1/Colombia Strain	No
		[43]	Ivory Coast	Leaves	Ethanol	Moderate	10.3 µg/ml IC_50_	*Plasmodium falciparum* FCB1	Nd
*Phyllanthus niruri*	*Phyllanthaceae*	[69]	D.R.Congo	Whole Plant	Petroleum Ether	Very Good	1.3 µg/ml IC_50_	*Plasmodium falciparum*	Nd
*Phyllanthus urinaria*	*Phyllanthaceae*	[45]	Cambodia	Whole Plant	Water	Very Good	2.4 µg/ml IC_50_	*Plasmodium falciparum* W2	Nd
*Physalis angulata*	*Solanaceae*	[28]	Ivory Coast	Whole Plant	Ethanol	Good	7.9 µg/ml IC_50_	*Plasmodium falciparum* Fcb1/Colombia Strain	Nd
		[43]	Ivory Coast	Whole Plant	Ethanol	Good	7.9 µg/ml IC_50_	*Plasmodium falciparum* FCB1	Nd
		[44]	D.R. Congo	Leaves	Methanolic and dichloromethane	Very good	1.27 µg/ml IC_50_	*Plasmodium falciparum* 3D7, W2, *Plasmodium berghei berghei*	No
*Picralima nitida*	*Apocynaceae*	[53]	Nigeria	Roots	Ethanol	Good	6.29 µg/ml IC_50_	*Plasmodium falciparum* D10	Nd
		[113]	Nigeria	Stems	Methanol	Good	6.0–6.3 µg/ml IC_50_	*Plasmodium falciparum* D6, W2	No
		[89]	Cameroon	Seeds	Methanol	Moderate	10.9 µg/ml IC_50_	*Plasmodium falciparum* W2	Nd
		[114]	Ivory Coast	Root, Stem Barks Ans Fruit Rins	Ns	Very Good	0.188–1.581 μg/ml IC_50_	*Plasmodium falciparum*	Nd
*Piper capense*	*Piperaceae*	[91]	Comoros	Ns	Dichloromethane	Good	7 µg/ml IC_50_	*Plasmodium falciparum* W2	No
*Piptadeniastrum africanum*	*Leguminosae*	[40]	D.R. Congo	Stem Barks	Water	Good	6.11 µg/ml IC_50_	*Plasmodium falciparum* K1	Yes (SI = 1.4–1.5; human embryonic lung cells [MRC-5])
		[40]	D.R.Congo	Stem Barks	Water	Good	6.11 µg/ml IC_50_	*Plasmodium falciparum* K1	No
*Piptostigma calophyllum*	*Annonaceae*	[49]	Cameroon	Leaves	Methanol	Good	6.72 µg/ml IC_50_	*Plasmodium falciparum* W2	Nd
*Pittosporum viridiflorum*	*Pittosporaceae*	[24]	Kenya	Leaves	Methanol	Moderate	17.6–18.9 µg/ml iC_50_	*Plasmodium falciparum* D6, W2	Nd
		[22]	South Africa	Whole Plant	Dichloromethane	Very Good	3 µg/ml IC_50_	*Plasmodium falciparum* D10	Nd
*Plumbago zeylanica*	*Plumbaginaceae*	[22]	South Africa	Leaves	Dichloromethane	Very Good	3 µg/ml IC_50_	*Plasmodium falciparum* D10	Nd
*Podocarpus latifolius*	*Podocarpaceae*	[21]	Kenya	Root Barks	Methanol	Good	6.43 µg/ml IC_50_	*Plasmodium falciparum* D6	No
*Pollichia campestris*	*Caryophyllaceae*	[22]	South Africa	Twigs	Dichloromethane/Methanol	Good	6.8 µg/ml IC_50_	*Plasmodium falciparum* D10	Nd
*Polyalthia longifolia*	*Annonaceae*	[115]	Ghana	Stem Barks	Ethanol, N-Hexane,Dichloromethane, Ethyl Acetate, Methanol-Ethyl Acetate	Good^a^	3–6 µg/ml IC_50_	*Plasmodium falciparum* K1	No
		[116]	Ghana	Stem Barks	Methanol, Chloroform, Cyclohexane, Ethyl Acetate	Good^a^	4.53–10.17 µM IC_50_	*Plasmodium falciparum* 3D8	Nd
*Polyalthia oliveri*	*Annonaceae*	[55]	Cameroon	Stem Barks	Ethanol, Water, Dichloromethane, Methanol, Hexane	Very Good	4.30 µg/ml IC_50_	*Plasmodium falciparum* W2	Nd
		[49]	Cameroon	Stem Barks	Methanol	Very Good	3.43 µg/ml IC_50_	*Plasmodium falciparum* W2	Nd
*Polyalthia suaveolens*	*Annonaceae*	[49]	Cameroon	Twigs	Methanol	Very Good	3.23 µg/ml IC_50_	*Plasmodium falciparum* W2	Nd
*Polygonatum verticillatum*	*Asparagaceae*	[117]	Kenya	Rhizome	N-Hexane, Chloroform	Very Good	2.33—4.62 μg/ml IC_50_	*Plasmodium* *falciparum*	No
*Premna chrysoclada*	*Lamiaceae*	[26]	Kenya	Leaves	Methanol	Moderate	11.1 µg/ml IC_50_	*Plasmodium falciparum* D6, W2	Nd
*Prosopis africana*	*Fabaceae*	[50]	Nigeria	Ns	Methanol, Water, Butanol, Ethyl Acetate	Moderate	14.97–15.28 µg/ml IC_50_	*Plasmodium falciparum* 3D7, K1	Yes (SI ≥ 99; mouse heart-derived cells [NBMH])
*Prunus africana*	*Rosaceae*	[24]	Kenya	Stem Barks	Methanol	Moderate	17.3 µg/ml IC_50_	*Plasmodium falciparum* D6, W2	Nd
*Pseudospondias microcarpa*	*Anacardiaceae*	[31]	Tanzania	Roots	Ethanol	Very Good	1.13 µg/ml IC_50_	*Plasmodium falciparum* K1	No
*Psiadia punctulata*	*Asteraceae*	[22]	South Africa	Twigs	Dichloromethane	Good	9 µg/ml IC_50_	*Plasmodium falciparum* D10	Nd
*Psidium guajava*	*Myrtaceae*	[40]	DR Congo	Leaves	Water	Good	5.46 µg/ml IC_50_	*Plasmodium falciparum* K1	No
		[20]	Tanzania	Leaves	Ethyl Acetate	Moderate	10 µg/ml IC_50_	*Plasmodium falciparum* K1	Nd
*Psorospermum senegalense*	*Hypericaceae*	[63]	Burkina Faso	Leaves	Dichloromethane	Moderate	10.03 µg/ml IC_50_	*Plasmodium falciparum* 3D7 & W2	No
*Ptaeroxylon obliquum*	*Rutaceae*	[22]	South Africa	Stems	Dichloromethane/Methanol	Good	5.5 µg/ml IC_50_	*Plasmodium falciparum* D10	Nd
*Pterocarpus angolensis*	*Fabaceae*	[22]	South Africa	Roots	Dichloromethane	Moderate	10.6 µg/ml IC_50_	*Plasmodium falciparum* D10	Nd
*Pterocarpus erinaceus*	*Fabaceae*	[118]	Burkina Faso	Leaves Ans Barks	Ethanol, Chloroform	Very Good	1.93 µg/ml IC_50_	*Plasmodium falciparum* 3D7 And Dd2	Nd
*Pulicaria crispa*	*Asteraceae*	[34]	Sudan	Whole Plant	Petroleum Ether/Chloroform	Very Good	< 5 µg/ml IC_50_	*Plasmodium falciparum*	Nd
*Pycnanthus angolensis*	*Myristicaceae*	[28]	Ivory Coast	Stem Barks	Ethanol	Moderate	18.2 µg/ml IC_50_	*Plasmodium falciparum* Fcb1/Colombia Strain	Nd
		[74]	S. Tome´ And Prı ´Ncipe	Barks	Ethanol	Very Good	< 5 µg/ml IC_50_	*Plasmodium falciparum* 3D7 And Dd2	Nd
*Pyrenacantha grandiflora Baill*	*Icacinaceae*	[37]	South Africa	Roots	Dichloromethane	Good	5.82 µg/ml IC_50_	*Plasmodium falciparum* NF54	Nd
*Quassia africana*	*Simaroubaceae*	[103]	Congo Brazzaville	Leaves	Water, Ethanol, Dichloromethane	Very Good	0.1–2.2 µg/ml IC_50_	*Plasmodium falciparum* Fcm29-Cameroon	Yes (IC_50_ = 6.7 µg/ml; KB cells)
		[40]	D.R. Congo	Root Barks	Water	Very Good	0.46 µg/ml IC_50_	*Plasmodium falciparum* K1	No
*Ranunculus multifidus*	*Ranunculaceae*	[22]	South Africa	Whole Plant	Dichloromethane/Methanol	Very Good	2.3 µg/ml IC_50_	*Plasmodium falciparum* D10	Nd
*Rauvolfia caffra Sond*	*Apocynaceae*	[37]	South Africa	Roots	Dichloromethane	Very Good	2.13 µg/ml IC_50_	*Plasmodium falciparum* NF54	Nd
*Rauvolfia nombasiana*	*Apocynaceae*	[26]	Kenya	Root Barks	Methanol	Good	9.1 µg/ml IC_50_	*Plasmodium falciparum* D6, W2	Nd
*Rauvolfia vomitoria*	*Apocynaceae*	[53]	Nigeria	Roots	Dichloromethane	Very Good	4.78 µg/ml IC_50_	*Plasmodium falciparum* D10	Nd
		[28]	Ivory Coast	Barks	Ethanol	Very Good	2.5 µg/ml IC_50_	*Plasmodium falciparum* Fcb1/Colombia Strain	No
		[43]	Ivory Coast	Root Barks	Ethanol	Very Good	2.5 µg/ml IC_50_	*Plasmodium falciparum* FCB1	Nd
*Rhamnus prinoides*	*Rhamnaceae*	[77]	Kenya	Roots	Methanol	Moderate	15.1 µg/ml IC_50_	*Plasmodium falciparum* K39 (CQ-S), ENT30, NF54, V1/S	Nd
		[21]	Kenya	Root Barks	Methanol	Very Good	3.53 µg/ml IC_50_	*Plasmodium falciparum* D6	No
*Rhizophora mucronata*	*Rhizophoraceae*	[22]	South Africa	Twigs	Dichloromethane/Methanol	Good	5.6 µg/ml IC_50_	*Plasmodium falciparum* D10	Nd
*Ricinus communis var. communis*	*Euphorbiaceae*	[22]	South Africa	Stems	Water	Good	8.0 µg/ml IC_50_	*Plasmodium falciparum* D10	Nd
*Rubia cordifolia*	*Rubiaceae*	[95]	Kenya	Leaves/Seeds/Stems	Methanol	Very Good	1.20 µg/ml IC_50_	*Plasmodium Knowlesi*	Nd
		[24]	Kenya	Whole Plant	Methanol	Very Good	< 5 µg/ml IC_50_	*Plasmodium falciparum* D6, W2	Nd
*Rumex abyssinicus*	*Polygonaceae*	[92]	Rwanda	Roots	Water	Very Good	3.1–4.3 µg/ml IC_50_	*Plasmodium falciparum* 3D7, W2	Yes (SI = 3.1; human normal foetal lung fibroblast [WI-38])
*Rumex crispus*	*Polygonaceae*	[22]	South Africa	Roots	Dichloromethane	Moderate	14 µg/ml IC_50_	*Plasmodium falciparum* D10	Nd
*Salacia madagascariensis*	*Celastraceae*	[20]	Tanzania	Roots	Petroleum Ether	Very Good	0.8 µg/ml IC_50_	*Plasmodium falciparum* K1	Nd
*Salvia africana-lutea*	*Lamiaceae*	[120]	South Africa	Aerial Parts	Methanol/Chloroform	Moderate	15.863 µg/ml IC_50_	*Plasmodium falciparum* FCR-3	Nd
*Salvia albicaulis*	*Lamiaceae*	[120]	South Africa	Aerial Parts	Methanol/Chloroform	Moderate	15.833 µg/ml IC_50_	*Plasmodium falciparum* FCR-3	Nd
*Salvia aurita*	*Lamiaceae*	[120]	South Africa	Aerial Parts	Methanol/Chloroform	Good	8.923 µg/ml IC_50_	*Plasmodium falciparum* FCR-3	Nd
*Salvia chamelaeagnea*	*Lamiaceae*	[120]	South Africa	Aerial Parts	Methanol/Chloroform	Good	8.713 µg/ml IC_50_	*Plasmodium falciparum* FCR-3	Nd
*Salvia dolomitica*	*Lamiaceae*	[120]	South Africa	Aerial Parts	Methanol/Chloroform	Good	7.623 µg/ml IC_50_	*Plasmodium falciparum* FCR-3	Nd
*Salvia garipensis*	*Lamiaceae*	[120]	South Africa	Aerial Parts	Methanol/Chloroform	Moderate	13.953 µg/ml IC_50_	*Plasmodium falciparum* FCR-3	Nd
*Salvia muirii*	*Lamiaceae*	[120]	South Africa	Aerial Parts	Methanol/Chloroform	Moderate	11.873 µg/ml IC_50_	*Plasmodium falciparum* FCR-3	Nd
*Salvia radula*	*Lamiaceae*	[120]	South Africa	Aerial Parts	Methanol/Chloroform	Very Good	3.913 µg/ml IC_50_	*Plasmodium falciparum* FCR-3	Yes (IC_50_ = 20.12 µg/ml; Kidney cells)
*Salvia repens*	*Lamiaceae*	[120]	South Africa	Aerial Parts	Methanol/Chloroform	Good	8.253 µg/ml IC_50_	*Plasmodium falciparum* FCR-3	Nd
		[22]	South Africa	Whole Plant	Dichloromethane/Methanol	Moderate	10.8 µg/ml IC_50_	*Plasmodium falciparum* D10	Nd
*Salvia runcinata*	*Lamiaceae*	[120]	South Africa	Aerial Parts	Methanol/Chloroform	Moderate	16.613 µg/ml IC_50_	*Plasmodium falciparum* FCR-3	Nd
*Salvia schlechteri*	*Lamiaceae*	[120]	South Africa	Aerial Parts	Methanol/Chloroform	Moderate	17.513 µg/ml IC_50_	*Plasmodium falciparum* FCR-3	Nd
*Salvia stenophylla*	*Lamiaceae*	[120]	South Africa	Aerial Parts	Methanol/Chloroform	Good	6.53 µg/ml IC_50_	*Plasmodium falciparum* FCR-3	Yes (IC_50_ = 12.12 µg/ml; Kidney cells)
*Sonchus schweinfurthi*	*Compositae*	[95]	Kenya	Barks/Roots	Methanol	Very Good	2.10 µg/ml IC_50_	*Plasmodium Knowlesi*	Nd
*Scaevola plumieri*	*Goodeniaceae*	[22]	South Africa	Twigs	Dichloromethane	Moderate	11 µg/ml IC_50_	*Plasmodium falciparum* D10	Nd
*Schefflera umbellifera*	*Araliaceae*	[22]	South Africa	Leaves	Dichloromethane/Methanol	Very Good	3.7 µg/ml IC_50_	*Plasmodium falciparum* D10	Nd
*Schizozygia coffaeoides*	*Apocynaceae*	[26]	Kenya	Leaves	Methanol	Moderate	10.5 µg/ml IC_50_	*Plasmodium falciparum* D6, W2	Nd
*Schkuhria pinnata*	*Compositae*	[24]	Kenya	Whole Plant	Methanol	Good	1.3–6.8 µg/ml IC_50_	*Plasmodium falciparum*D6, W2	Nd
*Schrankia leptocarpa*	*Fabaceae*	[65]	Benin	Aerial Parts	Methanol	Moderate	3.38- > 20 µg/ml IC_50_	*Plasmodium falciparum* 3D7 & K1	Nd
*Sclerocarya birrea*	*Anacardiaceae*	[24]	Kenya	Stem Barks	Methanol	Moderate	5.9–24.9 µg/ml IC_50_	*Plasmodium falciparum* D6, W2	Nd
*Secamone afzelii*	*Apocynaceae*	[65]	Benin	Aerial Parts	Methanol	Moderate	6.48- > 20 µg/ml IC_50_	*Plasmodium falciparum* 3D7 & K1	Nd
*Securidaca longipedunculata*	*Polygalaceae*	[121]	Mali	Leaves	Dichloromethane	Good	6.9 µg/ml IC_50_	*Plasmodium falciparum* 3D7	Nd
*Securinega virosa*	*Phyllanthaceae*	[52]	Burkina Faso	Leaves	Dichloromethane	Good	7.1 µg/ml IC_50_	*Plasmodium falciparum*	Nd
*Senecio oxyriifolius*	*Asteraceae*	[22]	South Africa	Whole plant	Dichloromethane/Methanol	Moderate	13 µg/ml IC_50_	*Plasmodium falciparum* D10	Nd
*Senecio stuhlmannii*	*Asteraceae*	[56]	Uganda	Shoots	Ethyl Acetate	Moderate	14.0–15.2 µg/ml IC_50_	*Plasmodium falciparum* D10, K1	Nd
*Senna didymobotrya*	*Fabaceae*	[22]	South Africa	Twigs	Dichloromethane/Methanol	Good	9.5 µg/ml IC_50_	*Plasmodium falciparum* D10	Nd
*Senna petersiana*	*Fabaceae*	[22]	South Africa	Twigs	Dichloromethane/Methanol	Moderate	13 µg/ml IC_50_	*Plasmodium falciparum* D10	Nd
		[59]	Malawi	Leaves	Methanol	Very Good	2·67 µg/ml IC_50_	*Plasmodium falciparum* Vl/S	Nd
*Sericocomopsis hildebrandtii*	*Amaranthaceae*	[21]	Kenya	Root Barks	Methanol	Very Good	3.78 µg/ml IC_50_	*Plasmodium falciparum* D6	No
*Setaria megaphylla*	*Poaceae*	[22]	South Africa	Whole plant	Dichloromethane/Methanol	Very Good	4.5 µg/ml IC_50_	*Plasmodium falciparum* D10	Nd
*Sida acuta*	*Malvaceae*	[118]	Burkina Faso	Whole Plant	Ethanol, Chloroform, Water	Very Good	0.87–0.92 µg/ml IC_50_	*Plasmodium falciparum* 3D7 And Dd2	Nd
		[38]	Ivory Coast	Ns	Ethanol	Good	3.9–5.4 µg/ml IC_50_	*Plasmodium falciparum*	No
*Solanum panduriforme*	*Solanaceae*	[25]	South Africa	Leaves	Acetone	Very Good	3.62 µg/ml IC_50_	*Plasmodium falciparum* UP1 (CQ-R)	Nd
*Solanecio mannii*	*Asteraceae*	[92]	Rwanda	Leaves	Dichloromethane	Moderate	12.7–18.2 µg/ml IC_50_	*Plasmodium falciparum* 3D7, W2	No
*Spilanthes mauritiana*	*Asteraceae*	[22]	South Africa	Stems	Dichloromethane/Methanol	Good	5.3 µg/ml IC_50_	*Plasmodium falciparum* D10	Nd
*Staudtia gabonensis*	*Myristicaceae*	[33]	Gabon	Stems	Methanol	Very Good	0.8 µg/ml IC_50_	*Plasmodium falciparum* Fcbm W2	No
*Stephania abyssinica*	*Menispermaceae*	[24]	Kenya	Root Barks	Methanol	Good	4.7–6.1 µg/ml IC_50_	*Plasmodium falciparum* D6, W2	Nd
*Stephania rotunda*	*Menispermaceae*	[45]	Cambodia	Tubers	Dichloromethane	Very Good	1.0 µg/ml IC_50_	*Plasmodium falciparum* W2	Nd
*Struchium sparganophorum*	*Asteraceae*	[73]	S. Tome´ And Prı ´Ncipe	Leaves	Petroleum Ether	Good	< 10 µg/ml IC_50_	*Plasmodium falciparum* 3D7 And Dd2	Nd
*Strychnopsis thouarsii*	*Menispermaceae*	[122]	Madagascar	Stem Barks	Methanol	Very Good^a^	3.1—4.2 µM	*Plasmodium falciparum* NF54, *Plasmodium yoelli* 265 BY	No
*Strychnos henningsii*	*Loganiaceae*	[72]	Kenya	Twigs	Methanol	Moderate	14.6–17.9 µg/ml IC_50_	*Plasmodium falciparum* K1, NF54	Nd
*Strychnos pungens*	*Loganiaceae*	[22]	South Africa	Leaves	Dichloromethane	Moderate	12.6 µg/ml IC_50_	*Plasmodium falciparum* D10	Nd
*Strychnos spinosa*	*Loganiaceae*	[123]	Senegal	Leaves, Stem	Methanol, Water	Moderate	15 µg/ml IC_50_	*Plasmodiumfalciparum*	Nd
*Strychnos icaja*	*Loganiaceae*	[46]	D.R. congo	Root barks	Methanolic and dichloromethane	Very good	0.69 µg/ml IC_50_	*Plasmodium falciparum* 3D7, W2, *Plasmodium berghei berghei*	Nd
*Suregada zanzibariensis*	*Euphorbiaceae*	[26]	Kenya	Leaves	Methanol	Good	5.8–6.7 µg/ml IC_50_	*Plasmodium falciparum* D6, W2	Nd
		[124]	Kenya	Leaves	Methanol	Very Good	1.82–4.66 µg/ml IC_50_	*Plasmodium falciparum* D6&W2	Nd
		[124]	Kenya	Leaves	Methanol	Very Good	1.82–4.66 µg/ml IC_50_	*Plasmodium falciparum* D6, W2	No
*Syzygium cordatum subsp. cordatum*	*Myrtaceae*	[22]	South Africa	Twigs	Dichloromethane/Methanol	Moderate	14.7 µg/ml IC_50_	*Plasmodium falciparum* D10	Nd
		[37]	South Africa	Leaves	Dichloromethane	Good	6.15 µg/ml IC_50_	*Plasmodium falciparum* NF54	Nd
*Tabernaemontana elegans*	*Apocynaceae*	[37]	South Africa	Roots	Dichloromethane	Very Good	0.33 µg/ml IC_50_	Plasmodium falciparum NF54	Nd
*Tabernaemontana pachysiphon*	*Apocynaceae*	[26]	Kenya	Flower	Methanol	Very Good	4.4–4.8 µg/ml IC_50_	*Plasmodium falciparum* D6, W2	Nd
*Tagetes minuta*	*Asteraceae*	[75]	Uganda	Leaves	Ethyl Acetate	Nd	Nd	*Plasmodium falciparum* Fcb8	Nd
*Tamarindus indica*	*Fabaceae*	[23]	Sudan	Stem Barks	Methanol	Moderate	10 µg/ml IC_50_	*Plasmodium falciparum* 3D7, Dd2	No
		[110]	Togo	Fruits	Water	Very Good	4.786 µg/ml IC_50_	*Plasmodium falciparum*	Nd
*Tapinanthus dodoneifolius*	*Loranthaceae*	[52]	Burkina Faso	Leaves	Methanol	Good	5.2 µg/ml IC_50_	*Plasmodium falciparum*	Nd
*Tarchonanthus camphoratus*	*Asteraceae*	[22]	South Africa	Whole Plant	Dichloromethane/Methanol	Good	6 µg/ml IC_50_	*Plasmodium falciparum* D10	Nd
*Teclea nobilis*	*Rutaceae*	[24]	Kenya	Stem Barks	Methanol	Moderate	3.9–20.4 µg/ml IC_50_	*Plasmodium falciparum* D6, W2	Nd
		[75]	Uganda	Barks	Ethyl Acetate	Nd	Nd	*Plasmodium falciparum* Fcb9	Nd
*Tecoma capensis*	*Bignoniaceae*	[22]	South Africa	Twigs	Dichloromethane/Methanol	Moderate	10.2 µg/ml IC_50_	*Plasmodium falciparum* D10	Nd
*Tectona grandis*	*Lamiaceae*	[112]	Ghana	Leaves	Methanol	Very Good	0.92 µg/ml IC_50_	*Plasmodium falciparum* 3D7, W2	No
*Terminalia avicennioides*	*Combretaceae*	[50]	Nigeria	Ns	Methanol, Water, Butanol, Ethyl Acetate	Moderate	12.28–14.09 µg/ml IC_50_	*Plasmodium falciparum* 3D7, K1	Yes (SI ≥ 114; mouse heart-derived cells [NBMH])
		[52]	Burkina Faso	Leaves	Methanol	Very Good	1.9 µg/ml IC_50_	*Plasmodium falciparum*	Nd
*Terminalia glaucescens*	*Combretaceae*	[39]	Ivory Coast	Stem, Leave	Water, Ethanol, Pentane	Very Good	2.34–4.83 µg/ml IC_50_	*Plasmodium falciparum* Fcm29, Fcb1, CQ-S (Nigerian)	No
*Terminalia ivorensis*	*Combretaceae*	[32]	Ghana	Stem Barks	Ethanol	Good	6.949 µg/ml IC_50_	*Plasmodium falciparum* 3D7	Nd
		[112]	Ghana	Leaves	Methanol	Good	5.70 µg/ml IC_50_	*Plasmodium falciparum* 3D7, W2	No
*Terminalia macroptera*	*Combretaceae*	[27]	Burkina Faso	Root Barks	Water	Very Good	1 µg/ml IC_50_	*Plasmodium falciparum* W2	Nd
*Terminalia mollis*	*Combretaceae*	[92]	Rwanda	Root Barks	Methanol	Moderate	11.7–26.3 µg/ml IC_50_	*Plasmodium falciparum* 3D7, W2	No
*Terminalia spinosa*	*Combretaceae*	[26]	Kenya	Stem Barks	Methanol	Good	7.9 µg/ml IC_50_	*Plasmodium falciparum* D6, W2	Nd
*Tetracera poggei Gilg*	*Dilleniaceae*	[69]	DR Congo	Leaves	Petroleum Ether	Very Good	1.7 µg/ml IC_50_	*Plasmodium falciparum*	Nd
*Tetrapleura tetraptera*	*Fabaceae*	[33]	Gabon	Leaves	Dichloromethane	Moderate	10.1–13.0 µg/ml IC_50_	*Plasmodium falciparum* FCB, 3D7	No
*Thalia geniculata*	*Marantaceae*	[65]	Benin	Roots	Methanol	Moderate	2.83- > 20 µg/ml IC_50_	*Plasmodium falciparum* 3D7 & K1	Nd
*Tinospora bakis*	*Menispermaceae*	[34]	Sudan	Whole Plant	Petroleum Ether/Chloroform	Very Good	< 5 µg/ml IC_50_	*Plasmodium falciparum*	Nd
*Tithonia diversifolia*	*Asteraceae*	[73]	S. Tome´ And Prı ´Ncipe	Aerial Parts	Petroleum Ether, Dichloromethane	Good	< 10 µg/ml IC_50_	*Plasmodium falciparum* 3D7 And Dd2	Nd
		[92]	Rwanda	Flowers	Dichloromethane	Very Good	1.0–1.1 µg/ml IC_50_	*Plasmodium falciparum* 3D7, W2	No
*Toddalia asiatica*	*Rutaceae*	[26]	Kenya	Root Barks	Methanol	Good	6.82–13.9 µg/ml IC_50_	*Plasmodium falciparum* D6, W2	Nd
		[125]	Kenya	Root Barks	Dichloromethane + Methanol	Very Good^a^	9 – 100 ng/ml IC_50_	*Plasmodium falciparum*	Nd
*Trichilia emetica*	*Meliaceae*	[121]	Mali	Leaves	Dichloromethane	Moderate	11.9 µg/ml IC_50_	*Plasmodium falciparum* 3D7	Nd
		[58]	Sudan	Leaves	Methanol	Good	2.5–17.5 µg/ml IC_50_	*Plasmodium falciparum* 3D7, Dd6	Nd
		[24]	Kenya	Stem Barks	Methanol	Moderate	13.3 µg/ml C_50_	*Plasmodium falciparum* D6, W2	Nd
		[25]	South Africa	Stem Barks	Acetone	Very Good	3.29 µg/ml IC_50_	*Plasmodium falciparum* UP1 (CQ-R)	Nd
		[22]	South Africa	Leaves, Twigs	Dichloromethane/Methanol	Very Good	3.5 µg/ml IC_50_	*Plasmodium falciparum* D10	Nd
*Triclisia dictyophylla*	*Menispermaceae*	[40]	D.R. Congo	Leaves	Water	Good	5.13 µg/ml IC_50_	*Plasmodium falciparum* K1	No
*Tridax procumbens*	*Asteraceae*	[22]	South Africa	Whole Plant	Dichloromethane/Methanol	Moderate	17 µg/ml IC_50_	*Plasmodium falciparum* D10	Nd
		[26]	Kenya	Whole Plant	Methanol	Moderate	15.4 µg/ml IC_50_	*Plasmodium falciparum* D6, W2	Nd
*Triumfetta welwitschii var. hirsuta*	*Malvaceae*	[22]	South Africa	Leaves	Dichloromethane/Methanol	Very Good	3.6 µg/ml IC_50_	*Plasmodium falciparum* D10	Nd
*Turraea floribunda*	*Meliaceae*	[22]	South Africa	Leaves	Dichloromethane/Methanol	Good	8.8 µg/ml IC_50_	*Plasmodium falciparum* D10	Nd
		[26]	Kenya	Stem Barks	Methanol	Good	5.5 µg/ml IC_50_	*Plasmodium falciparum* D6, W2	Nd
*Turraea robusta*	*Meliaceae*	[72]	Kenya	Root Barks	Methanol	Very Good	2.4–3.5 µg/ml IC_50_	*Plasmodium falciparum* K1, NF54	Nd
		[24]	Kenya	Stem Barks	Methanol	Good	2.1–10.3 µg/ml IC_50_	*Plasmodium falciparum* D6, W2	Nd
*Tylosema fassoglensis*	*Fabaceae*	[30]	Kenya	Tubers	Dichloromethane	Very Good	0.77–0.896 µg/ml IC_50_	*Plasmodium falciparum* W2, D6	Nd
*Uapaca paludosa*	*Phyllanthaceae*	[103]	Congo Brazzaville	Barks	Dichloromethane	Good	8 µg/ml IC_50_	*Plasmodium falciparum* Fcm29-Cameroon	Nd
*Uvaria acuminata*	*Annonaceae*	[26]	Kenya	Root Barks	Methanol	Good	6.9–8.9 µg/ml IC_50_	*Plasmodium falciparum* D6, W2	Nd
*Uvaria scheffleri*	*Annonaceae*	[26]	Kenya	Leaves	Methanol	Good	6.8 µg/ml IC_50_	*Plasmodium falciparum* D6, W2	Nd
*Uvaria afzelii*	*Annonaceae*	[48]	Ivory Coast	Roots	Pentane	Moderate	9–22 µg/ml IC_50_	*Plasmodium falciparum* FCM29, CQ-S (Nigerian)	No
*Uvariastrum zenkeri*	*Annonaceae*	[49]	Cameroon	Twigs	Ethanol	Very Good	1.89 µg/ml IC_50_	*Plasmodium falciparum* W2	Nd
*Uvariodendron molundense*	*Annonaceae*	[49]	Cameroon	Twigs	Methanol	Very Good	4.79 µg/ml IC_50_	*Plasmodium falciparum* W2	Nd
*Uvariopsis congolana*	*Annonaceae*	[55]	Cameroon	Stems	Ethanol, Water, Dichloromethane, Methanol, Hexane	Very Good	4.47 µg/ml IC_50_	*Plasmodium falciparum* W2	Nd
*Vangueria infausta Burch. subsp. Infausta*	*Rubiaceae*	[37]	South Africa	Roots	Dichloromethane	Very Good	1.84 µg/ml IC_50_	*Plasmodium falciparum* NF54	Nd
*Vepris lanceolata*	*Rutaceae*	[20]	Kenya	Root Barks	Ethyl Acetate	Good	7.0 µg/ml IC_50_	*Plasmodium falciparum* K1	Nd
*Vernonia amygdalina*	*Asteraceae*	[74]	S. Tome´ And Prı ´Ncipe	Leaves	Ethyl Acetate	Moderate	10 µg/ml IC_50_	*Plasmodium falciparum* 3D7 And Dd2	Nd
		[80]	Cameroon	Leaves	Dichloromethane	Moderate	8.72–11.27 µg/ml IC_50_	*Plasmodium falciparum* 3D7, DD2	No
		[126]	Nigeria	Leaves	Ethanol	Good	9.83 µg/ml IC_50_	*Plasmodium falciparum* 3D7, NF-54	Yes (SI = 6.14; C-1008 kidney fibroblast
		[26]	Kenya	Leaves	Methanol	Good	4.9–7.2 µg/ml IC_50_	*Plasmodium falciparum* D6, W2	Nd
		[127]	Nigeria	Leaves	Ethanol	Moderate	11.2 µg/ml IC_50_	*Plasmodium falciparum*	Yes (LD_50_ = 1950 mg/kg; rat)
		[69]	D.R. Congo	Leaves	Petroleum Ether	Very Good	2.5 µg/ml IC_50_	*Plasmodium falciparum*	Nd
*Vernonia brachycalyx*	*Asteraceae*	[104]	Kenya	Leaves	Dichloromethane/Ethyl Acetate	Good	6.6—8.4 µg/ml IC_50_	*Plasmodium falciparum* K39, V1/S	Nd
*Vernonia cinerea*	*Asteraceae*	[45]	Cambodia	Whole Plant	Dichloromethane	Moderate	18.3 µg/ml IC_50_	*Plasmodium falciparum* W2	Nd
*Vernonia colorata*	*Asteraceae*	[57]	Ivory Coast	Stems, Leaves	Water	Good	2.35–9.38 µg/ml IC_50_	*Plasmodium falciparum* Fcb1 & F32	Nd
		[54]	Zimbabwe	Leaves	Petrolether/Ethylacetate	Moderate	12.1–17.8 µg/ml IC_50_	*Plasmodium falciparum* Pow, Dd2	Nd
		[91]	Comoros	Roots	Dichloromethane	Very Good	3 µg/ml IC_50_	*Plasmodium falciparum* W2	No
		[22]	South Africa	Leaves	Dichloromethane/Methanol	Very Good	4.7 µg/ml IC_50_	*Plasmodium falciparum* D10	Nd
*Vernonia fastigiata*	*Asteraceae*	[22]	South Africa	Leaves	Dichloromethane/Methanol	Moderate	10 µg/ml IC_50_	*Plasmodium falciparum* D10	Nd
*Vernonia guineensis*	*Asteraceae*	[128]	Cameroon	Leaves	Dichloromethane	Very Good	1.635—1.823 µg/ml IC_50_	*Plasmodium falciparum*	No
*Vernonia lasiopus*	*Compositae*	[12]	Kenya	Leaves	Chloroform, Ethylacetate, Methanol	Very Good	1.0–3.2 µg/ml IC_50_	*Plasmodium falciparum* K39 (CQ-S), ENT30, NF54, V1/S	Nd
		[73]	Kenya	Root Barks	Dichloromethane	Very Good	4.7–4.9 µg/ml IC_50_	*Plasmodium falciparum* K1, NF54	Nd
*Vernonia myriantha*	*Asteraceae*	[22]	South Africa	Leaves	Dichloromethane/Methanol	Very Good	3 µg/ml IC_50_	*Plasmodium falciparum* D10	Nd
*Vernonia oligocephala*	*Asteraceae*	[22]	South Africa	Leaves	Dichloromethane/Methanol	Very Good	3.5 µg/ml IC_50_	*Plasmodium falciparum* D10	Nd
*Vismia guineensis*	*Hypericaceae*	[48]	Ivory Coast	Leaves	Pentane	Moderate	15–20 µg/ml IC_50_	*Plasmodium falciparum* FCM29, CQ-S (Nigerian)	Nd
*Warburgia ugandensis*	*Canellaceae*	[72]	Kenya	Stem Barks	Dichloromethane	Very Good	1.4–2.2 µg/ml IC_50_	*Plasmodium falciparum* K1, NF54	Nd
		[24]	Kenya	Root Barks	Methanol	Good	4.1–6.1 µg/ml IC_50_	*Plasmodium falciparum* D6, W2	Nd
*Warburgia stuhlmannii*	*Canellaceae*	[26]	Kenya	Stem Barks	Methanol	Very Good	1.8–2.3 µg/ml IC_50_	*Plasmodium falciparum* D6, W2	Nd
*Ximenia americana*	*Olacaceae*	[57]	Ivory Coast	Stem, Leave	Water	Very Good	0.6–2.6 µg/ml IC_50_	*Plasmodium falciparum* Fcb1 & F32	Nd
*Xylopia aethiopica*	*Annonaceae*	[98]	Cameroon	Stem Barks	Water	Moderate^a^	17.8 µg/ml IC_50_	*Plasmodium falciparum* W5	Nd
		[49]	Cameroon	Leaves	Methanol	Very Good	3.75 µg/ml IC_50_	*Plasmodium falciparum* W2	Nd
*Xylopia africana*	*Annonaceae*	[49]	Cameroon	Stem Barks	Methanol	Very Good	1.07 µg/ml IC_50_	*Plasmodium falciparum* W2	Nd
*Xylopia parviflora (A.Rich.)Benth.Oliv*	*Annonaceae*	[37]	South Africa	Roots	Dichloromethane	Very Good	2.19 µg/ml IC_50_	*Plasmodium falciparum* NF54	Nd
		[49]	Cameroon	Leaves	Methanol	Very Good	3.44 µg/ml IC_50_	*Plasmodium falciparum* W2	Nd
*Xylopia phloiodora*	*Annonaceae*	[98]	Cameroon	Stem Barks	Water	Moderate^a^	17.9 µg/ml IC_50_	*Plasmodium falciparum* W2	Nd
*Xysmalobium undulatum*	*Apocynaceae*	[22]	South Africa	Whole Plant	Dichloromethane/Methanol	Good	6 µg/ml IC_50_	*Plasmodium falciparum* D10	Nd
*Zanthoxylum chalybeum*	*Rutaceae*	[137]	Kenya	Root Barks	Water	Good	2.32–5.52 µg/ml IC_50_	*Plasmodium falciparum* NF54, ENT30	Nd
		[77]	Uganda	Stem Barks	Ethyl Acetate	Very Good	0.57–3.21 µg/ml IC_50_	*Plasmodium falciparum* NF54 & FCR3	Nd
		[92]	Rwanda	Root Barks	Methanol	Very Good	1.9–4.2 µg/ml IC_50_	*Plasmodium falciparum* 3D7, W2	No
		[20]	Tanzania	Root Barks	Ethyl Acetate	Very Good	4.2 µg/ml IC_50_	*Plasmodium falciparum* K1	Nd
		[26]	Kenya	Root Barks	Methanol	Very Good	2.9–3.7 µg/ml IC_50_	*Plasmodium falciparum* D6, W2	Nd
*Zanthoxylum gilletii*	*Rutaceae*	[43]	Ivory Coast	Stem Barks	Ethanol	Very Good	2.8 µg/ml IC_50_	*Plasmodium falciparum* FCB1	Nd
*Zanthoxylum heitzii*	*Rutaceae*	[129]	Republic Of Congo	Barkss	Hexane	Very Good^a^	0.0089 µg/ml IC_50_	*Plasmodium falciparum, Plasmodium berghei*	Nd
*Zanthoxylum tsihanimposa*	*Rutaceae*	[130]	Madagascar	Stem Barks	Dichloromethane + Methanol	Very Good^a^	98.4 µM IC_50_	*Plasmodium falciparum* FCM29	Nd
*Zanthoxylum usambarense*	*Rutaceae*	[24]	Kenya	Root Barks	Methanol	Good	3.2–5.5 µg/ml IC_50_	*Plasmodium falciparum* D6, W2	Nd
Zea mays	*Poaceae*	[131]	Nigeria	Leaves	Ethanol, ethyl acetate	Good	3.69—9.31 µg/ml IC_50_	*Plasmodium falciparum* 3D7, INDO, *Plasmodium berghei*	Nd
*Zehreria scabra*	*Cucurbitaceae*	[22]	South Africa	Whole Plant	Dichloromethane/Methanol	Good	5.6 µg/ml IC_50_	*Plasmodium falciparum* D10	Nd
		[26]	Kenya	Whole Plant	Methanol	Good	9.8 µg/ml IC_50_	*Plasmodium falciparum* D6, W2	Nd
*Ziziphus abyssica*	*Rhamnaceae*	[24]	Kenya	Leaves	Methanol	Moderate	17.5 µg/ml IC_50_	*Plasmodium falciparum* D6, W2	Nd
*Ziziphus mucronata*	*Rhamnaceae*	[22]	South Africa	Leaves	Dichloromethane	Moderate	12 µg/ml IC_50_	*Plasmodium falciparum* D10	Nd
		[25]	South Africa	Stem Barks	Acetone	Very Good	4.13 µg/ml IC_50_	*Plasmodium falciparum* UP1 (CQ-R)	Nd
*Ziziphus cambodiana*	*Rhamnaceae*	[45]	Cambodia	Stems	Dichloromethane	Moderate	19.0 µg/ml IC_50_	*Plasmodium falciparum* W2	Nd

*Nd* Not done, *Ns* Not specified, *SI* Selectivity index

^a^Activity determined using pure compounds isolated from plant

**Table 2:** In vivo antimalarial activity of African medicinal plants

Plant species	*Plant family*	Source	Country of study	Part of plant used	Extraction solvent	Antimalarial activity	Parasite suppression rate	Strain of *Plasmodium* tested	Toxicity (value; assay)
*Acacia nilotica*	*Fabaceae*	[132]	Nigeria	Roots	Water	Moderate	79.5% at 400 mg/kg/day	*Plasmodium berghei* NK65	No
		[133]	Nigeria	Roots	Methanol	Very good	62.59% at 150 mg/kg/day	*Plasmodium berghei* NK65	No
*Adansonia digitata*	*Malvaceae*	[134]	Nigeria	Stem barks	Methanol	Moderate^a^	90.18% at 400 mg/kg/day	*Plasmodium berghei*	Nd
		[135]	Kenya	Stem barks	Ethanol	Very good	> 60% at 100 mg/kg/day	*Plasmodium berghei*	No
		[135]	Kenya	Stem barks	Water	Very good	60.47% at 100 mg/kg/day	*Plasmodium berghei*	No
*Ageratum conyzoides*	*Asteraceae*	[136]	Nigeria	Leaves	Water	Moderate	89.87% at 400 mg/kg/day	*Plasmodium berghei* NK65	Nd
*Albizia gummifera*	*Fabaceae*	[137]	Kenya	Root barks	Methanol	Very good^a^	72.9% at 20 mg/kg.day	*Plasmodium falciparum* NF54 and ENT36	Nd
*Allophylus africanus*	*Sapindaceae*	[138]	Nigeria	Stems, roots	Ns	Very good	92.82–97.81 at 50 mg/kg/day	*Plasmodium berghei* NK-65	Nd
*Aloe pulcherrima*	*Xanthorrhoeaceae*	[139]	Ethiopia	Leaves	Methanol	Good^a^	56.2 at 200 mg/kg/day	*Plasmodium berghei*	No
*Anthocleista djalonensis*	*Gentianaceae*	[140]	Nigeria	Roots	Chloroform, ethyl acetate, methanol	Moderate	64.81–87.66% at 500 mg/kg/day	*Plasmodium beghei* ANKA	No
		[140]	Nigeria	Roots	Ethanol, chloroform, ethyl acetate, methanol	Moderate	67.92% at 500 mg/kg/day	*Plasmodium berghei* ANKA	No
*Artemisia macivarae*	*Asteraceae*	[141]	Nigeria	Whole plant	Chloroform	Very good	80% at 100 mg/kg	*Plasmodium berghei*	Nd
*Aspilia africana*	*Asteraceae*	[142]	Nigeria	Leaves	Ethanol	Moderate	92.23% at 400 mg/kg/day	*Plasmodium berhhei* NK65	No
*Azadirachta indica*	*Meliaceae*	[143]	Kenya	Leaves	Methanol	Good	83.48% at 250 mg/kg/day	*Plasmodium falciparum* D6 and W2	No
		[144]	Cameroon	Leaves	Ethanol	Moderate	69.28% at 300 mg/kg/day	*Plasmodium berghei* NK65	No
		[145]	Nigeria	Leaves	Methanol	Very good	56 – 87% at 50 mg/kg/day	*Plasmodium berghei* ANKA	No
*Balanites rotundifolia*	*Zygophyllaceae*	[146]	Ethiopia	Leaves	Methanol	Moderate	67% at 400 mg/dl	*Plasmodium berghei*	No
*Blighia sapida*	*Sapindaceae*	[147]	Nigeria	Leaves	Ethanol	Good	57% at 200 mg/kg/day	*Plasmodium berghei* ANKA	No
*Bombax buonopozense*	*Malvaceae*	[148]	Nigeria	Root barks	Water	Good	93% at 200 mg/kg/day	*Plasmodium berghei* NK65	Nd
*Brassica nigra*	*Brassicaceae*	[149]	Ethiopia	Seeds	Methanol	Moderate	53.13% at 400 mg/kg/day	*Plasmodium berghei* ANKA	Nd
*Calpurnia aurea*	*Fabaceae*	[150]	Ethiopia	Leaves	Hydroalcohol	Very good	51.15% at 60 mg/kg	*Plasmodium berghei*	No
*Carica papaya*	*Caricaceae*	[151]	Nigeria	Leaves	Ethanol	Good	59.29% at 200 mg/kg	*Plasmodim berghei* NK65	Nd
*Senna occidentalis*	*Fabaceae*	[152]	D.R. Congo	Root barks	Ethanol	Good	68% at 200 mg/kg	*Plasmodium berghei* ANKA	No
*Cassia sieberiana*	*Fabaceae*	[153]	Nigeria	Stems	Ethanol	Good	63.9% at 300 g/kg/day	*Plasmodium berghei* NK65	No
*Cassia singueana*	*Fabaceae*	[154]	Nigeria	Root barks	Methanol	Good	79.06% at 200 mg/kg/day	*Plasmodium berghei*	Yes (LD_50_ = 847 mg/kg; mice)
*Chrozophora senegalensis*	*Euphorbiaceae*	[155]	Nigeria	Whole plant	Methanol	Very good	51.8% at 75 mg/kg/day	*Plasmodium berghei*	Nd
*Chrysophyllum albidum*	*Sapotaceae*	[156]	Nigeria	Seeds, pulp juice	Ethanol	Moderate	72.97% at 500 mg/kg	*Plasmodium berghei*	No
*Clausena anisota*	*Rutaceae*	[157]	Nigeria	Leaves	Ethanol	Very good	82.02% at 78 mg/kg/day	*Plasmodium berghei*	Yes (LD_50_ = 393.7 mg/kg; albino mice)
*Combretum molle*	*Combretaceae*	[158]	Ethiopia	Seeds	Methanol	Good	63.5% at 250 mg/kg/day	*Plasmodium berghei* ANKA	Nd
*Commiphora africana*	*Burseraceae*	[159]	Tanzania	Stem barks	Dichloromethane	Moderate	64.24% at 400 mg/kg/day	*Plasmodium falciparum* (D6, Dd2), *Plasmodium berghei*	No
*Crossopteryx febrifuga*	*Rubiaceae*	[160]	Nigeria	Stem barks	Ethanol	Good	63.65% at 200 mg/kg/day	*Plasmodium berghei* var. ANKA	Nd
*Croton macrostachyus*	*Euphorbiaceae*	[161]	Kenya	Stem barks	Ethyl acetate	Moderate	82% at 500 mg/kg/day	*Plasmodium berghei* ANKA	Nd
*Cryptolepis sanguinolenta*	*Apocynaceae*	[162]	Congo	Root barks	Ethanol	Moderate	75.07% at 400 mg/kg/day	*Plasmodium falciparum, Plasmodium berghei berghei*	Nd
		[84]	Ghana	Roots	Hexane, ethanol, dichloromethane	Very good*	> 80% at 2.5 mg/kg/day	*Plasmodium vinckei petteri, Plasmodium berghei* ANKA	Nd
*Cucumis metuliferus*	*Cucurbitaceae*	[163]	Tanzania	Leaves	Chloroform	Moderate	70.69% at 600 mg/kg/day	*Plasmodium berghei* ANKA	Nd
*Dichrostachys cinerea*	*Fabaceae*	[159]	Tanzania	Stem barks	Methanol	Moderate	53.12% at 400 mg/kg/day	*Plasmodium falciparum* (D6, Dd2), *Plasmodium berghei*	No
*Dodonaea angustifolia*	*Sapindaceae*	[164]	Ethiopia	Roots	N-butanol	Moderate	55.8% at 400 mg/kg/day	*Plasmodium berghei*	Nd
*Enantia chlorantha Oliv*	*Annonaceae*	[165]	Nigeria	Stem barks	Ethanol	Moderate	75.23% at 500 mg/kg	*Plasmodium berghei* NK-65	Nd
*Erigeron floribundus*	*Asteraceae*	[144]	Cameroon	Whole plant	Ethanol	Good	62.4% at 240 mg/kg/day	*Plasmodium berghei* NK65	No
*Euphorbia cordifolia*	*Euphorbiaceae*	[166]	Cameroon	Whole plant	Aqueous	Very good	94.70% at 200 mg/kg/day	*Plasmodium berghei*	No
*Euphorbia hirta L*	*Euphorbiaceae*	[162]	Congo	Whole plant	Ethanol	Moderate	69.44% at 400 mg/kg/day	*Plasmodium falciparum, Plasmodium berghei berghei*	Nd
*Faidherbia albida*	*Fabaceae*	[167]	Nigeria	Stem barks	Ethanol	Moderate	89.5 at 400 mg/kg/day	*Plasmodium berghei* NK65	Nd
*Grewia plagiophylla*	*Malvaceae*	[143]	Kenya	Leaves	Methanol	Moderate	77.9 at 250 mg/kg/day	*Plasmodium falciparum* D6 and W2	Nd
*Grewia trichocarpa*	*Malvaceae*	[168]	Kenya	Root	Water	Good	35.8% at 10 mg/kg/day	*Plasmodium berghei*	Yes (LD_50_ = 545.8 µg/ml; brine shrimp)
*Garcinia kola*	*Clusiaceae*	[169]	Nigeria	Seeds	Petroleum ether	Very good*	93% at 200 mg/kg/day	*Plasmodium berghei*	Nd
*Hippocratea africana*	*Celastraceae*	[170]	Nigeria	Nd	Ethanol	Moderate	90.9% at 600 mg/kg/day	*Plasmodium berghei berghei*	Yes (LD_50_ = 2449 mg/kg; mice)
*Hoslundia opposita*	*Lamiaceae*	[143]	Kenya	Leaves	Methanol	Moderate	79.67% at 250 mg/kg/day	*Plasmodium falciparum* D6 and W2	Yes (CC_50_ = 37 µg/ml; Vero E6 cells)
*Icacina senegalensis*	*Icacinaceae*	[171]	Nigeria	Leaves	Methanol	Very good	80% at 100 mg/kg/day	*Plasmodium berghei*	Yes (LD_50_ > 2000 mg/kg; mice)
*Indigofera spicata*	*Fabaceae*	[172]	Ethiopia	Roots	Methanol	Moderate	53.42% at 600 mg/kg/day	*Plasmodium berghei* ANKA	Nd
*Lannea schweinfurthii*	*Anacardiaceae*	[143]	Kenya	Leaves	Methanol	Moderate	83.48% at 250 mg/kg/day	*Plasmodium falciparum* D6 and W2	Yes (CC_50_ = 76 µg/ml; Vero E6 cells)
*Lippia kituiensis*	*Verbenaceae*	[163]	Tanzania	Leaves	Ethyl acetate	Moderate	70.14% at 600 mg/kg/day	*Plasmodium berghei* ANKA	Nd
*Lophira lanceolata*	*Ochnaceae*	[173]	Nigeria	Leaves	Methanol	Moderate	80% at 400 mg/kg/day	*Plasmodium berghei*	No
*Maerua crassifolia*	*Capparaceae*	[174]	Nigeria	Leaves	Methanol	Moderate	86% at 400 mg/kg/day	*Plasmodium berghei* NK65	No
*Maytenus senegalensis*	*Celastraceae*	[175]	Tanzania	Root barks	Ethanol	Very good	98.1% at 100 mg/kg/day	*Plasmodium berghei*	No
*Morinda morindoides*	*Rubiaceae*	[152]	D.R. Congo	Leaves	Dichloromethane	Good	74% at 200 mg/kg/day	*Plasmodium berghei* ANKA	No
*Mucuna pruriens*	*Fabaceae*	[176]	Nigeria	Leaves	Water	Good	71.75% at 270 mg/kg/day	*Plasmodium berghei* NK65	No
*Nauclea latifolia*	*Rubiaceae*	[177]	Nigeria	Leaves	Ethanol	Moderate	60.63% at 500 mg/kg/day	*Plasmodium berghei*	No
		[165]	Nigeria	Roots	Ethanol	Moderate	71.15% at 500 mg/kg/day	*Plasmodium berghei* NK-65	Nd
*Oldenlandia affinis*	*Rubiaceae*	[178]	Nigeria	Aerial parts	Methanol, water, dichloromethane	Moderate	75% at 400 mg/kg/day	*Plasmodium berghei*	No
*Peschiera fuchsiaefolia*	*Apocynaceae*	[179]	Madagascar	Stem barks	Ns	Good*	43.4% at 10 mg/kg/day	*Plasmodium yoelii* N67, *Plasmodium falciparum* FMC29	Nd
*Phyllanthus amarus*	*Phyllanthaceae*	[180]	Nigeria	Whole plant	Water and ethanol	Good	79% at 1600 mg/kg/day	*Plasmodium yoelii*	Nd
*Phyllanthus niruri*	*Phyllanthaceae*	[152]	D.R. Congo	Whole plant	Ethanol	Good	73% at 200 mg/kg/day	*Plasmodium berghei* ANKA	No
		[181]	Nigeria	Aerial parts	Methanol/chloroform	Very good	90.48% at 100 mg/kg/day	*Plasmodium berghei berghei NK 65*	Nd
*Phytolacca dodecandra*	*Phytolaccaceae*	[182]	Ethiopia	Leaves	Methanol	Moderate	55.24% at 400 mg/kg/day	*Plasmodium berghei*	Nd
*Picralima nitida*	*Apocynaceae*	[183]	Nigeria	Seeds	Ethanol	Good	73% at 115 mg/kg/day	*Plasmodium berghei berghei*	Yes (LD_50_ = 87.29 µg/ml; albino mice)
*Piliostigma thonningii*	*Fabaceae*	[184]	Nigeria	Leaves	Ethanol	Moderate	91% at 400 mg/kg/day	*Plasmodium berghei* NK65	No
*Premna chrysoclada*	*Lamiaceae*	[143]	Kenya	Leaves	Methanol	Good	65.08% at 250 mg/kg/day	*Plasmodium falciparum* D6 and W2	Nd
*Pseudocedrela kotschyi*	*Meliaceae*	[185]	Nigeria	Leaves	Ethanol	Moderate	90% at 400 mg/kg/day	*Plasmodium berghei* (NK65	No,
*Rhus natalensis*	*Anacardiaceae*	[143]	Kenya	Leaves	Methanol	Moderate	82.7% at 250 mg/kg/day	*Plasmodium falciparum* D6 and W2	Nd
*Salacia nitida*	*Celastraceae*	[165]	Nigeria	Roots	Ethanol	Moderate	71.15% at 250 mg/kg/day	*Plasmodium berghei* NK-65	Nd
*Stachytarpheta cayennensis*	*Verbenaceae*	[186]	Nigeria	Leaves	Ethanol	Good	78.2% at 270 mg/kg/day	*Plasmodium berghei berghei*	Yes (LD_50_ = 938.08 mg/kg; albino mice)
*Telfairia occidentalis*	*Cucurbitaceae*	[187]	Nigeria	Leaves	Water	Good	72.17% at 200 mg/kg/day	*Plasmodium berghei* ANKA	No
*Tithonia diversifolia*	*Asteraceae*	[160]	Nigeria	Aerial parts	Ethanol	Good	74.97% at 200 mg/kg/day	*Plasmodium berghei* var. ANKA I	Nd
*Toddalia asiatica*	*Rutaceae*	[188]	Kenya	Root barks	Methanol	Moderate	59.3% at 500 mg/kg/day	*Plasmodium berghei* NK66	Nd
*Trema orientalis*	*Cannabaceae*	[189]	Nigeria	Stem barks	Methanol	Good	70% at 200 mg/kg/day	*Plasmodium berghei*	Nd
*Trichilia megalantha*	*Meliaceae*	[190]	Nigeria	Stem barks	Methanol, chloroform	Good	89.1–100% at 200 mg/kg/day	*Plasmodium berghei berghei* ANKA	Nd
*Triphyophyllum peltatum*	*Dioncophyllaceae*	[191]	Ivory Coast	Roots, stem barks	Dichloromethane	Very good*	99% at 50 mg/kg/day	*Plasmodium berghei* ANKA CRS	Nd
*Uvaria acuminata*	*Annonaceae*	[143]	Kenya	Roots	Methanol	Good	27.0% at 250 mg/kg/day	*Plasmodium falciparum* D6 and W2	Nd
*Uvaria chamae P. Beauv*	*Annonaceae*	[170]	Nigeria	Nd	Ethanol	Moderate	72.2% at 600 mg/kg/day	*Plasmodium berghei berghei*	Yes (LD_50_ = 3464 mg/kg; mice)
*Verbena hastata*	*Verbenaceae*	[192]	Nigeria	Leaves	Ethanol	Moderate	70% at 400 mg/kg/day	*Plasmodium berghei*	No
*Vernonia amygdalina*	*Asteraceae*	[193]	Uganda	Leaves	Water	Good	73% at 200 mg/kg/day	*Plasmodium berghei*	No
		[194]	Nigeria	Leaves	Water	Good	50.78—62.66% at 125 mg/kg/day	*Plasmodium berghei* ANKA	Nd
		[195]	Botswana	Leaves and root barks	Ethanol	Moderate	67% at 500 mg/kg/day	*Plasmodium berghei*	Nd
*Vernonia lasiopus*	*Asteraceae*	[188]	Kenya	Root barks	Methanol	Moderate	59.3% at 500 mg/kg/day	*Plasmodium berghei* NK67	Nd
*Withania somnifera*	*Solanaceae*	[196]	Ethiopia	Leaves	Methanol	Moderate	57% at 300 mg/kg/day	*Plasmodium berghei* ANKA	Nd
*Xylopia aethiopica*	*Annonaceae*	[141]	Nigeria	Fruits	Chloroform	Very good	60% at 100 mg/kg/day	*Plasmodium berghei*	Nd
*Artemisia abyssinica*	*Asteraceae*	[197]	Ethiopia	Aerial parts	Hydroalcohol	Good	64.7% at 200 mg/kg/day	*Plasmodium berghei*	Nd
*Rotheca myricoides*	*Lamiaceae*	[198]	Ethiopia	Leaves	Methanol	Good	54.14% at 200 mg/kg/day	*Plasmodium berghei*	No
*Dodonaea angustifolia*	*Sapindaceae*	[198]	Ethiopia	Roots	Methanol	Good	57.74% at 200 mg/kg/day	*Plasmodium berghei*	No
*Clutia abyssinica*	*Peraceae*	[199]	Kenya	Leaves	Methanol	Moderate	40.45% at 100 mg/kg/day	*Plasmodium falciparum, Plasmodium berghei* ANKA	No
*Pittosporum viridiflorum*	*Pittosporaceae*	[199]	Kenya	Leaves	Methanol	Moderate	54.77% at 100 mg/kg/day	*Plasmodium falciparum* D6 &W2, *Plasmodium berghei* ANKA	Yes (SI = 2.51; Vero E6 cells)

*Nd* Not done, *Ns* Not specified, *SI* Selectivity index

^a^Activity determined using pure compounds isolated from plant

**Table 3:** In vitro and in vivo studies on African medicinal plants

Plant species	Plant family	Source	Country of study	Part of plant used	Extraction solvent	Overall activity	In vitro	In vivo	IC_50_ or ED_50_ or LD_50_	Strain of *Plasmodium* tested	parasite suppression rate	Toxicity (value; assay)
*Sphaeranthus suaveolens*	*Compositae*	[199]	Kenya	Whole plant	Methanol	Moderate	Moderate	In active	7.93–56.73 µg/ml IC_50_	*Plasmodium falciparum* D6 and W2, *Plasmodium berghei* ANKA	46.74% at 100 mg/kg/day	No
*Abutilon grandiflorum*	*Malvaceae*	[200]	Tanzania	Roots	Ethyl acetate	Good	Moderate	Very good	9–14 µg/mL IC_50_	*Plasmodium falciparum* HB3 and FCB, *Plasmodium vinckei vinckei*	83–87% at 20 ug/ml/day	Yes (IC_50_ = 36 µg/ml; human colon carcinoma cell line [HT29])
*Alchornea laxiflora*	*Euphorbiaceae*	[131]	Nigeria	Roots	Ethyl acetate, dichloromethane	Good	Inactive	Very good	38.44—40.17 µg/ml IC_50_	*Plasmodium falciparum* 3Dè, INDO, *Plasmodium berghei*	65.73% at 150 mg/kg/day	Yes (LD_50_ = 748.33 mg/kg; HeLa cells)
*Annona senegalensis*	*Annonaceae*	[201]	Nigeria	Leaves	Methanol	Moderate	In active	Very good	28.8 µg/ml IC_50_	*Plasmodium berghei*	> 57% at 100 mg/kg/day	No
*Boscia angustifolia*	*Capparaceae*	[199]	Kenya	Stem barks	Methanol	Moderate	Moderate	Very good	7.43–35.93 µg/ml IC_50_	*Plasmodium falciparum* D6 &W2, *Plasmodium berghei* ANKA	60.12% at 100 mg/kg/day	No
*Chrozophora senegalensis*	*Euphorbiaceae*	[64]	Senegal	Leaves	Water	Very good	Very good	Very good	1.6–1.9 µg/ml IC_50_	*Plasmodium falciparum* FcM29, FcB1, *Plasmodium vinckei petteri*	65% at 10 mg/kg/day	No
*Clerodendrum eriophyllum*	*Lamiaceae*	[199]	Kenya	Root barks	Methanol	Moderate	Good	Very good	9.51–10.56 µg/ml IC_50_	*Plasmodium falciparum* D6 & W2, *Plasmodium berghei* ANKA	90.13% at 100 mg/kg/day	No
*Cocos nucifera*	*Arecaceae*	[202]	Nigeria	Husk	Ethyl acetate	Moderate	Moderate	Very good	10.94 µg/ml IC_50_	*Plasmodium falciparum* W2, *Plasmodium berghe*i NK65	98.6% at 125 mg/kg/day	Nd
*Commiphora africana*	*Burseraceae*	[159]	Tanzania	Stem barks	Dichloromethane	Moderate	Very good	Moderate	4.54 µg/ml IC_50_	*Plasmodium falciparum* D6, Dd2, *Plasmodium berghei*	64.24% at 400 mg/kg/day	No
*Ficus thonningii*	*Moraceae*	[203]	Nigeria	Whole plant	Hexane	Moderate	Good	Moderate	2.7–10.4 µg/ml IC_50_	*Plasmodium falciparum* NF54, K1, *Plasmodium berghei* NK65	84.5% at 500 mg/kg/day	No
*Flueggea virosa*	*Phyllanthaceae*	[199]	Kenya	Leaves	Methanol	Very good	Very good	Very good	2.28–3.64 µg/ml IC_50_	*Plasmodium falciparum* D6 and W2, *Plasmodium berghei* ANKA	70.91% at 100 mg/kg/day	No
*Fuerstia africana*	*Lamiaceae*	[199]	Kenya	Whole plant	Methanol	Very good	Very good	Very good	0.98–2.40 µg//ml IC_50_	*Plasmodium falciparum* D6 and W2, *Plasmodium berghei* ANKA	61.85% at 100 mg/kg/day	No
*Harungana madagascariensis*	*Hypericaceae*	[199]	Kenya	Leaves	Water	Moderate	Inactive	Very good	39.07–43.7 µg/ml IC_50_	*Plasmodium falciparum* D6 and W2, *Plasmodium berghei* ANKA	88.04% at 100 mg/kg/day	No
		[204]	Nigeria	Stem barks	Ethanol	Very good	Very good	Inactive	0.052—0.517 μg/ml IC_50_	*Plasmodium yoelii* nigeriensis N67, *Plasmodium falciparum*	28.6–44.8%	Nd
*Lannea schweinfurthii*	*Anacardiaceae*	[205]	Kenya	Stem barks	Methanol	Moderate	Moderate	Very good	11.38–36.26 µg/ml IC_50_	*Plasmodium falciparum* D6, W2, *Plasmodium berghei*	91.37% at 100 mg/kg/day	Yes (SI = 6.21–19.79; Vero cells)
*Lophira alata*	*Ochnaceae*	[203]	Nigeria	Whole plant	Hexane	Good	Very good	Moderate	2.5 µg/ml IC_50_	*Plasmodium falciparum* NF54, K1, *Plasmodium berghei* NK65	74.45% at 500 mg/kg/day	No
*Ludwigia erecta*	*Onagraceae*	[199]	Kenya	Whole plant	Water	Very good	Very good	In active	0.93–1.61 µg/ml IC_50_	*Plasmodium falciparum* D6 & W2, *Plasmodium berghei* ANKA	49.64% at 100 mg/kg/day	No
*Maytenus putterlickioides*	*Celastraceae*	[199]	Kenya	Root barks	Methanol	Good	Good	Very good	4.41–10.26 µg/ml IC_50_	*Plasmodium falciparum* D6 and W2, *Plasmodium berghei* ANKA	78.66% at 100 mg/kg/day	No
*Maytenus undata*	*Celastraceae*	[199]	Kenya	Leaves	Methanol	Good	Good	Very good	7.4–9.89 µg/ml IC_50_	*Plasmodium falciparum* D6 and W2, *Plasmodium berghei* ANKA	76.29% at 100 mg/kg/day	No
*Mimusops caffra*	*Sapotaceae*	[206]	South Africa	Leaves	Dichloromethane	Good	Very good	Moderate	2.14 µg/ml IC_50_	*Plasmodium falciparum* D10, *Plasmodium berghei*	94.01% at 400 mg/kg/day	Nd
*Schkuhria pinnata*	*Compositae*	[199]	Kenya	Whole plant	Methanol	Good	Good	In actice	1.3–6.83 µg/ml IC_50_	*Plasmodium falciparum* D6 & W2, *Plasmodium berghei* ANKA	49.9% at 100 mg/kg/day	No
*Sclerocarya birrea*	*Anacardiaceae*	[205]	Kenya	Stem barks	Methanol	Moderate	Moderate	Very good	5.91–24.96 µg/ml IC_50_	*Plasmodium falciparum* D6, W2*, Plasmodium berghei*	63.49% at 100 mg/kg/day	No
*Toddalia asiatica*	*Rutaceae*	[117]	Kenya	Fruits	Ethyl acetate	Very good	Very good	Moderate	1.87 μg/ml IC_50_	*Plasmodium falciparum* W2 & D6, *Plasmodium berghei*	81.34% at 500 mg/kg/day	No
*Turraea robusta*	*Meliaceae*	[205]	Kenya	Root barks	Methanol	Good	Good	Very good	2.09–10.32 µg/ml IC_50_	*Plasmodium falciparum* D6, W2, *Plasmodium berghei*	78.2% at 100 mg/kg/day	Yes (SI = 2.36–11.67; Vero cells)
*Uapaca nitida*	*Phyllanthaceae*	[207]	Tanzania	Root barks	Ethanol	Moderate*	Inactive	Inactive	19.6—25.9 µg/mL IC_50_	*Plasmodium falciparum* K1, T9-96 & *Plasmodium berghei*	poor	No
*Vernonia ambigua*	*Asteraceae*	[208]	Nigeria	Ns	Water	Very good	Inactive	Very good	31.26–50 µg/ml IC_50_	*Plasmodium berghei, Plasmodium falciparum*	60% at 100 mg/kg/day	No
		[209]	Republic of Congo	Leaves	Methanol	Moderate	Very good	Moderate	3.58 µg/ml IC_50_	*Plasmodium falciparum. Plasmodium yoelii*	61.28% at 500 mg/kg/day	No
*Warburgia stuhlmannii*	*Camellaceae*	[199]	Kenya	Stem barks	Water	Very good	Very good	Very good	1.81–2.33 µg/ml IC_50_	*Plasmodium falciparum* D6 and W2, *Plasmodium berghei* ANKA	84.95% at 100 mg/kg/day	No
*Azadirachta indica*	*Meliaceae*	[143]	Kenya	Leaves	Methanol	Good	Good	Good	6.24–7.53 µg/ml IC_50_	*Plasmodium falciparum* D6 and W2	83.48% at 250 mg/kg/day	No
*Dichrostachys cinerea*	*Fabaceae*	[159]	Tanzania	Stem barks	Methanol	Moderate	Good	Moderate	2.37–11.92 µg/ml IC_50_	*Plasmodium falciparum* D6, Dd2, *Plasmodium berghei*	53.12% at 400 mg/kg/day	No
*Grewia plagiophylla*	*Malvaceae*	[143]	Kenya	Leaves	Methanol	Moderate	Moderate	Good	13.28–34.2 µg/ml IC_50_	*Plasmodium falciparum* D6 and W2	77.9% at 250 mg/kg/day	Nd
*Hoslundia opposita*	*Lamiaceae*	[143]	Kenya	Leaves	Methanol	Moderate	Good	Good	12.8–13.22 µg/ml IC_50_	*Plasmodium falciparum* D6 and W2	79.67% at 250 mg/kg/day	Yes (SI = 0.58; Vero E6 cells)
*Lannea schweinfurthii*	*Anacardiaceae*	[143]	Kenya	Leaves	Methanol	Moderate	Inactive	Good	38.87–54.15 µg/ml IC_50_	*Plasmodium falciparum* D6 and W2	83.48% at 250 mg/kg/day	Yes (SI = 1.4; Vero E6 cells)
*Premna chrysoclada*	*Lamiaceae*	[143]	Kenya	Leaves	Methanol	Good	Good	Good	7.75–9.02 µg/ml IC_50_	*Plasmodium falciparum* D6 and W2	65.08% at 250 mg/kg/day	Nd
*Rhus natalensis*	*Anacardiaceae*	[143]	Kenya	Leaves	Methanol	Moderate	Inactive	Good	43.93–51.2 µg/ml IC_50_	*Plasmodium falciparum* D6 and W2	82.7% at 250 mg/kg/day	Nd
*Triphyophyllum peltatum*	*Dioncophyllaceae*	[191]	Ivory coast	Roots, stem barks	Dichloromethane	Very good*	Very good	Very good	1.90 mg/kg for Dioncophylline C and 10.71 mg/kg for dioncophylline A	*Plasmodium berghei* ANKA CRS	99% at 50 mg/kg/day	Nd
*Uvaria acuminata*	*Annonaceae*	[143]	Kenya	Roots	Methanol	Good	Good	In active	6.90–8.89 µg/ml IC_50_	*Plasmodium falciparum* D6 and W2	27.0% at 250 mg/kg/day	Nd

*Nd* Not done, *Ns* Not specified, *SI* Selectivity index

^a^Activity determined using pure compounds isolated from plant

**Table 4:** Clinical trial on African medicinal plants

Plant species	Plant family	Source	Country of study	Part of plant used	Extraction solvent	Crude extract?	Antimalarial activity	Parasite suppression rate	Strain of Plasmodium tested	Toxicity
*Cochlospermum planchoni*i	Bixaceae	[210]	Burkina Faso	Roots	Ns	Yes	Moderate	52 at 600 ml/day	*Plasmodium falciparum*	No

*Nd* Not done, *Ns* Not specified

### Family and species distribution of plants evaluated

From 722 studies, the most frequent plant families studied included *Fabaceae* 47 (6.5%), *Euphorbiaceae* 45 (6.2%), *Annonaceae* 37 (5.1%), *Rubiaceae* 37 (5.1%), *Rutaceae* 37 (5.1%), *Meliaceae* 30 (4.2%), and *Lamiaceae* 12 (1.7%). Five hundred and two (502) plant species were investigated in this study. Of them, the most investigated were: *Azadirachta indica*, *Zanthoxylum chalybeum*, *Picrilima nitida*, and *Nauclea latifolia*. The most frequent parts of the plants tested were the leaves, roots, root barkss, stems, and the whole plant. A majority of the studies used the crude extracts of the plants compared to pure compounds (95.7% *vs.* 4.3%). In descending order, methanol 322 (44.7%), dichloromethane 207 (28.7%), ethanol 103 (14.3%), water 85 (11.7%) and ethyl acetate 62 (8.6%) were the most frequent extraction solvent used.

### In vitro and in vivo activities of the plants evaluated

Overall, 248 (34.3%) of the studies reported activity that was very good (IC_50_ values < 5 µg/ml or suppression rate of ≥ 50% at 100 mg/kg body weight/day), 241 (33.4%) reported good activity and 233 (32.3%) reported moderate activity. For the in vitro studies, a majority 228 (38.6%) reported very good activity; 206 (34.9%) reported good activity and 187 (31.6%) reported moderate activity. Meanwhile for the in vivo studies, a majority 19 (21.1%) reported moderate activity, 16 (17.8%) reported very good activity and 13 (14.4%) reported good activity. For studies reporting both the in vitro and in vivo activity, a majority of 17 (42.5%) reported only moderate activity, 13 (32.5%) studies reported very good activity and 10 (25.0%) reported good activity. Among the plants with very good activity, only one species demonstrated very good activity both in vitro and in vivo (Table 3).

Among the studies, the most frequent plant species demonstrating very good antiplasmodial activity were: *Alchornea cordifolia* [3/3, 100%], *Flueggea virosa* [3/3, 100%], *Cryptolepis sanguinolenta* [¾, 75%], *Zanthoxylum chalbeum* [4/5, 80%] and *Maytenus senegalensis* [3/6, 50%]. Plant families with the most active species include *Rutaceae* [13/25, 52.0%], *Apocynaceae* [13/26, 50%], *Celastraceae* [7/15, 46.7%], *Annonaceae* [17/37, 45.9%], *Euphorbiaceae* [21/48, 43.8], *Combretaceae* [7/16, 43.8%], *Fabaceae* [18/47, 38.3%], *Lamiaceae* [8/23, 34.8%], *Asteraceae* [23/69, 33.3%], and *Rubiaceae* [8/37, 21.6%]. The fractions are derived from the count of studies reporting very good antiplasmodial activity (numerator) divided by the total number of studies that assessed the activity of that plant species (denominator).

*Azadirachta indica* and *Vernonia amygdalina* were the most frequently reported inactive species (Additional file 1: Table S1). Furthermore, *Fabaceae, Rubiaceae, Euphorbiaceae*, and *Asteraceae* were the plant families containing the most frequently reported inactive plants. A majority of 95.7% (691/722) of the studies used the crude extract of the plants. The antiplasmodial and/or anti-malarial activity was significantly higher (p = 0.044) in studies using pure compounds compared to those using crude preparations.

### Toxicity of plants evaluated for their antiplasmodial and anti-malarial activity

Out of the 198 plants evaluated in toxicity assays, 52 (26.3%) were found to demonstrate some degree of toxicity. The most frequently reported plants with toxicity were *Azadirachta indica* and *Vernonia amygdalina.* Plant families harboring the most toxic species were *Lamiaceae, Anacardiaceae, Moraceae, Meliaceae, Asteraceae,* and *Fabaceae*. Approximately 33% of the plants tested demonstrated some toxicity in vitro and 26.7% had some degree of toxicity in vivo. Among plants with very good, good, and moderate antiplasmodial activity, 17.8%, 28.3%, and 35.4% had some degree of toxicity, respectively. The leaf was the plant part with the most frequently reported toxicity. Albino mice and Vero E6 cells were the most commonly used assays for the assessment of the toxicity of the plants.

## Discussion

Resistance to the frontline anti-malarial drugs is increasing and is now a global concern. With this rising rate of resistance, there is a need to accelerate research into the discovery and development of new anti-malarial drugs. Unfortunately, from this study, it is evident that the progress into the discovery of a new anti-malarial drug in Africa is slothful. Despite a considerable number of plant species that have demonstrated significant antiplasmodial activity in vitro, fewer plants have been evaluated in vivo and only one clinical trial with *Cochlospermum planchonii* (*Bixaceae*) has been conducted so far. This reinforces the need for basic and clinical research in the region. Van Wyk [213] had also arrived at the same conclusion.

This review revealed research articles from 31 African countries. Most of the articles were from Nigeria. This is suggestive that Nigeria is leading the podium in research on anti-malarial drug discovery and development, deservedly so, because she is probably the most affected country in the world. It is noteworthy that South Africa which is generally more technologically advanced than Nigeria had very few (8) articles. The African region is the most affected in the world recording the greatest number of cases and malaria attributed deaths. However, the distribution of malaria in Africa is not even, with sub-Saharan Africa harboring disproportionately the greatest number of cases. This is suggestive that research to identify new anti-malarial drugs may be related to the burden of the disease, thus the government policy to control the disease. There is, therefore, the need for policy-driven research into new anti-malarial all across the African region. In this review, IC_50_ values of < 20 µg/ml were considered as the cutoff of significant anti-malarial activity. This cutoff is considered the minimum to qualify as a first-pass “hit” in anti-malarial drugs screening [214]. Five hundred and two (502) plant species from 169 families were observed to have moderate to very good anti-malarial activity. The most investigated plant families were *Euphorbiaceae, Fabaceae, Rubiaceae,* and *Annonaceae*. However, the plant families containing the most active plants were *Apocynaceae, Celestraceae,* and *Rutaceae*. This finding suggests that more emphasis should be given to plants in these families for anti-malarial drug discovery. Besides, the most investigated plant species were *Azadirachta indica, Nauclea latifolia, Picralima nitida,* and *Zanthoxylum chalybeum*. *Alchornea cordifolia, Flueggea virosa, Crytolepis sanguinolenta,* and *Zanthoxylum chalybeum* were the only plant species with consistently very good antiplasmodial and anti-malarial activities between studies. This is very surprising that no clinical trial using any of these plants has been conducted. Further studies on these plant species should be performed.

This study revealed that overall, a majority of the plants investigated had very good antiplasmodial activity in vitro. That activity decreases as you move to in vivo in most studies, with a majority of plants demonstrating only moderate activity. For example, Gathirwa et al. [146] showed that the activity of *Uvaria acuminate* decreased from good activity in vitro to inactive in vivo. However, a few studies show that plant activity could also increase from in vitro to in vivo. For example, Ngbolua et al. [211] showed that the activity of *Vernonia ambigua* increased from in vitro to in vivo analysis. Other examples include studies by Muthaura et al. [20] using *Boscia angustifolia*, Kweyamba et al. [162] using *Commiphora Africana,* and Ajaiyeoba et al. [204] using *Annona senegalensis*. This suggests that plants could still have significant anti-malarial activity in vivo although they failed to in vitro. Most investigators usually progress to in vivo studies only when they observe significant antiplasmodial activity in vitro. This may explain the findings of a smaller number of in vivo studies in the current study. The investigation of the anti-malarial activities of plants should continue in vivo despite the dismal performance of the plants in vitro.

The current study revealed substantial inter-study variation in the antiplasmodial activity of several plant species. For example, considerable variation in the antiplasmodial activity was observed for *Senna occidentalis, Adansonia digitata, Acanthospermum hispidum, Rotheca myricoides, Anogeissus leocarpus, Annona muricata, Ageratum conyzoides, Albizia coriaria, Ekebergia capensis, Flueggea virosa, Lippia javanica, Maytenus senegalensis, Morinda lucida, Picralima nitida, Trichilia emetica, Vernonia amydalina,* and *Vernonia colorata*. The factors that could have accounted for these differences may include differences in the extraction solvent thus the extraction yield and extracted metabolite. With dichloromethane, mainly the apolar metabolites are extracted. In contrast, with methanol, from polar to moderate apolar metabolites are extracted.

Most (95.7%) of the studies used crude extract for their investigation and rarely the pure compounds (Additional file 1: Table S2 presents a summary of active compounds that have been identified from some of the plants). The finding of a majority of studies in Africa using only the crude extract of plants may be attributed to the absence of the necessary infrastructure to process the plant materials to get the pure compounds. Furthermore, there may be geographical differences in the areas where the plants were collected and this may also affect the activity of the same plant species. For example, despite using the same extraction solvent, the antiplasmodial activity of *Acacia nilotica* was moderate in South Africa and very good in Sudan. There was also variation between the different assay types. For example, the activities of *Vernonia ambigua* [211] and *Annona senegalensis* [204] have been reported to increase from inactive in vitro to very good in vivo. However, a few plant species including *Alchornea cordifolia,* and *Zanthoxylum chalybeum*, were observed to be consistently very good between studies. These plant species should be exploited further for their antiplasmodial activity. The activities of the plants were equally observed to increase with the isolation of the active compounds thus reinforcing the need for research into identifying the active compounds of African medicinal plants. The marked difference in the antiplasmodial activity of the crude extract of *Artemisia annua* and the pure compounds points out the issue that even the compounds which show only low potency and may be discarded from the initial screen for further development may still have active components with therapeutic potential [215]. The strain of the *Plasmodium* used may also be another factor accounting for the inter-study variation observed; studies using chloroquine-sensitive strains of the parasite like *P. falciparum* 3D7, D6, NF54 tend to report higher antiplasmodial activity compared to studies using chloroquine-resistant strains like *P. falciparum* W2, Dd5, K1 or D10.

This study revealed that only a few (26.3%) of the plants demonstrated some degree of toxicity. The families hosting the most toxic plant species were *Lamiaceae, Anacardiaceae, Moraceae,* and *Meliaceae*. The most toxic plants were *Azadirachta indica* and *Vernonia amygdalina*. The former [168] is one of the few plant species that demonstrated very good antiplasmodial activity in some studies. Other plants with high toxicity but very good antiplasmodial/anti-malarial activities include *Arenga engleri* [25], *Celtis integrifolia* [52], *Ficus platyhylla* [50], *Gutenbergia cordifolia* [21], *Helchrysum cymosum* [97], *Microglossa pyrifolia* [92], *Opilia celtidifolia* [52], *Quassia Africana* [103], *Rumex abyssinicus* [92], *Clausena anisota* [157], *Icacina senegalensis* [171], *Abutilon grandiflorum* [200], and *Lannea schweinfurthii* [205]. The isolation of the active compounds, which has to be done, could eliminate the toxicity, if not all, to a certain degree. For example, *Salvia radula* crude extract (of aerial parts) has been shown to demonstrate some degree of toxicity, but betulafolientriol oxide isolated from the plant was very active with little or no toxicity against human kidney epithelial cells [120]. There was also considerable variation in the toxicity between the assay types (in vitro or in vivo). As many as 32.8% of the plants demonstrated some level of toxicity in vitro meanwhile 26.7% were toxic in vivo. Since it is customary to evaluate toxicity at the in vitro level and toxic plants are discarded before in vivo evaluation, that may explain while fewer plants were toxic in vivo. Toxicity varied within the same plant species from study to study and could be attributed to differences in the study design as well as differences in the parts of the plants used for testing. From this study, the most toxicity was observed with the leaves. Also, a relationship could be established between toxicity and antiplasmodial activity; as the activity of the plant increases, the toxicity, on the other hand, was observed to decrease. Furthermore, albino mice and Vero E6 cells were the most commonly used assays in the evaluation of toxicity. Unfortunately, the authors could nt make a meaningful relationship between the type of assay and toxicity because of the fewer studies assessing the toxicity of the medicinal plants.

This study, however, is limited in that the analyses may have been compounded by the substantial inter-study variation in the methodologies used by different independent studies for the extraction of plant material, the overall extraction yield, the diversity of extracted metabolites as well as the geographical variations in the different sites used in the plant collection. However, the study has provided important baseline data that may be exploited by researchers in the field for the discovery and development of new anti-malarial drugs.

## Conclusion

This study has revealed the slothful progress in the discovery and development of new anti-malarial drugs from African medicinal plants. Despite the encouraging activities demonstrated by the plants in vitro, fewer plants have been evaluated in vivo and just one clinical trial has been conducted so far with *Cochlospermum planchonii* (*Bixaceae*). The study also revealed considerable inter-study variation in the antiplasmodial activities of the plants, however, the activity of some plants including *Alchornea cordifolia, Azadirachta indica,* and *Zanthoxylum chalybeum* was consistently very good. The study demonstrates a relationship between antiplasmodial activity and toxicity whereby the toxicity of the plants decreases as the antiplasmodial activity increases. Besides, the active compounds were identified in just a handful of the plants. Therefore, there is a need for a policy-driven approach in the discovery and development of new anti-malarial drugs to subvert the rising resistance to the frontline anti-malarial drugs in the world.

## Supplementary Information

**Additional file 1: Table S1.** In vitro and in vivo studies reporting inactive antiplasmodial or antimalarial activity.** Table S2.** List of active compounds identified from plants.

## Data Availability

Not applicable.

## Abbreviations

PRISMA: Preferred Reporting Items for Systematic Reviews and Meta-analysis
Nd: Not done
Ns: Not specified
SI: Selectivity Index
LD_50_: Median lethal dose
IC_50_: Half-maximal inhibitory concentration
CC_50_: 50% Cytotoxic concentration
LC_50_: Lethal concentration
