# Supplementary material for: Antiplasmodial, antimalarial activities and toxicity of African medicinal plants: a systematic review of literature
Source: Malar J. 2021 Aug 25;20:349. doi: 10.1186/s12936-021-03866-0 (PMC8390284; doi:10.1186/s12936-021-03866-0)
Supplement: Supplementary file 1 — Additional file 1: Table S1. In vitro and in vivo studies reporting inactive antiplasmodial or antimalarial activity. Table S2. List of active compounds identified from plants. [file 12936_2021_3866_MOESM1_ESM.docx]

**Supplementary Table 1: *In vitro* and *in vivo* studies reporting inactive antiplasmodial or antimalarial activity**

| **Plant species** | ***Plant family*** | **Source** | **Country of study** | **Part of plant used** | **Assay type** | **Extraction solvent** | **IC_50_ or ED_50_ or LD50** | **Strain of *Plasmodium* Tested** | **Parasite reduction rate** | **Toxicity (value; assay)** |
| --- | --- | --- | --- | --- | --- | --- | --- | --- | --- | --- |
| *Mangifera indica* | *Anacardiaceae* | [26] | Kenya | Stem barks | *In vitro* | Methanol | <25 µg/ml IC_50_ | *Plasmodium falciparum* D6, W2 | Nd | Nd |
| *Ricinus communis* | *Euphorbiaceae* | [26] | Kenya | Leaves | *In vitro* | Methanol | <25 µg/ml IC_50_ | *Plasmodium falciparum* D6, W2 | Nd | Nd |
| *Cuminum cyminum L.* | *Apiaceae* | [214] | Thailand | Seeds | *In vitro* | Ethanol | 23.9-26.5 µg/ml IC_50_ | *Plasmodium falciparum* 3D7, K1 | Nd | Nd |
| *Acacia nilotica* | *Fabaceae* | [142] | Nigeria | Leaves | *In vitro* | Chloroform | Nd | *Plasmodium berghei* | 40% at 100 mg/kg/day | Nd |
| *Acacia seyal Del.* | *Fabaceae* | [168] | Kenya | Roots | *In vivo* | Water | Nd | *Plasmodium berghei* | 27.8% at 10 mg/kg/day | No |
| *Acacia tortilis* | *Fabaceae* | [124] | Kenya | Ns | *In vitro* | Methanol | 85.73 µg/ml IC_50_ | *Plasmodium falciparum* D6, W2 | Nd | Nd |
| *Acalypha wilkinsiana* | *Euphorbiaceae* | [214] | Nigeria | Leaves | *In vivo* | Water, chloroform | Nd | *Plasmodium berghei* | 47.24% at 440 mg/kg/day | Nd |
| *Acanthospermum hispidum dc* | *Asteraceae* | [23] | Sudan | Aerial parts | *In vitro* | Methanol | 4.9-48 µg/ml IC_50_ | *Plasmodium falciparum* 3D7, Dd2 | Nd | No |
| *Acokanthera schimperi* | *Apocynaceae* | [215] | Ethiopia | Leaves | *In vivo* | Methanol | Nd | *Plasmodium berghei* ANKA | 34.8% at 600 mg/kg/day | No |
| *Ageratum conyzoides* | *Asteraceae* | [22] | South Africa | Whole plant | *In vitro* | Dichloromethane/methanol | 27µg/ml IC_50_ | *Plasmodium falciparum* D10 | Nd | Nd |
| *Ajuga remota* | *Lamiaceae* | [216] | Kenya | Aerial parts | *In vitro* | Ethanol | 55 – 57 µg/ml IC_50_ | *Plasmodium falciparum* FCA/20GHA, W2 | Nd | Nd |
| *Albizia coriaria* | *Fabaceae* | [124] | Kenya | Ns | *In vitro* | Methanol | 71.17 µg/ml IC_50_ | *Plasmodium falciparum* D6 & W2 | Nd | Nd |
| *Alchornea cordifolia* | *Euphorbiaceae* | [43] | Ivory Coast | Leaves | *In vitro* | Ethanol | 22.3 µg/ml IC_50_ | *Plasmodium falciparum* FCB1 | Nd | Nd |
| *Alchornea floribunda* | *Euphorbiaceae* | [40] | D.R. Congo | Root barks | *In vitro* | Water | 20.8 µg/ml IC_50_ | *Plasmodium falciparum* K1 | Nd | No |
| *Aloe debrana* | *Xanthorrhoeaceae* | [198] | Ethiopia | Leaves | *In vivo* | Methanol | Nd | *Plasmodium beghei* | 30.21% at 200 mg/kg/day | No |
| *Aloe humilis* | *Xanthorrhoeaceae* | [74] | S. Tome´ and prıncipe | Leave saps | *In vitro* | Ethyl acetate | 25 µg/ml IC_50_ | *Plasmodium falciparum* 3D7 and Dd2 | Nd | Nd |
| *Aloe nyeriensis var kedongensis* | *Xanthorrhoeaceae* | [124] | Kenya | Ns | *In vitro* | Water | 67.84-87.7 µg/ml IC_50_ | *Plasmodium falciparum* D6 & W2 | Nd | Nd |
| *Aloe sp.* | *Xanthorrhoeaceae* | [217] | Ethiopia | Leaves | *In vivo* | Ethanol | Nd | *Plasmodium berghei* | 73.945 at 650 mg/kg/day | Nd |
| *Amaranthus spinosus L* | *Amaranthaceae* | [218] | Burkina Faso | Stems | *In vivo* | Water | 789 mg/kg ED_50_ | *Plasmodium berghei berghei* | Nd | No |
| *Annona muricata* | *Annonaceae* | [108] | Togo | Leaves | *In vitro* | Ethanol | 39.9 µg/ml IC_50_ | *Plasmodium falciparum* | 87.6% at 100 ug | Nd |
| *Annona squamosa* | *Annonaceae* | [59] | Sudan | Leaves | *In vitro* | Methanol | 2-30 µg/ml IC_50_ | *Plasmodium falciparum* 3D7, Dd8 | Nd | Nd |
| *Antidesma laciniatum* | *Phyllanthaceae* | [98] | Cameroon | Leaves | *In vitro* | Water | 29.4 µg/ml* IC_50_ | *Plasmodium falciparum* W4 | Nd | Nd |
| *Artocarpus altilis* | *Moraceae* | [219] | Nigeria | Stem barks | *In vivo* | Ethanol | 227.2 mg/kg ED_50_ | *Plasmodium beghei* | 55.5% at 400 mg/kg/day | Nd |
| *Asparagus africanus* | *Asparagaceae* | [220] | Ethiopia | Roots, aerial parts | *In vivo* | Hydroalcohol | Nd | *Plasmodium beghei* | 46.1% at 600 mg/kg/day | Nd |
| *Asparagus racemosus* | *Asparagaceae* | [124] | Kenya | Ns | *In vitro* | Methanol | 32.63-33.95, 71 µg/ml IC_50_ | *Plasmodium falciparum* D6 & W2 | Nd | Nd |
| *Azadirachta indica* | *Meliaceae* | [217] | Ethiopia | Leaves | *In vivo* | Ethanol | Nd | *Plasmodium berghei* | 54.79% at 650 mg/kg/day | Nd |
|  |  | [168] | Kenya | Root barks | *In vivo* | Water | Nd | *Plasmodium berghei* | 3.1% at 10 mg/kg/day | Yes (LC_50_=285.8 µg/ml; Brine shrimp lethality test) |
|  |  | [221] | Burkina faso | Fruit | *In vivo* | Water | Nd | *Plasmodium berghei* ANKA | 45% at 200 mg/kg/day | Nd |
| *Balanites aegyptiaca* | *Zygophyllaceae* | [222] | Togo | Aerial parts | *In vitro* | Methanol | 24.56 μg/mL IC_50_ | *Plasmodium falciparum* | Nd | Nd |
| *Bauhinia rufescens* | *Fabaceae* | [223] | Burkina faso | Leaves | *In vivo* | Water | Nd | *Plasmodium beghei* | 25.7% at 500 mg/kg/day | Nd |
| *Bersama abyssinica* | *Melianthaceae* | [28] | Ivory Coast | Leaves | *In vitro* | Ethanol | 23.9 µg/ml IC_50_ | *Plasmodium falciparum* FcB1/Colombia strain | Nd | Nd |
|  |  | [43] | Ivory Coast | Leaves | *In vitro* | Ethanol | 20.7 µg/ml IC_50_ | *Plasmodium falciparum* FCB1 | Nd | Nd |
| *Boerhavia erecta* | *Nyctagynaceae* | [218] | Burkina Faso | Stems | *In vivo* | Water | 564 mg/kg ED_50_ | *Plasmodium berghei berghei* | Nd | No |
| *Boscia angustifolia* | *Capparaceae* | [121] | Mali | Leaves | *In vitro* | Methanol | 37.6 µg/ml IC_50_ | *Plasmodium falciparum* 3D7 | Nd | Nd |
| *Bridelia micrantha. Benth* | *Phyllanthaceae* | [224] | Nigeria | Ns | *In vitro* | Methanol | 158.7 µg/ml IC_50_ | *Plasmodium falciparum* K1 | Nd | Nd |
| *Brucea antidysenterica* | *Simaroubaceae* | [225] | Ethiopia | Seeds | *In vivo* | Methanol | Nd | *Plasmodium berghei* | 47.7% at 600 mg/kg/day | Yes (LD_50_=2000 mg/kg; albino mice) |
| *Caesalpinia bonducella* | *Fabaceae* | [226] | Tanzania | Leaves | *In vivo* | Ethanol | 42.8 mg/kg ID_50_ | *Plasmodium berghei* | Nd | No |
| *Caesalpinia volkensii* | *Fabaceae* | [216] | Kenya | Aerial parts | *In vitro* | Petroleum ether | 250 µg/ml IC_50_ | *Plasmodium falciparum* FCA/20GHA, W2 | Nd | Nd |
| *Canthium glaucum* | *Rubiaceae* | [136] | Kenya | Ns | *In vivo* | Trichloromethane+methanol | Nd | *Plasmodium berghei* | >40% | No |
|  |  | [136] | Kenya | Root | *In vivo* | Water | Nd | *Plasmodium berghei* | 31.98% at 100 mg/kg/day | No |
| *Cassia abbreviata* | *Caesalpiniceae* | [226] | Tanzania | Roots | *In vivo* | Ethanol | 111 mg/kg ID_50_ | *Plasmodium berghei* | Nd | No |
| *Cassia siamea* | *Caesalpiniaceae* | [103] | Congo brazzaville | Barks | *In vitro* | Dichloromethane | 21 µg/ml IC_50_ | *Plasmodium falciparum* FcM29-Cameroon | Nd | Nd |
| *Cissus quadrangularis* | *Vitaceae* | [121] | Mali | Whole plant | *In vitro* | Dicloremethane | 23.9 µg/ml IC_50_ | *Plasmodium falciparum* 3D7 | Nd | Nd |
| *Rotheca myricoides* | *Lamiaceae* | [188] | Kenya | Root barks | *In vivo* | Methanol | Nd | *Plasmodium berghei* NK65 | 31.7% at 500 mg/kg/day | Nd |
| *Cochlospermum planchonii* | *Bixaceae* | [221] | Burkina faso | Roots | *In vivo* | Water | Nd | *Plasmodium berghei* ANKA | 45.5% at 200 mg/kg/day | Nd |
| *Cogniauxia podolaena* | *Cucurbitaceae* | [103] | Congo brazzaville | Root | *In vitro* | Dichloromethane | 21 µg/ml IC_50_ | *Plasmodium falciparum* FcM29-Cameroon | Nd | Nd |
| *Combretum glutinosum* | *Combretaceae* | [123] | Senegal | Barkss | *In vitro* | Methanol, water | 35 µg/ml IC_50_ | *Plasmodium falciparum* | Nd | Nd |
| *Crinum macowanii* | *Amaryllidaceae* | [22] | South Africa | Bulbs | *In vitro* | Water | 25 µg/ml IC_50_ | *Plasmodium falciparum* D10 | Nd | Nd |
| *Croton macrostachyus* | *Euphorbiaceae* | [215] | Ethiopia | Leaves | *In vivo* | Methanol | Nd | *Plasmodium berghei* ANKA | 34.33% at 600 mg/kg/day | No |
| *Dialium guineense* | *Fabaceae* | [71] | Togo | Twigs and Leaves | *In vitro* | Water | <22.5 µg/ml IC_50_ | *Plasmodium falciparum* | Nd | Nd |
| *Dichapetalum guineense* | *Dichapetalaceae* | [71] | Togo | Aerial parts | *In vitro* | Water | <22.5 µg/ml IC50 | *Plasmodium falciparum* | Nd | Nd |
| *Dichrostachys cinerea (L)* | *Fabaceae* | [168] | Kenya | Root | *In vivo* | Water | Nd | *Plasmodium berghei* | 6.3% at 10 mg/kg/day | No |
| *Diplorhynchus condylocarpon* | *Apocynaceae* | [22] | South Africa | Roots | *In vitro* | Dichloromethane/methanol | 24 µg/ml IC_50_ | *Plasmodium falciparum* D10 | Nd | Nd |
| *Dodonaea angustifolia* | *Sapindaceae* | [227] | Ethiopia | Seeds | *In vivo* | Water, hyDRoalcohol (80% water 20% methanol) | Nd | *Plasmodium beghei* | 35.79% at 400 mg/kg/day | Nd |
| *Echinops kebericho mesfin* | *Asteraceae* | [228] | Ethiopia | Rhizome | *In vivo* | Methanol | Nd | *Plasmodium beghei* ANKA | 32.83% at 1000 mg/kg | No |
| *Elephantorrhiza elephantina* | *Fabaceae* | [22] | South Africa | Leaves | *In vitro* | Dichloromethane/methanol | 26 µg/ml IC_50_ | *Plasmodium falciparum* D10 | Nd | Nd |
| *Emilia discifolia* | *Asteraceae* | [57] | Uganda | Shoots | *In vitro* | Ethyl acetate | 24.7-30.2 µg/ml IC_50_ | *Plasmodium falciparum* D10, K1 | Nd | Nd |
| *Enantia chlorantha* | *Annonaceae* | [229] | Nigeria | Barks | *In vivo* | Ethanol | 0·34 mg/g ED_50_ | *Plasmodium yoelii* | Nd | Nd |
| *Erythrina senegalensis. DC* | *Fabaceae* | [224] | Nigeria | Ns | *In vitro* | Methanol | 99.7  µg/ml IC_50_ | *Plasmodium falciparum* K1 | Nd | Nd |
| *Eucalyptus globulus* | *Myrtaceae* | [81] | Cameroon | Leaves | *In vitro* | Dichloromethane/methanol | 16.80-26.45 µg/ml IC_50_ | *Plasmodium falciparum* 3D7, DD2 | Nd | No |
| *Euphorbia hirta* | *Euphorbiaceae* | [230] | Nigeria | Whole plant | *In vivo* | Ethanol | Nd | *Plasmodium berghei* | 44.36% at 400 mg/kg | Nd |
| *Ficus polita* | *Moraceae* | [108] | Togo | Leaves | *In vitro* | Ethanol | 20.8µg/ml IC50 | *Plasmodium falciparum* | 77.2% at 100 ug | Nd |
| *Ficus sur* | *Moraceae* | [188] | Kenya | Root barks | *In vivo* | Methanol | Nd | *Plasmodium berghei* NK68 | 48.8% at 500 mg/kg/day | Nd |
| *Garcinia buchananii* | *Clausiaceaem* | [232] | Kenya | Whole | *In vivo* | Methanol | 169.4 mg/kg EC_50_ | *Plasmodium berghei* ANKA | 54.62% at 400 mg/kg | Nd |
| *Gardenia jovis tantis* | *Rubiaceae* | [23] | Sudan | Leaves | *In vitro* | Methanol | 5.3-52 µg/ml IC_50_ | *Plasmodium falciparum* 3D7, Dd2 | Nd | No |
| *Gardenia ternifolia* | *Rubiaceae* | [233] | Ethiopia | Root barks | *In vivo* | Methanol | Nd | *Plasmodiumbeghei* | 51.33% at 600 mg/dl | No |
| *Gomphocarpus fruticosus* | *Apocynaceae* | [22] | South Africa | Fruit | *In vitro* | Dichloromethane/methanol | 26 µg/ml IC50 | *Plasmodium falciparum* D10 | Nd | Nd |
| *Guiera senegalensis* | *Combretaceae* | [223] | Burkina faso | Leaves | *In vivo* | Water |  | *Plasmodium beghei* | 44.9% at 500 mg/kg/day | Nd |
| *Indigofera emarginella* | *Fabaceae* | [57] | Uganda | Shoots | *In vitro* | Methanol | 22.4-22.5 µg/ml IC_50_ | *Plasmodium falciparum* D10, K1 | Nd | Nd |
| *Irvingia gabonensis* | *Irvingiaceae* | [28] | Ivory Coast | Stem barks | *In vitro* | Ethanol | 21.6 µg/ml IC_50_ | *Plasmodium falciparum* FcB1/Colombia strain | Nd | Nd |
|  |  | [43] | Ivory Coast | Stem barks | *In vitro* | Ethanol | 22.1 µg/ml IC_50_ | *Plasmodium falciparum* FCB1 | Nd | Nd |
| *Jatropha gossypiifolia* | *Euphorbiaceae* | [71] | Togo | Leaves | *In vitro* | Water | <22.5 µg/ml IC_50_ | *Plasmodium falciparum* | Nd | Nd |
|  |  | [63] | Burkina faso | Leaves | *In vitro* | Dichloromethane | 35.66 µg/ml IC_50_ | *Plasmodium falciparum* 3D7 &W2 | Nd | Nd |
| *Jatropha multifida* | *Euphorbiaceae* | [113] | Nigeria | Stem barks | *In vitro* | Methanol | 7 231.5 µg/mL IC_50_ | *Plasmodium falciparum* | Nd | Nd |
| *Justicia flava* | *Acanthaceae* | [22] | South Africa | Whole plant | *In vitro* | Dichloromethane/methanol | 31 µg/ml IC_50_ | *Plasmodium falciparum* D10 | Nd | Nd |
| *Keetia leucantha* | *Rubiaceae* | [233] | Benin | Twigs | *In vivo* | Dichloromethane, water | Nd | *Plasmodium berghei* | 27.8% at 50 mg/kg/day | No |
| *Khaya senegalensis* | *Meliaceae* | [59] | Sudan | Leaves | *In vitro* | Methanol | 5.5-47 µg/ml IC_50_ | *Plasmodium falciparum* 3D7, Dd4 | Nd | Nd |
| *Lannea discolor* | *Anacardiaceae* | [22] | South Africa | Fruits | *In vitro* | Dichloromethane | 25 µg/ml IC_50_ | *Plasmodium falciparum* D10 | Nd | Nd |
| *Launaea cornuta* | *Asteraceae* | [135] | Kenya | Ns | *In vivo* | Methanol | Nd | *Plasmodium berghei* | 39% at 100 mg/kg/day | Yes (LD_50_<1000 µg/ml; brine shrimp) |
|  | *Asteraceae* | [135] | Kenya | Leaves | *In vivo* | Water | Nd | *Plasmodium berghei* | 38.13% at 100 mg/kg/day | Yes (LD_50_<1000 µg/ml; Brine shrimp lethality test) |
| *Luffa aegyptiaca* | *Cucurbitaceae* | [108] | Togo | Leaves | *In vitro* | Ethanol | 88.0 µg/ml IC_50_ | *Plasmodium falciparum* | 88.0% at 100 ug | Nd |
| *Mammea africana* | *Calophyllaceae* | [40] | D.R. Congo | Stem barks | *In vitro* | Water | 28.57 µg/ml IC_50_ | *Plasmodium falciparum* K1 | Nd | No |
| *Manniophyton fulvum* | *Euphorbiaceae* | [40] | D.R. Congo | Leaves | *In vitro* | Water | 22.44 µg/ml IC_50_ | *Plasmodium falciparum* K1 | Nd | No |
| *Maranthes floribunda* | *Chrysobalanaceae* | [226] | Tanzania | Roots | *In vivo* | Ethanol | > 2400 mg/kg IC_50_ | *Plasmodium berghei* | Nd | No |
| *Mareya micrantha* | *Euphorbiaceae* | [28] | Ivory Coast | Stem barks | *In vitro* | Ethanol | 27.6 µg/ml IC_50_ | *Plasmodium falciparum* FcB1/Colombia strain | Nd | Nd |
| *Markhamia tomentosa* | *Bignoniaceae* | [234] | Nigeria | Leaves | *In vivo* | Water | Nd | *Plasmodium beghei* | 73% at 800 mg/kg/day | Nd |
| *Maytenus acuminata* | *Celastraceae* | [188] | Kenya | Root barks | *In vivo* | Methanol | Nd | *Plasmodium berghei* NK69 | 41.5% at 500 mg/kg/day | Nd |
| *Melanthera scandens* | *Asteraceae* | [43] | Ivory Coast | Whole plant | *In vitro* | Ethanol | 20.7 µg/ml IC_50_ | *Plasmodium falciparum* FCB1 | Nd | Nd |
| *Mezoneuron benthamianum* | *Fabaceae* | [235] | Guinea | Leaves | *In vitro* | Ethanol | 22.5 - 32.6 µg/ml IC_50_ | *Plasmodium falciparum* 3D7 | Nd | Nd |
| *Millettia versicolor* | *Fabaceae* | [103] | Congo brazzaville | Leaves | *In vitro* | Ethanol | 33 µg/ml IC_50_ | *Plasmodium falciparum* FcM29-Cameroon | Nd | Nd |
| *Mitracarpus scaber* | *Rubiaceae* | [64] | Senegal | Leaves | *In vitro, in vivo* | Acetone | 10-38 µg/ml IC50 | *Plasmodium falciparum* FcM29, FcB1, *Plasmodium vinckei petteri* | Nd | Yes (SI=0.4; mouse mammary tumour [Fm3A]) |
| *Momordica charantia* | *Cucurbitaceae* | [108] | Togo | Leaves | *In vitro* | Ethanol | 79.1 µg/ml IC_50_ | *Plasmodium falciparum* | 68.4% at 100 ug | Nd |
| *Morinda morindoides* | *Rubiaceae* | [162] | Congo | Leaves | *In vivo* | Dichloromethane | Nd | *Plasmodium falciparum, Plasmodium berghei* | 44.21 at 400 mg/kg/day | Nd |
|  |  | [70] | D.R. Congo | Leaves | *In vitro* | Petroleum ether, isoamylalcohol, ethanol, chloroform | 1.8-94.2 µg/ml IC_50_ | *Plasmodium falciparum* | Nd | Nd |
| *Moringa pterygosperma* | *Moringaceae* | [108] | Togo | Twigs and Leaves | *In vitro* | Ethanol | 60 µg/ml IC_50_ | *Plasmodium falciparum* | 87.0% at 100 ug | Nd |
| *Murraya koenigii* | *Rutaceae* | [219] | Nigeria | Leave | *In vivo* | Ethanol | 287.1 mg/kg ED50 | *Plasmodium beghei* | 48.5 at 400mg/kg | Nd |
| *Nauclea latifolia* | *Rubiaceae* | [219] | Nigeria | Roots | *In vivo* | Ethanol | 279.3mg/kg ED_50_ | *Plasmodium beghei* | 34.4 at 400 mg/kg | Nd |
|  |  | [71] | Togo | Roots | *In vitro* | Water | <22.5 µg/ml IC_50_ | *Plasmodium falciparum* | Nd | Nd |
|  |  | [236] | Nigeria | Leaves | *In vivo* | Methanol | Nd | *Plasmodium berghei* ANKA | 56.% at 860 mg/kg/day | Yes (LD_50_=4.3 g/kg; albino mice) |
| *Nauclea pobeguinii* | *Rubiaceae* | [237] | D.R. Congo | Stem barks | *Human* | Ethanol | Nd | *Plasmodium falciparum* | 87.9% at 1000 mg/kg/day | Nd |
| *Nigella sativa* | *Ranunculaceae* | [237] | Nigeria | Seeds | *In vivo* | Methanol | Nd | *Plasmodium yoelii nigeriensis* | 94% at 1250 mg/kg | Nd |
| *Ocimum lamiifolium* | *Lamiaceae* | [225] | Ethiopia | Leaves | *In vivo* | Water | Nd | *Plasmodium berghei* | 35.53% at 600 mgkg/day | No |
| *Ocotea usambarensis* | *Lauraceae* | [199] | Kenya | Stem barks | *0* | Methanol | 7.69-29.75 µg/ml IC_50_ | *Plasmodium falciparum* D6 & W2, Plasmodium berghei ANKA | 42.2% at 100 mg/kg/day | No |
| *Opilia celtidifolia* | *Opiliaceae* | [110] | Togo | Aerial parts | *In vitro* | Water | 83.176 µg/ml IC_50_ | *Plasmodium falciparum* | Nd | Nd |
| *Osyris quadripartita* | *Santalaceae* | [239] | Ethiopia | Leaves | *In vivo* | Chloroform | Nd | *Plasmodium berghei* ANKA | 41.3% at 600 mg/kg/day | No |
| *Parkinsonia aculeata* | *Fabaceae* | [69] | Mozambique and Portugal | Aerial parts | *In vitro* | N-hexane | 24.5 µg/ml IC_50_ | *Plasmodium falciparum* 3D8 | Nd | Nd |
| *Parquetina nigrescens* | *Apocynaceae* | [28] | Ivory Coast | Leaves | *In vitro* | Ethanol | 21.2 µg/ml IC_50_ | *Plasmodium falciparum* FcB1/Colombia strain | Nd | Nd |
|  |  | [43] | Ivory Coast | Leaves | *In vitro* | Ethanol | 20.3 µg/ml IC_50_ | *Plasmodium falciparum* FCB1 | Nd | Nd |
| *Paullinia pinnata* | *Sapindaceae* | [71] | Togo | Aerial parts | *In vitro* | Water | <22.5 µg/ml IC_50_ | *Plasmodium falciparum* | Nd | Nd |
| *Penianthus longifolius* | *Menispermaceae* | [40] | D.R. Congo | Root barks | *In vitro* | Water | 27.1 µg/ml IC_50_ | *Plasmodium falciparum* K1 | Nd | No |
| *Pennisetum polystachion* | *Poaceae* | [123] | Senegal | Whole aerial | *In vitro* | Methanol, water | 28 µg/ml IC_50_ | *Plasmodium falciparum* | Nd | Nd |
| *Pericopsis elata. Harms* | *Fabaceae* | [224] | Nigeria | Ns | *In vitro* | Methanol | 124.8  µg/ml IC_50_ | *Plasmodium falciparum* K1 | Nd | Nd |
| *Piliostigma thonningii* | *Fabaceae* | [22] | South Africa | Twigs | *In vitro* | Dichloromethane/methanol | 25.9 µg/ml IC_50_ | *Plasmodium falciparum* D10 | Nd | Nd |
| *Piper guineense* | *Piperaceae* | [240] | Nigeria | Leaves | *In vivo* | Ethanol | Nd | *Plasmodium berghei* | 62.69% at 600 mg/kg/day | No |
| *Polyalthia longifolia* | *Annonaceae* | [234] | Nigeria | Leaves | *In vivo* | Water | Nd | *Plasmodium berghei* | 53% at 800 mg/kg/day | Nd |
| *Pseudocedrela kotosifye* | *Meliaceae* | [59] | Sudan | Leaves | *In vitro* | Methanol | 15-50 µg/ml IC_50_ | *Plasmodium falciparum* 3D7, Dd7 | Nd | Nd |
| *Pseudospondias microcarpa* | *Anacardiaceae* | [103] | Congo brazzaville | Leaves | *In vitro* | Ethanol | 26 µg/ml IC_50_ | *Plasmodium falciparum* FcM29-Cameroon | Nd | Nd |
| *Rauvolfia caffra Sond.* | *Apocynaceae* | [22] | South Africa | Fruits | *In vitro* | Dichloromethane | 26.5µg/ml IC_50_ | *Plasmodium falciparum* D10 | Nd | Nd |
| *Rhamnus prinoides* | *Rhamnaceae* | [231] | Kenya | Whole plant | *In vivo* | Methanol | 139.2 mg/kg EC_50_ | *Plasmodium berghei* (ANKA) | 57.74% at 400 mg/kg/day | Nd |
|  |  | [188] | Kenya | Leaves | *In vivo* | Methanol | Nd | *Plasmodium berghei* NK70 | 43.9% at 500 mg/kg/day | Nd |
| *Rhamnus staddo* | *Rhamnaceae* | [188] | Kenya | Root barks | *In vivo* | Methanol | Nd | *Plasmodium berghei* NK71 | 48.1% at 500 mg/kg/day | Nd |
| *Rubus keniensis* | *Rosaceae* | [231] | Kenya | Whole | *In vivo* | Methanol | 245.1 mg/kg EC_50_ | *Plasmodium berghei* (ANKA) | 44.12% at 400 mg/kg/day | Nd |
| *Salvia africana-caerulea* | *Lamiaceae* | [120] | South Africa | Aerial parts | *In vitro* | Methanol/chloroform | 22.68 71 µg/ml IC_50_ | *Plasmodium falciparum* FCR-3 | Nd | Nd |
| *Salvia disermas* | *Lamiaceae* | [120] | South Africa | Aerial parts | *In vitro* | Methanol/chloroform | 24.1771 µg/ml IC_50_ | *Plasmodium falciparum* FCR-3 | Nd | Nd |
| *Salvia lanceolata* | *Lamiaceae* | [120] | South Africa | Aerial parts | *In vitro* | Methanol/chloroform | 26.0171 µg/ml IC_50_ | *Plasmodium falciparum* FCR-3 | Nd | Yes (IC_50_=26.71 µg/ml; kidney cells) |
| *Salvia namaensis* | *Lamiaceae* | [120] | South Africa | Aerial parts | *In vitro* | Methanol/chloroform | 25.3871 µg/ml IC_50_ | *Plasmodium falciparum* FCR-3 | Nd | Nd |
| *Salvia verbenaca* | *Lamiaceae* | [120] | South Africa | Aerial parts | *In vitro* | Methanol/chloroform | 23.9771 µg/ml IC_50_ | *Plasmodium falciparum* FCR-3 | Nd | Nd |
| *Scherbournia bignoniiflora* | *Rubiaceae* | [43] | Ivory Coast | Leaves | *In vitro* | Ethanol | 24.7 µg/ml IC_50_ | *Plasmodium falciparum* FCB1 | Nd | Nd |
| *Schumanniophyton magnificum* | *Rubiaceae* | [89] | Cameroon | Stem barks | *In vitro* | Water/ethanol | 25.5 µg/ml IC_50_ | *Plasmodium falciparum* W2 | Nd | Nd |
| *Senna petersiana (Bolle) Lock.* | *Fabaceae* | [37] | South Africa | Leaves | *In vitro* | Dichloromethane | 22.5 µg/ml IC_50_ | *Plasmodium falciparum* NF54 | Nd | Nd |
| *Senna singueana* | *Fabaceae* | [241] | Ethiopia | Leaves | *In vivo* | Ethanol | Nd | *Plasmodium berghei* ANKA | 44.52% at 400 mg/kg/day | Nd |
| *Strophanthus eminii* | *Apocynaceae* | [226] | Tanzania | Roots | *In vivo* | Ethanol | > 2400 mg/kg | *Plasmodium berghei* | Nd | No |
| *Strychnos spinosa* | *Loganiaceae* | [28] | Ivory Coast | Barks | *In vitro* | Ethanol | 21.8 µg/ml IC_50_ | *Plasmodium falciparum* FcB1/Colombia strain | Nd | Nd |
| *Stylosanthes erecta* | *Fabaceae* | [121] | Mali | Aerial parts | *In vitro* | Dichloromethane | 21.9 µg/ml IC_50_ | *Plasmodium falciparum* 3D7 | Nd | Nd |
| *Syzygium guineense* | *Myrtaceae* | [242] | Ethiopia | Leaves | *In vivo* | Water | Nd | *Plasmodium berghei* | 49.09% at 400 mg/kg | Nd |
| *Tabernaemontana elegans* | *Apocynaceae* | [69] | Mozambique and Portugal | Leaves | *In vitro* | Dichloromethane | 26.9 µg/ml IC_50_ | *Plasmodium falciparum* 3D9 | Nd | Nd |
| *Tamarindus indica* | *Fabaceae* | [217] | Ethiopia | Fruit | *In vivo* | Water | Nd | *Plasmodium berghei* | 81.09% st 650 mg/kg/day | Nd |
|  |  | [168] | Kenya | Stem barks | *In vivo* | Water | Nd | *Plasmodium berghei* | 25.1% at 10 mg/kg/day | Yes (LC_50_=516.4 µg/ml; Brine shrimp lethality test) |
| *Thomandersia hensii* | *Schlegeliaceae* | [89] | Cameroon | Stem barks | *In vitro* | Ethyl acetate | 24.7 µg/ml IC_50_ | *Plasmodium falciparum* W2 | Nd | Nd |
| *Tragia fuliaris* | *Euphorbiaceae* | [226] | Tanzania | Roots | *In vivo* | Ethanol | 639.3 mg/kg ID_50_ | *Plasmodium berghei* | Nd | No |
| *Trichilia heudelotii* | *Meliaceae* | [234] | Nigeria | Stem | *In vivo* | Water | Nd | *Plasmodium berghei* | 40% at 800 mg/kg/day | Nd |
| *Uvaria chamae P. Beauv* | *Annonaceae* | [243] | Nigeria | Leaves | *In vivo* | Methanol | Nd | *Plasmodium berghei* NK65 | 42% at 800 mg/kg/day | Nd |
| *Vangueria acutiloba* | *Rubiaceae* | [199] | Kenya | Stem barks | *In vitro* | Methanol | 13.36-33.98 µg/ml IC_50_ | *Plasmodium falciparum* D6 &W2, *Plasmodium berghei* ANKA | 26.01% at 100 mg/kg/day | No |
| *Vepris glomerata* | *Rutaceae* | [226] | Tanzania | Roots | *In vivo* | Ethanol | > 2400 mg/kg IC50 | Plasmodiumberghei | Nd | No |
| *Vernonia amygdalina* | *Asteraceae* | [244] | Uganda | Ns | *In vitro* | Acetone/water | 25.5 µg/ml IC_50_ | *Plasmodium falciparum* | Nd | Nd |
|  |  | [245] | Nigeria | Leaves | *In vivo* | Ethanol | Nd | *Plasmodium berghei* ANKA | 82.3% at 1000 mg/kg/day | Nd |
|  |  | [246] | Uganda | Leaves | *In vivo* | Water | Nd | *Plasmodium falciparum* | 32% at 0.5 g/kg/day | No |
|  |  | [247] | Nigeria | Leaves | *In vivo* | Ethanol | Nd | *Plasmodium berghei* | 37.85% at 1000 mg/kg | Nd |
| *Vernonia brazzavillensis* | *Asteraceae* | [103] | Congo brazzaville | Leaves | *In vitro* | Ethanol | 22 µg/ml IC_50_ | *Plasmodium falciparum* FcM29-Cameroon | Nd | Nd |
| *Vitellaria paradoxa* | *Sapotaceae* | [63] | Burkina faso | Barks | *In vitro* | Dichloromethane | 43.94 µg/ml IC_50_ | *Plasmodium falciparum* 3D7 & W2 | Nd | Nd |
| *Waltheria indica* | *Malvaceae* | [63] | Burkina faso | Roots | *In vitro* | Dichloromethane | 29.71 µg/ml IC_50_ | *Plasmodium falciparum* 3D7 & W2 | Nd | Nd |
| *Zanthoxylum chalybeum* | *Rutaceae* | [135] | Kenya | Stem barks | *In vivo* | Water | Nd | *Plasmodium berghei* | 44.93% 100 mg/kg/day | Yes (LD_50_<1000 µg/ml; brine shrimp) |
| *Zingiber officinale Roscoea* | *Zingiberaceae* | [228] | Ethiopia | Roots | *In vivo* | Methanol | Nd | *Plasmodium beghei* ANKA | 49.43% at 1000 mg/kg/day | No |

**Nd: Not done**

**Ns: Not specified**

**SI: Selectivity index**

***Activity determined using pure compounds isolated from plant**

Supplementary Table 2: List of active compounds identified from plants

| ***Plant species*** | **Plant family** | **Source** | **Active compounds identified** |
| --- | --- | --- | --- |
| ***Nauclea pobeguinii*** | *Rubiaceae* | [107] | (5S)-5-carboxystrictosidine, 19-O-methylangustoline, 3-O-β-fucosylquinovic acid, 3-ketoquinovic acid and strictosamide |
| ***Kniphofia foliosa*** | *Xanthorrhoeaceae* | [102] | 10‐(chrysophanol‐7′‐yl)‐10‐(ξ)‐hydroxychrysopanol‐9‐anthrone and chryslandicin |
| ***Toddalia asiatica*** | *Rutaceae* | [125] | Alkaloid Nitidine |
| ***Zanthoxylum tsihanimposa*** | *Rutaceae* | [130] | Alkaloid γ-fagarine |
| ***Cassia sieberiana*** | *Fabaceae* | [153] | Alkaloids |
| ***Clerodendrum myricoides*** | *Lamiaceae* | [198] | Alkaloids |
| ***Calpurnia aurea*** | *Fabaceae* | [150] | Alkaloids, cardiac glycosides, flavonoids, phenols, phytosteriods, saponins, terpenoids and tannins |
| ***Momordica foetida*** | *Cucurbitaceae* | [77] | Alkaloids, flavonoids, cardiac glycosides |
| ***Chrysophyllum albidum*** | *Sapotaceae* | [156] | Alkaloids, flavonoids, saponins, tannins, cardiac glycosides |
| ***Balanites rotundifolia*** | *Zygophyllaceae* | [146] | Alkaloids, glycosides |
| ***Commiphora africana*** | *Burseraceae* | [159] | Alkaloids, steroids, cardiac glycosides, flavonoids, tannins, triterpenoids, saponins |
| ***Aloe pulcherrima*** | *Xanthorrhoeaceae* | [41] | Aloesaponarin II |
| ***Acanthospermum Hispidum DC*** | *Asteraceae* | [29] | Antioxidants, glycosides, coumarins and flavonoids |
| ***Salvia radula*** | *Lamiaceae* | [120] | Betulafolientriol oxide, (salvigenin |
| ***Sida acuta*** | *Malvaceae* | [38] | Cryptolepine |
| ***Cryptolepis sanguinolenta*** | *Apocynaceae* | [84] | Cryptolepine and isocryptolepine |
| ***Dacryodes edulis*** | *Burseraceae* | [85] | DES4-methyl 3,4,5-trihydroxybenzoate |
| ***Zanthoxylum heitzii*** | *Rutaceae* | [129] | Dihydronitidine (most active), pellitories and heitziquinone |
| ***Triphyophyllum peltatum*** | *Dioncophyllaceae* | [191] | Dioncopeltine A, dioncophylline C |
| ***Zanthoxylum chalybeum*** | *Rutaceae* | [77] | Faramide |
| ***Strychnos spinosa*** | *Loganiaceae* | [123] | Flavonoids, tannins, cardiotonic heterosides |
| ***Vangueria infausta Burch. subsp. Infausta*** | *Rubiaceae* | [37] | Friedeline, morindolide |
| ***Momordica balsamina*** | *Cucurbitaceae* | [68] | Karavoate B |
| ***Garcinia kola*** | *Clusiaceae* | [169] | Kolaviron |
| ***Dodonaea angustifolia*** | *Sapindaceae* | [198] | Quinins, saponins, flavonoides, alkaloids, terpenoids, diterpenoids, essential oils |
| ***Pavetta corymbosa*** | *Rubiaceae* | [110] | Saponins, alkaloids, flavonoids, tannins |
| ***Verbena hastata*** | *Verbenaceae* | [192] | Saponins, terpenes, flavonoids, sterols, carbohydrate |
| ***Dichrostachys cinerea*** | *Fabaceae* | [159] | Steroids, cardiac glycosides, flavonoids, tannins, triterpenoids, saponins |
| ***Tabernaemontana elegans Stapf.*** | *Apocynaceae* | [37] | Tabernaemontanine, dregamine |
| ***Tamarindus indica*** | *Fabaceae* | [110] | Tannins |
| ***Artemisia abyssinica*** | *Asteraceae* | [197] | Tannins, alkaloids, terpenoids, polyphenols, flavonoids |
| ***Microglossa pyrifolia*** | *Asteraceae* | [77] | Tannins, saponins, cardiac glycoside |
| ***Chrozophora senegalensis*** | *Euphorbiaceae* | [155] | Tannins, saponins, flavonoids, alkaloids, high fatty acids, and steroids glycosides |
| ***Clerodendrum rotundifolium*** | *Lamiaceae* | [77] | Tannins, saponins, flavonoids, cardiac glycosides |
